# First trimester maternal infections and offspring congenital heart defects: a meta-analysis

**DOI:** 10.1093/eurheartj/ehaf564

**Published:** 2026-02-18

**Authors:** Huimin Su, Edie Guo, Mark Woodward, Jian-Rong He, Tim Waterboer, Art Schuermans, Alexander Van De Bruaene, Els Troost, Pieter De Meester, Karl Morten, Terence Dwyer, Christina Chambers, Kazem Rahimi, Werner Budts, Nathalie Conrad

**Affiliations:** 1 Department of Cardiovascular Sciences, KU Leuven, Campus Gasthuisberg, Herestraat 49 Leuven 3000, Belgium; 2 Nuffield Department of Women’s and Reproductive Health, University of Oxford, Women’s Centre, John Radcliffe Hospital, Oxford OX3 9DU, UK; 3 The George Institute for Global Health, Imperial College London, London, UK; 4 The George Institute for Global Health, University of New South Wales, Sydney, Australia; 5 Division of Birth Cohort Study, Guangzhou Women and Children’s Medical Center, Guangzhou, China; 6 Infections and Cancer Epidemiology, German Cancer Research Center, Heidelberg, Germany; 7 Program in Medical and Population Genetics and Cardiovascular Disease Initiative, Broad Institute of MIT and Harvard, Cambridge, MA, USA; 8 Congenital and Structural Cardiology, University Hospitals Leuven, Leuven, Belgium; 9 Murdoch Children’s Research Institute, Royal Children’s Hospital, Melbourne, Australia; 10 Department of Pediatrics, University of California San Diego, San Diego, CA, USA

**Keywords:** Congenital heart defects, Maternal infections, Systematic review, Epidemiology

## Abstract

**Background and Aims:**

Maternal infections have been proposed to play a role in the development of congenital heart defects (CHD). This study aims to synthesize contemporary evidence on the association between first-trimester maternal infection and risk of off-spring CHD.

**Methods:**

This systematic review and meta-analysis (PROSPERO number: CRD42024523638) used Embase, PubMed, Web of Science, Scopus, and the Cochrane Library to identify studies investigating first-trimester maternal infection and offspring CHD, published up until 30 September 2024. Human studies with a minimum of 50 cases were eligible. Inverse variance weighted random-effects models were conducted to pool estimates and stratify associations by infection type and heart defect type.

**Results:**

A total of 30 studies (24 case-control, 3 cohort, and 3 cross-sectional studies) with 1 732 295 pregnancies were identified. Studies assessed maternal infectious status through self-reported questionnaires (*n* = 20, 66.7%), laboratory testing (*n* = 7, 23.3%) or medical records (*n* = 3, 10.0%). Overall, any first-trimester maternal infection was associated with higher risk of CHD in offspring, with a pooled odds ratio (OR) and 95% confidence interval (CI) of 1.63 (1.41, 1.88). Among specific types of infection, rubella virus, coxsackievirus, respiratory infections, and influenza presented higher risks of offspring CHD, with ORs (95% CI) of 2.78 (2.08, 3.72), 1.57 (1.12, 2.19), 1.57 (1.25, 1.96), and 1.50 (1.20, 1.87), respectively. Studies that reported associations by individual subtype of CHD relied on a comparatively modest number of cases. Pooled ORs for exposure to any first-trimester infection were 1.59 (1.16, 2.20) for ventricular septal defects, 1.55 (1.21, 1.99) for atrioventricular septal defects, and not statistically significant for other subtypes.

**Conclusions:**

First-trimester maternal infections are associated with increased risk of offspring CHD and appear to extend beyond infections commonly tested for during routine pregnancy screening. Larger-scale studies are warranted to confirm these findings using laboratory antibody testing and explore underlying mechanisms.

## Introduction

Congenital heart defects (CHD) refer to a range of structural abnormalities of the heart and great vessels that are present from birth.^1^ Although individual defects are relatively rare, collectively, they affect about 1% of births and represent one of the most important causes of infant mortality and morbidity worldwide.^2^

The aetiology of congenital heart anomalies is not fully elucidated and is thought to rely on a combination of multiple interrelated causes—including genetic, nutritional, environmental, lifestyle, socioeconomic, and reproductive factors.^3–5^ Maternal infections during pregnancy are of special interest as they have been shown to be associated with harmful teratogenic effects, including certain congenital anomalies of the heart.^3^

More specifically, a group of pathogens known as TORCH [Toxoplasmosis, Other (syphilis, varicella-zoster, parvovirus B19, and others), Rubella, Cytomegalovirus, and Herpes virus] are recognized for causing congenital anomalies in babies after in-utero exposure.^6^ They are generally thought to act either through inflammation-mediated placental damage, creating fetal hypoxia at crucial times during heart development and therefore cardiac remodelling, or by crossing the placenta and affecting the development of heart tissue, damaging vasculature and/or endothelial cells, during gestation.^6–8^ Yet to date, only rubella during pregnancy has been established to cause CHD; for other TORCH pathogens, previous studies have yielded inconsistent results.^9–18^

A systematic review performed before the COVID-19 pandemic demonstrated an association between certain viral pathogens and risk of overall CHD in offspring.^19^ We aimed to extend this work to include a series of recent large-scale studies,^11–13^ by investigating a broader range of viral as well as bacterial and parasitic infections, and by examining how associations might vary by specific subtypes of CHD.

## Methods

This systematic review and meta-analysis is reported in accordance with the Preferred Reporting Items for Systematic Review and Meta-Analysis guidelines.^20^ The protocol of this systematic review and meta-analysis was registered with the International Prospective Register of Systematic Reviews (PROSPERO) as CRD42024523638.

### Literature search and eligibility criteria

We searched Embase, PubMed, Web of Science, Scopus, and Cochrane Library from inception to 13 February 2024, without language restriction. We used a detailed search strategy covering concepts of pregnancy, infections, and CHD (see Supplementary data online, Table S2). We used EndNote for study de-duplication, following a structured process that involved both automatic detection and manual verification. Two reviewers (H.S. and E.G.) then independently screened the titles and abstracts in the blinded setting of the reference managing site Rayyan,^21^ and then assessed the full text for eligibility. Artificial intelligence enhanced software functionalities were not used. Discrepancies were resolved by discussion between the two reviewers and/or a third reviewer (N.C.). The bibliography of eligible studies and related meta-analyses was hand-searched for relevant studies. An updated search was performed through 30 September 2024, with no additional eligible studies identified. Articles written in languages other than English were translated by native-speaking authors (H.S. for studies in Chinese).

Human studies, with case-control, cohort, or cross-sectional designs, that reported the association between maternal infections during pregnancy and the risk of offspring CHD with at least 50 cases were eligible for inclusion. The threshold of 50 cases was set to minimize the risk of small study bias. We excluded studies that: (i) were reviews, commentaries, case reports, conference abstracts, editorials, or other non-primary research articles; (ii) did not report data of interest (i.e. either estimates or number of infected/non-infected women and number of cases/controls); (iii) were ecological in nature (i.e. studies that did not measure maternal infections at the patient-level); (iv) only reported results for maternal infection at times other than during pregnancy (e.g. at birth or later); (v) used indirect markers for maternal infections (e.g. antimicrobial use or vaccination); or (vi) had not been peer-reviewed. Studies that, to account for the inherent uncertainty in ascertaining the exact date of conception and/or infection, reported on infections during pregnancy as well as up to 3 months pre-conception were considered as eligible for inclusion.

### Data extraction and quality assessment

Data extraction was performed using a standardized data collection form, to collate information on study authors; publication year; study region; study design; study period; study setting; sample size; type of infection or pathogen; timing and measurement method of maternal infection; subtype, timing, and ascertainment method of CHD; matching/adjusting variables; inclusion of pregnancy terminations or not; sample size and number of events in each group, and reported effect size [point estimates and 95% confidence interval (CI)] for the association between infection and CHD. Infection assessment methods were further categorized into ‘self-report,’ ‘medical records,’ or ‘laboratory testing.’ ‘Laboratory testing’ was assigned only when studies explicitly referred to microbiological or serological tests (e.g. PCR, ELISA) to assess infection exposure, whereas ‘medical records’ referred to clinical documentation at the time of infection without explicit reference of laboratory confirmation. When multiple studies from the same population were identified on the same exposure and outcome variables, only the one with the largest sample size or the latest one was included;^22,23^ however, if the study samples were independent (e.g. participants from the same hospital but enrolled in a different year),^24,25^ they were treated as separate studies. When multiple studies from the same population reported different exposures (i.e. different types of maternal infection),^26–29^ data were extracted separately, but only the largest sample size was included in the total sample calculation.

Risk of bias was assessed independently by two investigators (H.S. and E.G.) using the Newcastle-Ottawa scale.^30^ This scale assesses each study on the selection of the study groups; the comparability of the groups; and the ascertainment of the exposure and/or outcomes of interest. Quality scores of 0–3, 4–6, and 7–9 are regarded as high, moderate, and low risk of bias, respectively. In addition, we performed a supplementary risk-of-bias assessment using the more recently developed Risk Of Bias In Non-randomized Studies—of Exposures (ROBINS-E) tool,^31^ and report these in the Supplement (see Supplementary data online, Figure S6).

### Statistical analysis

Our main analyses present pooled estimates from studies reporting maternal infections occurring during the first-trimester of pregnancy (considering up to 3 months before conception), which were assessed as having low or moderate risk of bias. Restriction of main analyses to studies reporting maternal infections occurring during the first-trimester of pregnancy was set because human organogenesis occurs during the first few weeks of pregnancy,^32^ and the consideration of up to 3 months before conception was set due to the inherent uncertainty in ascertaining the exact date of conception and/or infection.

To investigate how maternal infections in general may contribute to the aetiology of CHD, for example, through immunological responses, we performed pooled analyses considering all types of infections. To avoid multiple counting, each study was only included once in any meta-analytic pooling. If a study reported effect estimates for more than one infection variable, we considered the infection variable with the highest prevalence as a proxy for any infection in that study. We further quantified associations by specific type of infection and CHD when data from at least two studies was available. Studies reporting multiple infection types were included in each corresponding infection-specific meta-analytic pooling.

When studies reported multiple types of immunoglobulins, we reported associations based on IgM measures, as these would indicate the occurrence of a recent infection. If studies categorized CHD into groups, such as selected and major CHD, the analysis for overall CHD included data from the group covering most subtypes of CHD. For studies reporting CHD with and without extracardiac defects, the analysis focused on the group without extracardiac defects. Where studies reported both crude and adjusted estimates, we used estimates that were adjusted for potential confounders.

If the studies did not report effect estimates but provided raw data of cell counts, we constructed 2×2 tables and calculated the crude odds ratios (OR). We added 0.5 to all cells before OR calculation when there was a null value in 1 of the 4 cells. Given the rareness of the studied outcome, we considered different risk estimates (e.g. relative risks) as equivalent to OR.

To synthesize estimates across studies, we calculated pooled OR and their corresponding 95% CI using inverse variance weighted random-effects model, using the DerSimonian and Laird method.^33^ Heterogeneity of effect size across studies was tested by using the *Q* statistics at the *P* < .10 level of significance and the *I*^2^ statistic at the significance level at *I*^2^ > 50%.^34^

Subgroup analyses were performed by study region (Asia, Europe, North America, or others), study design (case-control, cohort, or cross-sectional study), study setting (population or hospital), infection assessment method (self-report, medical records, or laboratory testing), control for confounding (adjusted/matched for maternal age, adjusted/matched for other covariates excluding maternal age, no adjustment/matching for any covariates), inclusion of pregnancy terminations, and risk of bias (low or moderate). Additionally, we conducted *post hoc* subgroup analyses based on the timing of CHD diagnosis (prenatal or at birth, within the first year, after the first year, and unspecified), and the presence or absence of extracardiac defects, with the two groups defined as mutually exclusive subgroups within the same study population. We assessed subgroup differences using the metagen function from the meta package in R, which implements a Cochran’s *Q* test for heterogeneity between subgroups.

Sensitivity analyses were performed by: (i) excluding studies that did not adjust for or match on any covariates; (ii) excluding studies that investigated maternal infections occurring more than 1 month before conception (leaving studies examining infections from 1 month before conception up to the end of the first-trimester of pregnancy); (iii) excluding studies that investigated maternal infections occurring before conception (leaving studies examining infections in the first-trimester or early pregnancy); (iv) including studies that investigated infection exposure after the first-trimester or at an unspecified time-point during pregnancy; (v) including studies that were rated as having a high risk of bias; (vi) *post hoc*: excluding studies that reported cyanotic CHD rather than overall CHD; (vii) *post hoc*: including studies with fewer than 50 CHD cases (see Supplementary data online *Text S1* and Table S1 for details); (viii) *post hoc*: only including studies rated as low or moderate risk of bias according to the ROBINS-E tool.

To investigate how individual studies affect overall pooled estimates, we performed leave-one-out meta-analysis. Publication bias was assessed visually through funnel plot and quantitatively via Egger’s test (*P* > .10 indicates no publication bias). We further used the ‘Trim and Fill’ method to simulate missing studies, plotted imputed studies alongside observed ones in funnel plots, and re-evaluated risk estimates to examine how pooled estimates would change in a publication-bias-free scenario.

Subgroup analyses, sensitivity analyses, and publication bias analyses were performed for the overall association between any first-trimester maternal infections and any CHD because of the small number of studies for specific types of infections and CHD subtype.

All reported *P*-values are two-sided and *P* < .5 was considered statistically significant, except where otherwise specified. All analyses were performed using R software (version 4.3.2, R Core Team, R Foundation for Statistical Computing, Vienna, Austria).

## Results

### Literature search

**Figure 1:**
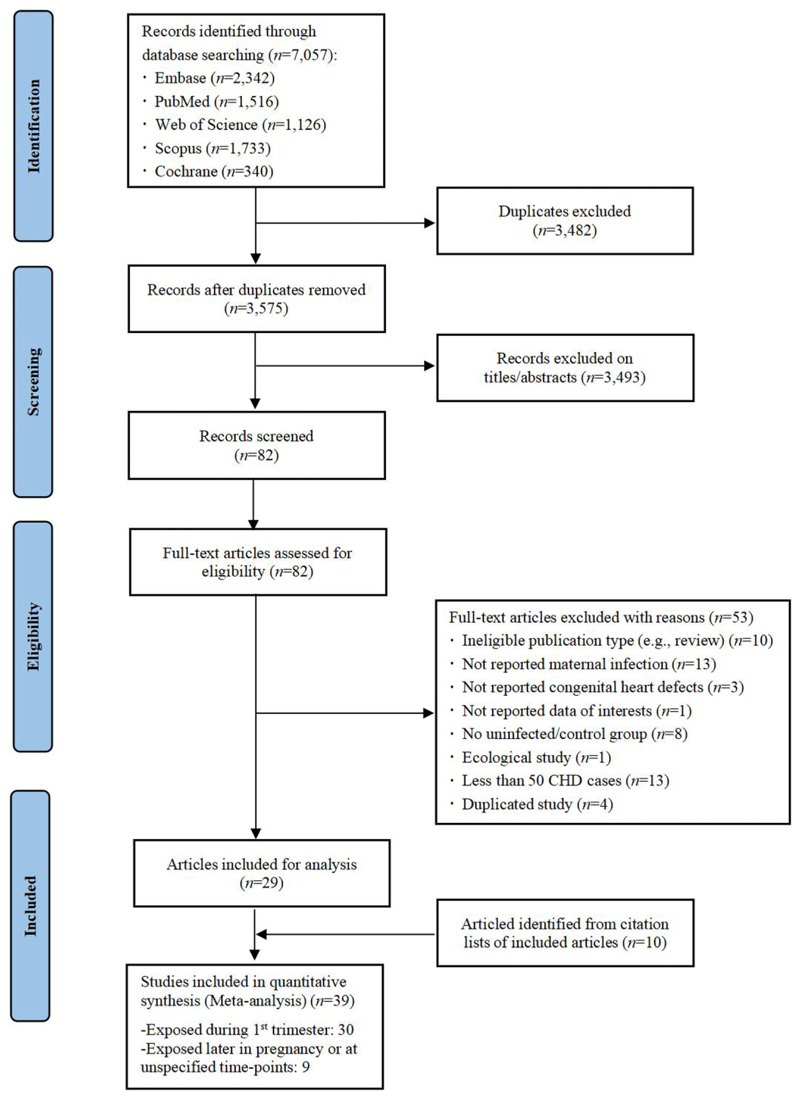
Study selection flow chart

Of 7057 records identified from Embase, PubMed, Web of Science, Scopus, and Cochrane Library, 82 were eligible for full-text assessment. After further excluding 53 articles based on study eligibility criteria, the remaining 29 studies together with 10 studies identified through citation searching (including four studies published in Chinese, and six studies using keywords or terminologies that we did not search on), were included in analyses (Figure 1).

### Characteristics of included studies

A total of 39 studies published from 1972 to 2023, and involving 49 495 104 participants were eligible.^11–18,22–29,35–57^ A total of 30 (76.9%) studies investigated infections during the first-trimester and were used in the main analyses. The remaining studies investigated infections at unspecified time during pregnancy (*n* = 8, 20.5%) or did not report the exposure time (*n* = 1, 2.6%), and were used in sensitivity analyses (Table 1, Supplementary data online, Tables S3 and S4).

Among the 30 included studies, 24 (80.0%) studies had a case-control design, while 3 (10.0%) studies had a cohort design, and 3 (10.0%) studies had a cross-sectional design. Fifteen (50.0%) studies were conducted in Asia (13 studies in China), 7 (23.3%) studies in North America, 7 (23.3%) studies in Europe, and 1 (3.3%) study in Oceania. Twenty-six (86.7%) studies were written in English and four (13.3%) were written in Chinese. A total of 16 (53.3%) studies were population-based and the remaining 14 (46.7%) studies relied on hospitals to identify participants (Table 1, Supplementary data online, Table S4).

The included 30 studies investigated 31 different types of infection variables: 16 were viral or virus-related infections (e.g. cytomegalovirus), 5 were bacterial or parasitic pathogens (i.e. chlamydia, gonorrhoea, toxoplasma, syphilis, trichomoniasis), and 10 were general infections that did not specify the exact pathogen (e.g. respiratory infection). We found no study that investigated the association between COVID-19 during the first-trimester of pregnancy and CHD and that met the inclusion criteria for a minimum number of 50 CHD cases. A total of 28 studies reported associations with overall CHD, 12 reported individual CHD subtypes, and 10 reported associations both overall and by CHD subtype (Table 1, Supplementary data online, Table S4). Out of a maximum of 9, the Newcastle-Ottawa scale scores ranged from 1 to 8: 16 studies had a low risk of bias (score of ≥7), 13 moderate risk of bias (score of 4–6), and 1 high risk of bias (score of ≤3). The most common sources of bias were the lack of reporting on non-response rates (24 out of 27 case-control or cross-sectional studies), lack of control for key confounders, or any confounders at all (15 out of 30 studies), and the lack of robust methods for ascertainment of exposure (13 out of 30 studies) (see Supplementary data online, Table S5).

### Any first-trimester maternal infection and overall CHD in offspring

**Figure 2:**
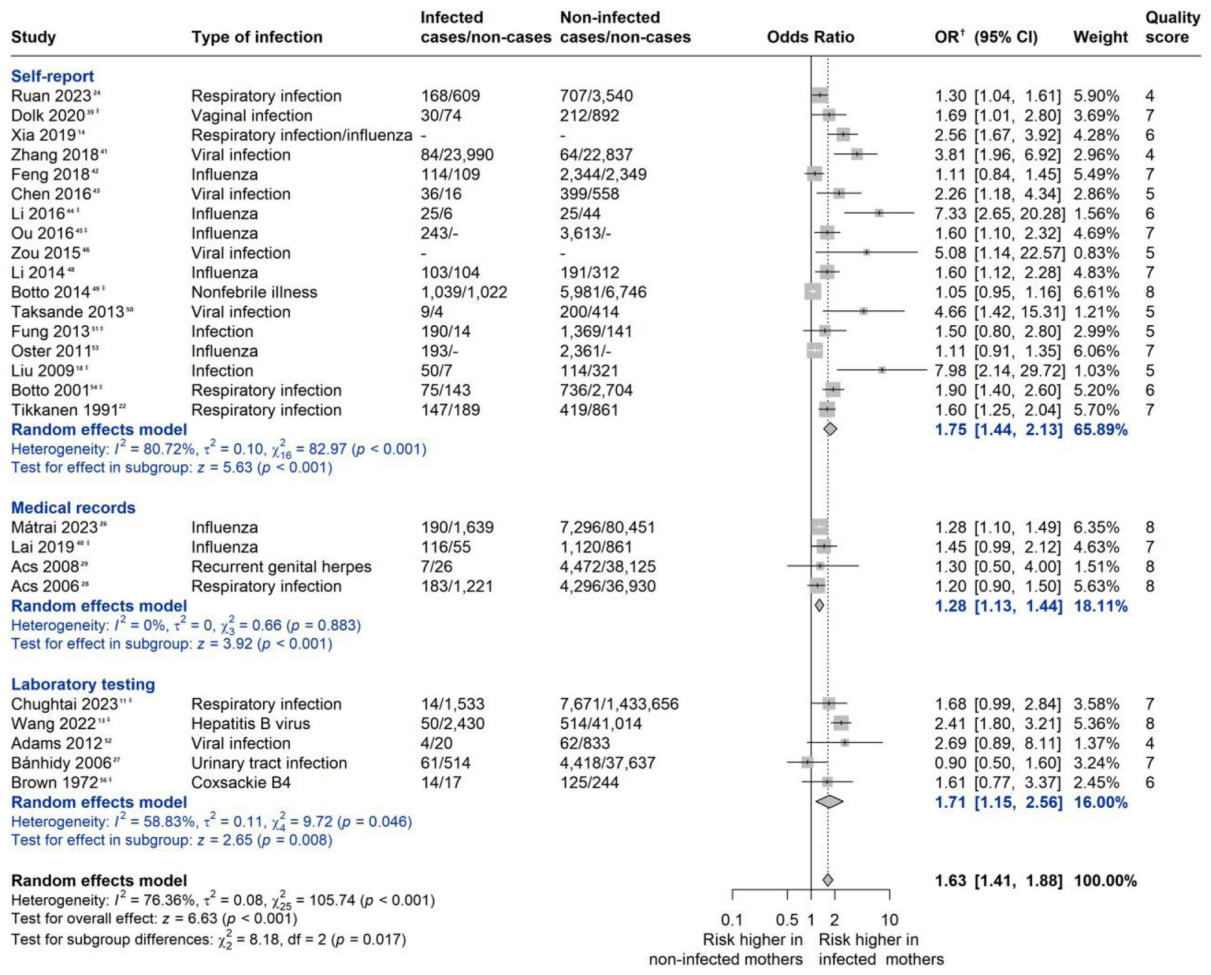
Meta-analysis of any first-trimester maternal infection for overall congenital heart defects in offspring. Forest plot presenting the association between any maternal infection during the first-trimester of pregnancy and risk of overall congenital heart defects in the offspring. Similarly, studies that reported several groups of congenital heart defects were included using data from the group covering largest number of congenital heart defects or the group without extracardiac defects. Pooled OR and their corresponding 95% CI were calculated using inverse variance weighted random-effects model. The quality score was calculated using the Newcastle-Ottawa Scale. Scores of 0–3, 4–6, and 7–9 are regarded as high, moderate, and low risk of bias, respectively. Main analyses were restricted to the 26 studies with low or moderate risk of bias. OR, odds ratio. CI, confidence interval. ^†^: Wang 2022 reports relative risk, others report odds ratio. ^‡^: In studies reporting associations for more than one infection, the infection variable with the highest prevalence (‘Type of infection’ column) was used as the quantitative summary for any infection in pooled analyses

Among 26 low-or-moderate-risk-bias studies that investigated infections during the first-trimester and reported associations with overall CHD, effect estimates ranged from 0.90 to 7.98. Among these studies, 25 (96.15%) reported point estimates above 1, of which 15 were statistically significant. Meta-analytic pooling of those risk estimates yielded a pooled OR (95% CI) of 1.63 (1.41, 1.88), with substantial heterogeneity (*P* < .001, *I*^2^ = 76.36%) (Figure 2). Estimates from studies relying on self-report to assess infection exposure [pooled OR (95% CI): 1.75 (1.44, 2.13); *I*^2^ = 80.72%] were similar to those relying on laboratory testing [pooled OR (95% CI): 1.71 (1.15, 2.56); *I*^2^ = 58.83%], yet higher than those relying on medical records [pooled OR (95% CI): 1.28 (1.13, 1.44); *I*^2^ = 0%] (Figure 2).

The funnel plot was asymmetrical with nine potentially missing studies (see Supplementary data online, Figure S1A), indicating possible publication bias (Egger’s test: *P* < .001). The re-evaluated OR (95% CI) based on the ‘Trim and Fill’ method was 1.32 (1.12, 1.57) (see Supplementary data online, Figure S1B).

### Specific type of first-trimester maternal infection and subtypes of CHD in offspring

**Figure 3:**
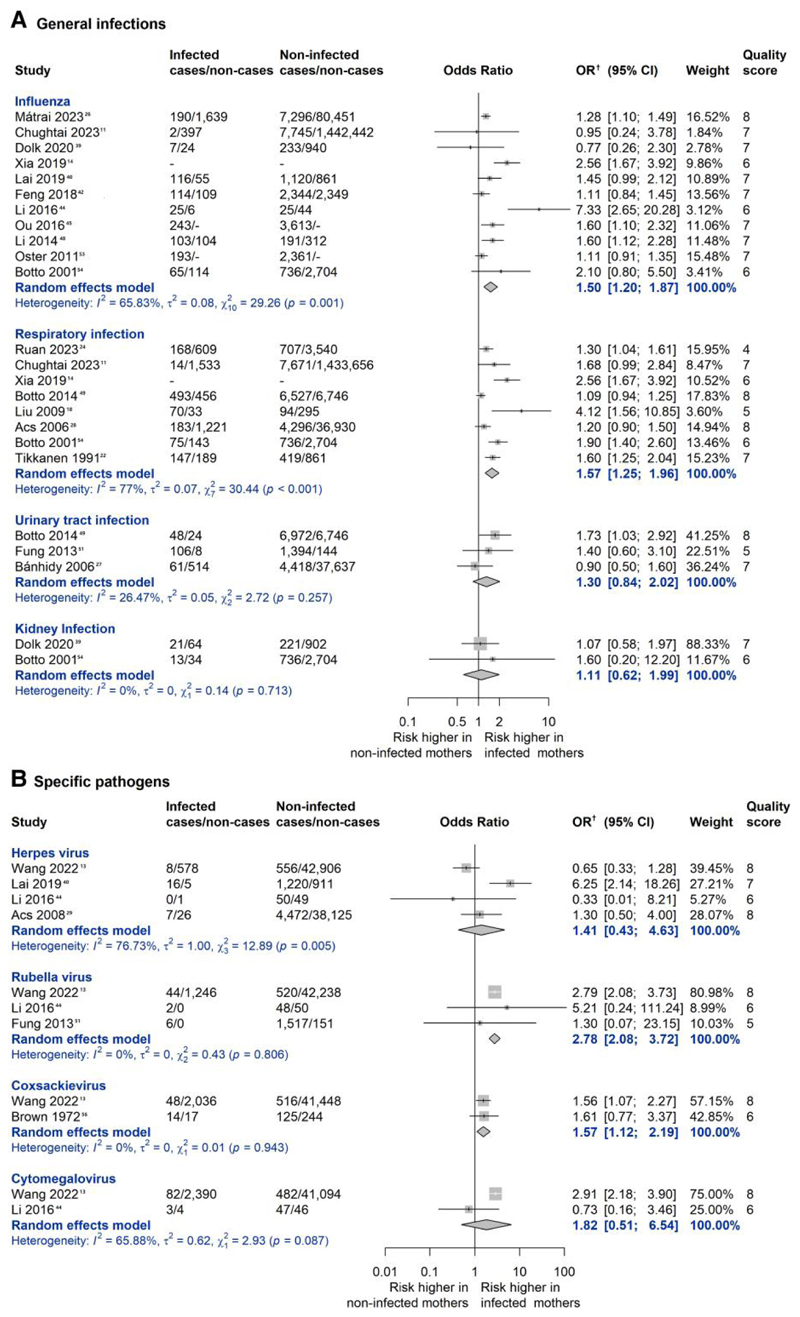
Meta-analysis of any first-trimester maternal infection for overall congenital heart defects in offspring, by specific type of infection. Forest plot presenting the association between specific type of maternal infection during the first-trimester of pregnancy and risk of overall congenital heart defects in the offspring. General infections refer to those that did not specify the exact pathogen, such as respiratory infection and urinary tract infection. Studies that reported several groups of congenital heart defects were included using data from the group covering largest number of congenital heart defects or the group without extracardiac defects. Pooled OR and their corresponding 95% CI were calculated using inverse variance weighted random-effects model. The quality score was calculated using the Newcastle-Ottawa Scale. Scores of 0–3, 4–6, and 7–9 are regarded as high, moderate, and low risk of bias, respectively. Main analyses were restricted to the 26 studies with low or moderate risk of bias. OR, odds ratio. CI, confidence interval. ^†^: Wang 2022 reports relative risk, others report odds ratio

Data from at least two studies were available for four general infections and four specific pathogens: influenza (*n* = 11), respiratory infection (*n* = 8), urinary tract infection (*n* = 3), kidney infection (*n* = 2), herpes virus (*n* = 4), rubella virus (*n* = 3), coxsackievirus (*n* = 2), cytomegalovirus (*n* = 2). Among them, influenza [pooled OR (95% CI): 1.50 (1.20, 1.87); *I*^2^ = 65.83%], respiratory infection [pooled OR (95% CI): 1.57 (1.25, 1.96); *I*^2^ = 77.00%], rubella virus [pooled OR (95% CI): 2.78 (2.08, 3.72); *I*^2^ = 0%] and coxsackievirus [pooled OR (95% CI): 1.57 (1.12, 2.19); *I*^2^ = 0%] were associated with higher risk of overall CHD (Figure 3).

**Figure 4:**
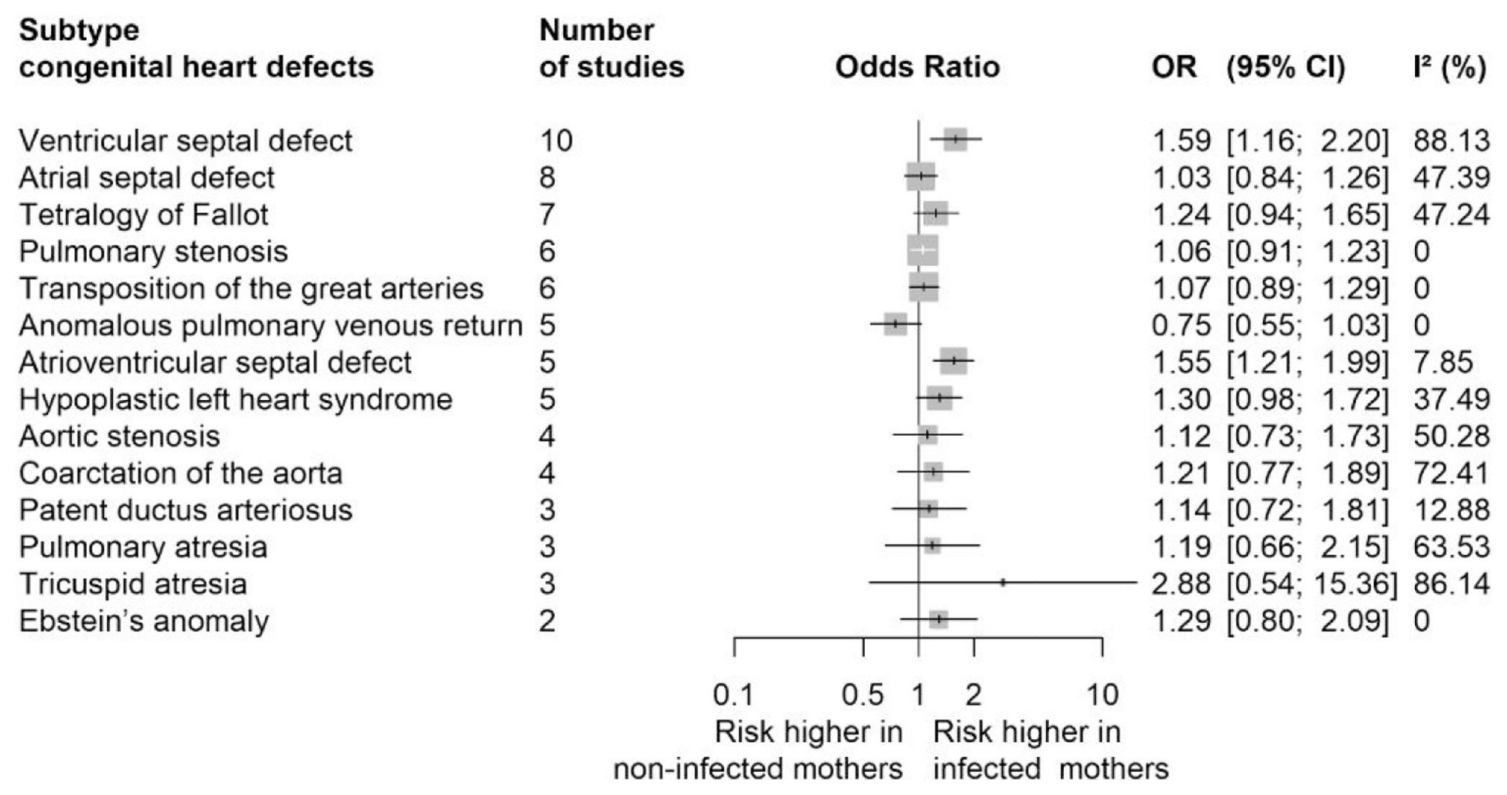
Meta-analysis of any first-trimester maternal infection for congenital heart defects in offspring, by specific type of heart defects. Forest plot presenting the association between any maternal infection during the first-trimester of pregnancy and risk of specific type of congenital heart defects in the offspring. Pooled OR and their corresponding 95% CI were calculated using inverse variance weighted random-effects model

A total of 12 (40.0%) studies reported on 14 CHD subtypes: ventricular septal defect (VSD, *n* = 10), atrial septal defect (*n* = 8), tetralogy of Fallot (*n* = 7), pulmonary stenosis (*n* = 6), transposition of the great arteries (*n* = 6), anomalous pulmonary venous return (*n* = 5), atrioven-tricular septal defect (AVSD, *n* = 5), hypoplastic left heart syndrome (HLHS) (*n* = 5), aortic stenosis (*n* = 4), coarctation of the aorta (*n* = 4), patent ductus arteriosus (*n* = 3), pulmonary atresia (*n* = 3), tricuspid atresia (TA) (*n* = 3), Ebstein’s anomaly (*n* = 2). Among them, any first-trimester maternal infection was associated with a higher risk of VSD [pooled OR (95% CI): 1.59 (1.16, 2.20); *I*^2^ = 88.13%] and AVSD [pooled OR (95% CI): 1.55 (1.21, 1.99); *I*^2^ = 7.85%] (Figure 4, Supplementary data online, Figure S2).

### Subgroup analyses

The association between any maternal infection and overall offspring CHD was significant in all subgroups, yet we observed important variability across subgroups. Pooled ORs (95% CI) ranged from 1.25 (1.12, 1.41) to 2.46 (1.80, 3.37) across subgroups of study region, study design, study setting, infection assessment method, control for confounding, inclusion of pregnancy terminations, risk of bias, and diagnosis timing. Heterogeneity, described as *I*^2^, was particularly high (>90%) for inclusion/exclusion of pregnancy terminations and risk of bias groups, while the *I*^2^ for the *post hoc* subgroup analysis by follow-up time was 0%, indicating no significant heterogeneity within this sub-group (Table 2, Supplementary data online, Figure S3). Originally planned analyses per subgroups of maternal age and fetal sex were not performed as less than two studies reported on these subgroups. Four studies reported CHD both without, and with, extracardiac defects, yielding a pooled OR (95% CI) of 1.24 (1.00, 1.54) without and of 1.22 (1.05, 1.42) with extracardiac defects. A Cochran’s *Q* test for subgroup differences indicated no significant difference between the two groups (*P* = .913) (see Supplementary data online, Figure S4).

### Sensitivity analyses

Sensitivity analyses of any maternal infection and overall CHD in off-spring were conducted to explore the potential sources of heterogeneity and to examine the robustness of the results (see Supplementary data online, Table S6). Exclusion of a study that neither adjusted for nor matched on any covariate showed a modestly increased risk estimate compared with main analyses [pooled OR (95% CI): 1.64 (1.40, 1.91); *I*^2^ = 76.95%]. Exclusion of studies investigating maternal infections occurring either more than 1 month or any time before conception showed a slightly increased risk estimate compared with main analyses [pooled OR (95% CI): 1.69 (1.43, 1.99); *I*^2^ = 77.37% and 1.62 (1.38, 1.91); *I*^2^ = 77.07%, respectively]. When including studies with a high risk of bias or exposure at times other than during the first-trimester of pregnancy, risk estimates remained similar but the heterogeneity was larger. Three *post hoc* sensitivity analyses were performed. First, excluding three studies that analysed cyanotic CHD rather than overall CHD showed a slightly increased risk estimate compared with the main analyses [pooled OR (95% CI): 1.71 (1.32, 2.23); *I*^2^ = 98.03%], but with increased heterogeneity. Second, including the only three studies with fewer than 50 CHD cases, which were initially excluded solely based on our pre-specified sample size threshold, despite meeting all other eligibility criteria, resulted in a consistent estimate (OR: 1.66; 95% CI: 1.43, 1.91; *I*^2^ = 75.49%). Third, only including studies rated as low or moderate risk of bias according to the ROBINS-E tool, which also produced a similar result (OR: 1.64; 95% CI: 1.40, 1.91; *I*^2^ = 76.95%). Leave-one-out analyses did not substantially affect the overall risk estimates. Excluding the one study (Wang *et al*.)^13^ reporting relative risk rather than OR, resulted in a pooled OR (95% CI) of 1.58 (1.37, 1.81), consistent with main analysis (see Supplementary data online, Figure S5).

## Discussion

In this systematic review and meta-analysis, maternal infections during the first-trimester of pregnancy were associated with an increased risk of offspring CHD. Associations were consistent across infection assessment methods, including infections recorded in electronic health records at the time of exposure and those validated through laboratory tests. However, heterogeneity was high and pooled estimates often relied on small number of studies and cases, especially for specific infections or/and heart defect subtypes, so that caution is indicated in the interpretation of these findings.

Findings from this study are consistent with a previous review investigating viral infections and risk of overall CHD in the offspring,^19^ and with the established association between maternal rubella and fetal heart anomalies.^58,59^ Our results expand previous knowledge by identifying additional pathogens not typically part of routine pregnancy screening, particularly coxsackievirus, that may also pose risks to fetal heart development. In our study, while several viral infections showed significant associations with the risk of CHD, pooled estimates for infections typically caused by bacteria (e.g. urinary tract or kidney infections) did not present significant associations. Together with the low heterogeneity observed among specific pathogens, such as rubella virus and coxsackievirus, these findings point towards organism-specific mechanisms. In contrast, the important heterogeneity among infections characterised by clinical presentation (e.g. respiratory infection) could be driven by a single (or few) potentially rare pathogen(s) yet to be identified.

By taking advantage of recent large-scale studies, our review also delved into individual CHD subtypes and found that first-trimester infections were associated with a 59% and 55% higher risk of VSD and AVSD, respectively. Estimates of association were also elevated for several other specific defects, including for example, HLHS or TA, yet without reaching statistical significance. Whether observed associations with septal defects are due to biological susceptibility or the higher prevalence of septal defects, which may make it easier to detect the associations with the currently available sample size, requires further investigation.

Reliable infection assessment methods are crucial to establish exposure, its timing, and the specific pathogen involved. In this review, the number of studies that assessed infection status using laboratory tests and by individual pathogen was modest and limited the granularity of our analyses. The effort needed to measure maternal exposures using biospecimens in large population-based cohorts presents a major challenge to address this gap. Large-scale prospectively collected mother and child biobanks and international consortia collaborations with pooled cohort data provide valuable resources to address the question of perinatal exposures and offspring health outcomes.^60,61^ Initiatives and regulations making these cohorts more broadly accessible will hopefully provide impetus for more studies to investigate the aetiology of CHD, and more generally of intergenerational health outcomes.

Several biological processes have been proposed to explain how maternal infections may be associated with offspring CHD. One possible mechanism relies on maternal immunological and inflammatory responses to infection at the time of conception. These processes, along with the subsequent change in cytokines expression can result in cell death and gene expression alterations, potentially impairing embryonic development.^62,63^ Other hypotheses that have been formulated involve mediation through maternal fever and/or associated therapeutic drugs (e.g. antipyretics), infection-related placental dysfunction resulting in reduced absorption of oxygen and nutrients, or placental proinflammatory cytokines impairing critical functions in the developing placenta and fetus.^64–66^ Findings from our study suggest that mechanisms by which maternal infections may play a role in the development of congenital heart anomalies are not generalized across infection types and more likely to be pathogen-specific or relate to distinct inflammatory pathways. One such mechanism could involve the vertical transmission of pathogens across the placental barrier, directly causing fetal infection and disrupting organogenesis.^6^ Evidence from previous studies indicates that certain viruses, such as the rubella virus, act as human teratogens, producing prolonged fetal exposure to toxic metabolites, inducing oxidative stress, and dysregulating the normal embryological process of cardiac morphogenesis,^67–69^ particularly when the infection occurs early in pregnancy. Further research is needed to fully understand these mechanisms and to explore potential interventions.

Another important consideration is how other maternal health attributes might confound the observed associations. Studies included in this meta-analysis commonly accounted for maternal age, race/ethnicity, education, smoking, alcohol, parity, family history of CHD, BMI, and gestational diabetes or hypertension, but many factors likely remain unaccounted for. One such example could be maternal immunemediated or inflammatory conditions, which might increase a mother’s susceptibility to infections and cause CHD through auto-antibody or inflammatory-mediated mechanisms.^70,71^ Associations between systemic lupus erythematosus and congenital heart block are well-established,^72^ yet there are over 100 different maternal immunemediated inflammatory conditions affecting about 10% of the population that have not been studied to the same extent.^7,73^ How socioeconomic factors and/or genetic predispositions may influence both infection exposure or severity and risks of CHD through different mechanisms also requires further research.

A key strength of this study is the pooling data from 1 732 295 patients enrolled in 30 studies, hence including substantially more information than previous meta-analyses that have addressed this question. This allowed us to investigate the association between maternal infection and offspring CHD by specific types of infections and sub-types of heart defects, providing comprehensive analyses and new insights for future research. If confirmed in larger-scale studies, associations between maternal infections and increased risk of CHD would have important clinical implications. Immediate public health interventions could include targeted screening in high risk groups and infection prevention measures, e.g. through masking, hand-washing or addressing possible sources of occupational exposures in pregnant women. Longer-term and more comprehensive strategies would involve trials to investigate the optimal management in affected mothers as well as intensified funding and research in vaccine development for those infections currently lacking effective vaccines.

Several limitations should be taken into account when interpreting these findings. First, substantial heterogeneity was observed among studies investigating the association between maternal infection and overall CHD, which was partly due to the wide variations in study settings (e.g. study region and study design) and methodology (e.g. types of infections and assessment method). Risk estimates remained significant across subgroups and sensitivity analyses, but interpretation should be cautious due to the heterogeneity. Second, the asymmetry observed in the funnel plot suggests potential publication bias, as supported by Egger’s test (*P* < .001), and the more modest pooled estimates derived from the ‘Trim and Fill’ method. While publication bias may have led to an overestimation of the true effect size, *post hoc* sensitivity analyses including studies with fewer than 50 CHD cases showed comparable results (OR: 1.66; 95% CI: 1.43, 1.91). Third, for the meta-analyses of specific infections or/and subtypes of heart defects, results mainly relied on a modest number of studies, which limited statistical power. More studies are warranted to investigate associations by specific pathogens and subtypes of CHD. Fourth, infection exposure assessment methods varied widely across studies. Studies relying on self-reported infections may be prone to recall bias and <25% of studies in our review relied on laboratory-confirmed infections. To ensure more reliable and comparable results, future studies should prioritize using standardized and accurate infection assessment methods, such as laboratory tests. Fifth, variability in follow-up times across the included studies might lead to inconsistencies in detection and reporting. The cross-sectional studies that diagnosed CHD prenatally or during school-age screening may suffer from misclassification or survival bias and differ from birth-based diagnoses in terms of severity. However, our *post hoc* subgroup analysis showed no significant heterogeneity (*I*^2^ = 0%), suggesting diagnosis timing did not substantially affect findings. Future research, especially large prospective cohort studies with longer follow-up durations, is warranted to capture late-diagnosed CHD cases. Sixth, when estimating the total population, we calculated the maximum sample size within the same cohort (e.g. for the Hungarian Case-Control Surveillance of Congenital Abnormalities, only 1980–2009 was included,^26^ not 1980–1996^27–29^). However, overlaps between study sources (e.g. three US studies using the National Center for Health Statistics natality database,^12^ 2012 US Birth Certificates,^17^ and Centres for Disease Control and Prevention 2017 Natality public use file^38^) might inflate the total number of individuals appear in multiple datasets.

In conclusion, we found that first-trimester maternal infections were associated with increased risk of offspring CHD in this systematic review. Associations appear to vary by type of infection and type of heart defects, and to extend beyond infections commonly tested for during routine pregnancy screening. Larger-scale studies are warranted to confirm these findings using laboratory antibody testing and explore underlying mechanisms.

## Supplementary Material

Supplementary data are available at European Heart Journal online.

Supplementary data

## Figures and Tables

**Table 1:** Characteristic of 30 studies of first trimester maternal infections and risk of congenital heart defects in offspring

Study	Study region	Study design	Study period	Study setting	Sample size	Investigated exposures	Exposure timing^b^	Reported outcomes	Matching/adjusting variables
Ruan *et al.*^24^	China (Asia)	Cross-sectional	May 2018– September 2019	Population-based	875 CHD cases and 4 149 non-CHD cases	Upper respiratory tract infection	Early pregnancy	CHD	Adjusted for spontaneous abortion, mental stress during early pregnancy, paternal smoking, and fetal single umbilical artery.
Mátrai *et al.*^26^	Hungary (Europe)	Case-control	1980–2009	Population-based	7486 CHD cases and 82,090 controls	Influenza	1st trimester	CHD; VSD; ASD; PDA; congenital malformations of heart, unspecified; other congenital malformations of cardiac chambers and connections	Matching variables: sex, birth week, and district of residence of the parents. Adjusted for maternal age, birth order (parity), and job position.
Chughtai *et al*.^11^	Australia (Oceania)	Cohort study	2001–2016	Population-based	1 453 037 birth records	Acute respiratory infection (0.90%);^a^ influenza (0.50%); high-risk infection (0.78%)	1st trimester; 2nd-3rd trimesters	Selected cardiovascular anomalies; major cardiovascular anomalies	Adjusted for maternal age group at delivery, smoking during pregnancy, remoteness of area of residence, quartile of socioeconomic status based on area of residence, previous pregnancy, country of birth, hospital of delivery, the number of weeks pregnant at first antenatal visit, Indigenous status, hypertension, and diabetes.
Wang *et al.*^13^	China (Asia)	Cohort study	March 13, 2013– December 31, 2019	Hospital based	564 CHD cases and 43 484 non-CHD cases	Hepatitis B virus (5.63%);^a ^coxsackievirus-B (4.73%); human cytomegalovirus (5.61%); herpes simplex virus (1.33%); rubella virus (2.93%)	Early pregnancy	CHD; ASD; VSD; AVSD; PDA; TOF; PS; TGA	Adjusted for educational level, age, ethnicity, history of adverse pregnancy outcomes, pre-pregnancy BMI, prepregnancy diabetes mellitus, and other 4 type virus infections.
Ruan *et al.*^25^	China (Asia)	Cross-sectional	June 2010– June 2017	Population-based	3312 CHD cases and 12 774 non-CHD cases	Upper respiratory infection	Early pregnancy	CHD	NA
Dolk *et al*.^39^	Ireland (Europe)	Case-control	September 2014– February 2017	Population-based	242 CHD cases and 966 controls	Vaginal infection (8.61 %);^a^ kidney Infection (7.04%); influenza (2.57%)	1st trimester	CHD	Adjusted for maternal age, previous pregnancy, maternal education, socioeconomic deprivation of area of residence, dietary class, BMI category, folic acid supplementation, smoking, antidepressant prescription in first trimester, pregnancy stress, and multiple stressors.
Xia *et al*.^14^	China (Asia)	Case-control	June 2016– December 2017	Hospital based	524 CHD cases and 262 controls	Upper respiratory tract infection/ influenza	1st trimester	All CHD; simple CHD; complex CHD	Adjusted for maternal ethic, maternal age at delivery, maternal education, marital status, residence, maternal prepregnancy obesity, multiple births, infant gender, family history of CHD, prepregnancy diabetes/hypertension, folic acid use, and smoking/drinking.
Lai *et al.*^40^	China (Asia)	Case-control	February 2010– December 2014	Hospital based	1 236 CHD cases and 916 controls	Influenza (7.77%);^a^ herpes (0.98%)	Periconceptional period	All CHD; isolated cardiac defect; complex malformations	Matching variables: same hospital, and same period. Adjusted for maternal age, maternal education level, residence, parental smoking, BMI, folic acid supplementation, and family history of a CHD.
Zhang *et al.*^41^	China (Asia)	Cross-sectional	January 2015– January 2017	Population-based	148 CHD cases and 46 827 non-CHD cases	Viral infection	Early pregnancy	CHD	Adjusted for family history of CHD, history of exposure to toxic substances, and chemical exposure in early pregnancy.
Howley *et al*.^15^	USA (North America)	Case-control	October 1997- December 2011	Population-based	40 861 birth records	Genitourinary infection (8.18%);^a ^urinary tract infection (7.35%); sexually transmitted infection (1.41%) (Chlamydia, bacterial vaginosis, human papillomavirus, herpes virus, trichomoniasis, gonorrhea)	Periconceptional period	Truncus arteriosus; TOF; D-TGA; DORV-TGA; other DORV; conoventricular VSD; AVSD; total APVR; HLHS; CoA; AS; PA; PS; TA; EA; perimembranous VSD; muscular VSD; secundum ASD; single ventricle defects; heterotaxy.	Matching variables: same time period, and same geographic area. For defects groups with 5+ exposed cases, adjusted for maternal age (continuous), race/ethnicity, education, BMI, smoking, folic acid supplement use, and state of residence at the time of birth.
Feng *et al.*^42^	China (Asia)	Case-control	November 2011– December 2017	Hospital based	2458 CHD cases and 2 458 controls	Influenza infection	1st trimester; during pregnancy	CHD; VSD; ASD; PDA; PS; TOF; VSD & ASD; multiple defects	Matching variables: child’s gender and birth date, and parent’s prefecture of residence. Adjusted for maternal age, parity, gravidity, education status, maternal diseases, medicine consumptions, and pregnancy supplementations.
Chen *et al.*^43^	China (Asia)	Case-control	May 1,2012– October 1, 2013	Hospital based	435 CHD cases and 574 controls	Viral infection	Periconceptional period	CHD	Matching variables: same period, and same hospital. Adjusted for medicine application during the first trimester of pregnancy, home decoration, hair perming and dying, and parents work environment exposure during peri-conceptional period.
Li *et al*.^44^	China (Asia)	Case-control	January 2014– December 2014	Hospital based	50 CHD cases and 50 controls	(1) Parvovirus (6%); toxoplasma (3%); rubella virus (2%); herpes virus (1%); cytomegalovirus (7%) (2) Flu (48%);^a^ reproductive tract infection (14%)	(1) 1st trimester; (2) 1st trimester; 2nd-3rd trimester; during pregnancy	CHD	Matching variables: same period, and same hospital.
Ou *et al*.^45^	China (Asia)	Case-control	January 1, 2004– December 31, 2013	Population-based	4034 CHD cases and 4 034 controls	Viral infection (5.14%); syphilis (0.34%); influenza (6.30%);^a^ infection of rubella (0.16%)	1st trimester	Isolated CHD; multiple defects; VSD; ASD; PS; TGA; TOF	Matching variables: same hospital, infant sex, time of conception, and parents’ residence. Adjusted for maternal age, household income, maternal education, family history of birth defect, previous pregnancies with still birth, chemical contact, passive smoking, paternal smoking, living in newly renovated rooms, residential proximity to main traffic <50meters, maternal occupation, maternal perinatal diseases and medication use at 1st trimester, and paternal alcohol intake before pregnancy.
Zou *et al.*^46^	China (Asia)	Case-control	January 2008–December 2013	Hospital based	150 CHD cases and 150controls	Virus infection	Early pregnancy	CHD	Matching variables: same hospital, same infant sex, and conception time within 3 months. Adjusted for gestational age, meat intake, oral vitamins, stress during early pregnancy, weight gain during pregnancy, birth weights, partial eclipse during pregnancy, drinking tea, having a fever during early pregnancy, and BMI of mothers before pregnancy.
Li *et al.*^48^	China (Asia)	Case-control	February 2010– October 2011	Hospital based	294 CHD cases and 416 controls	Influenza	1st trimester	All CHD; septal defects; conotruncal defects; left-sided obstructive malformation; right-sided obstructive malformation; APVR; other structural cardiac defects.	Matching variables: maternal gestational age within 2 weeks, and same hospital. Adjusted for maternal age, maternal education, maternal BMI, place of residence, parental smoking, folic acid supplementation, and history of pregnancy with any defect
Botto *et al.*^49^	USA (North America)	Case-control	1997–2005	Population-based	7020 CHD cases and 6 746 controls	Febrile illnesses (7.79%); febrile urinary tract infection or pelvic inflammatory disease (0.52%); febrile respiratory infection (6.89%); nonfebrile illness (14.97%)^a^	1st trimester	All CHD; heterotaxy; TOF; d-TGA; AVSD; total APVR; HLHS; CoA; AS; PA; PS; VSD, perimembranous; VSD, muscular; ASD, secundum; ASD, not otherwise specified	Matching variables: same time period, and same geographic area. Adjusted for maternal age, maternal race/ethnicity, maternal cigarette smoking during the first trimester, maternal alcohol consumption during the first trimester, maternal education, prepregnancy BMI, history of seizures, time to interview, family history of a first-degree relative with a major congenital heart defect, and periconceptional multivitamin use.
Taksande *et al.*^50^	India (Asia)	Case-control	March 2004– April 2007	Hospital based	209 CHD cases and 418 controls	Infection	1st trimester	CHD	Matching variable: admitted during the same period.
Fung *et al.*^51^	Canada (North America)	Case-control	February 2008–July 2011	Hospital based	2 339 CHD cases and 199 controls	Infection (11.90%); rubella (0.36%); urinary tract infection (6.90%); other viral illness (3.81%)	1st trimester	CHD	Matching variable: same time period.
Adams *et al.*^52^	USA (North America)	Cohort study	March 1996– March 2007	Hospital based	*66* CHD cases and 853 non- CHD cases	Viral (adenovirus, cytomegalovirus, parvovirus B19, respiratory syncytial virus, enterovirus, and Epstein-Barr virus)	2nd trimester	CHD	NA
Oster *et al*.^53^	USA (North America)	Case-control	1981–1989	Population-based	2 361 CHD and 3 435 controls	Influenza	Periconceptional period	CHD; cardiac outflow defects; VSD, perimembranous; ASD; AVSDs; EA; right-sided obstructive defects; left-sided obstructive defects; total APVR	Matching variables: born in the region and frequency-matched to cases on month, year, hospital of birth, and age at interview. Adjusted for family history of CHD, infant sex, infant race, maternal age, maternal BMI, maternal gestational diabetes, maternal smoking, and maternal alcohol use.
Liu *et al*.^18^	China (Asia)	Case-control	January 2004– January 2005	Hospital based	164 CHD cases and 328 controls	Infection (11.59%);^a^ upper respiratory tract infection (20.93%)	Early pregnancy	CHD	Matching variables: same medical institutions during the same period, same sex, age difference of <1 year, and same geographic classification (rural or urban). Adjusted for mother’s education level, neonatal asphyxia or hypoxia, number of previous pregnancies, maternal upper respiratory tract infection, maternal infection, maternal B-mode ultrasound examination, and maternal mental stress.
Acs *et al*.^29^	Hungary (Europe)	Case-control	1980–1996	Population-based	4 479 CHD cases and 38 151 controls	Recurrent genital herpes	1st trimester; during pregnancy	CHD	Matching variables: sex, birth week and district of parents’ residence Adjusted for maternal age, birth order, maternal employment status, and acute maternal diseases.
Bánhidy^27^	Hungary (Europe)	Case-control	1980–1996	Population-based	4479 CHD cases and 38 151 controls	Urinary tract infection	1st trimester	CHD	Matching variables: sex, birth week and district of parents’ residence. Adjusted for maternal employment status, and use of ampicillin, cefalexin, nalidixic acid, nitrofurantoin, sulfamethoxazole 1 trimethoprim in the second and/or third months of pregnancy.
Acs *et al*.^28^	Hungary (Europe)	Case-control	1980–1996	Population-based	4 479 CHD cases and 38 151 controls	Acute respiratory infection	1st trimester; 2nd- 3rd trimester; during pregnancy	CHD	Matching variables: sex, birth week, and district of parents’ residence. Adjusted for maternal age, birth order, maternal employment status, influenza/common cold 1st trimester, and use of pregnancy supplements.
Botto *et al*.^54^	USA (North America)	Case-control	1982–1983	Population-based	905 CHD cases and 3 029 controls	Respiratory infection (5.96%);^a ^kidney infection (1.35%); gynecologic infection (0.20%)	Periconceptional period	CHD; cardiac outflow defects (conotruncal, included dTGA, TOF); VSD, perimembranous; ASD (all types); AVSDs (included with or without Down syndrome); EA; right-sided obstructive defects (included TA, PS, PA/intact ventricular septum); left-sided obstructive defects (included HLHS, AS, CoA); total APVR	Matching variables: same period, hospital of birth, calendar quarter of birth, and race. Adjusted for maternal race, education, multivitamin use, smoking, alcohol use, chronic illnesses, and child’s period of birth.
Roguin *et al.*^55^	Israel (Asia)	Case-control	April 1994–September 1994	Hospital based	56 VSD cases and 975 controls	Respiratory infection (3.78%); urinary tract infection (4.36%)^a^	1st trimester	VSD	NA
Tikkanen *et al.*^22^	Finland (Europe)	Case-control	1982–1984	Population-based	573 CHD cases and 1 055 controls	Upper respiratory infection	1st trimester	CHD	Matching variable: born in the same period.
Tikkanen *et al.*^23^	Finland (Europe)	Case-control	1982–1983	Population-based	408 CHD cases and 756 controls	Upper respiratory infection	1st trimester	All CHD; conus arteriosus; HLHS; other defects.	Matching variable: born in the same period. Adjusted for maternal age, maternal alcohol consumption, maternal smoking, maternal hypertension, maternal exposure to chemicals, organic solvents, dyes, lacquers or paints, mineral oil products, dusts, and glues at work, maternal deodorant use during the first trimester, and maternal ultrasound examination as appropriate.
Brown *et al*.^56^	USA (North America)	Case-control	NA	Hospital based	139 CHD cases and 262 controls	Coxsackie B1 (3.24%), B2 (5.24%), B3 (3.24%), B4 (7.73%),^a^ B5 (1.50%), A9 (6.73%); echoviruses 6 (1.00%), 9 (1.25%)	1st trimester; 2nd-3rd trimester; during pregnancy	CHD	Matching variables: born within 2 weeks, blood specimen collected within 2 weeks, maternal age, infant gender, and infant race.

This table shows the characteristic of 30 studies investigating maternal infection during the first trimester and risk of congenital heart defects in offspring. Main analyses were restricted to 26 low- or moderate-risk-bias studies that investigated the association between infections during the first trimester and overall congenital heart defects (with the blue background). Additional study characteristics are presented in Supplementary data online, Table S3. Nine studies of maternal infections at other/unspecific timepoints during pregnancy and risk of congenital heart defects in offspring are presented in Supplementary data online, Tables S2 and S3 (with the grey background). APVR, anomalous pulmonary venous return; AS, aortic stenosis; ASD, atrial septal defect; AVSD, atrioventricular septal defect; BMI, body mass index; CCHD, cyanotic congenital heart defects; CHD, congenital heart defects; CoA, coarctation of the aorta; DORV, double outlet right ventricle; EA, Ebstein’s anomaly; HLHS, hypoplastic left heart syndrome; IAA, interrupted aortic arch; NA, not applicable; OR, odds ratio; PA, pulmonary atresia; PDA, patent ductus arteriosus; PS, pulmonary stenosis; TA, tricuspid atresia; TGA, transposition of the great arteries; TOF, tetralogy of Fallot; TS, truncus stenosis; VSD, ventricular septal defect.

a Variable with the highest prevalence and used as the quantitative summary for any infection in pooled analyses (in studies reporting associations for more than one infection).

b To address the uncertainty in determining the exact conception date, we extended the exposure period to up to 3 months before conception. This includes early pregnancy (first-trimester or 1 month before conception) and the periconceptional period (1−3 months before conception).

**Table 2:** Subgroup analysis of any first-trimester maternal infection for overall congenital heart defects in offspring

Subgroup variables	Number of studies	Pooled OR (95% CI)	Measures of subgroup heterogeneity		
			*χ* ^2^	*P*	*I* ^2^
Study region			10.65^a^	.014^a^	71.83%^a^
Asia	13	2.12 (1.63, 2.75)	50.31	<.001	76.15%
Europe	6	1.33 (1.17, 1.50)	5.59	.348	10.57%
North America	6	1.35 (1.06, 1.72)	16.96	.005	70.51%
Oceania	1	1.68 (0.99, 2.84)	-	-	-
Study design			6.85^a^	.033^a^	70.80%^a^
Case-control	21	1.53 (1.32, 1.78)	73.66	<.001	72.85%
Cohort	3	2.24 (1.75, 2.87)	1.50	.474	0%
Cross-sectional	2	2.13 (0.74, 6.12)	10.00	.002	90.00%
Study setting			7.51^a^	.006^a^	86.68%^a^
Population-based	13	1.38 (1.20, 1.59)	41.36	<.001	70.99%
Hospital based	13	2.14 (1.62, 2.83)	38.19	<.001	68.58%
Infection assessment method			8.18^a^	.017^a^	75.55%^a^
Self-report	17	1.75 (1.44, 2.13)	82.97	<.001	80.72%
Medical records	4	1.28 (1.13, 1.44)	0.66	.883	0%
Laboratory testing	5	1.71 (1.15, 2.56)	9.72	.046	58.83%
Control for confounding			5.95^a^	.051^a^	66.39%^a^
Adjusted/matched for maternal age	14	1.44 (1.23, 1.68)	51.69	<.001	74.85%
Adjusted/matched for other covariates excluding maternal age	11	2.12 (1.57, 2.87)	36.30	<.001	72.45%
No adjustment/matching for any covariates	1	2.69 (0.89, 8.11)	-	-	-
Inclusion of pregnancy terminations or not			14.07^a^	<.001^a^	92.89%^a^
Yes	10	1.25 (1.12, 1.41)	15.14	.087	40.56%
No	16	2.08 (1.64, 2.63)	62.52	<.001	76.01%
Risk of bias			11.46^a^	<.001^a^	91.27%^a^
Low	14	1.37 (1.19, 1.57)	45.36	<.001	71.34%
Moderate	12	2.46 (1.80, 3.37)	34.62	<.001	68.23%
Diagnosis timing (*post hoc* analysis)			2.13^a^	.546^a^	0%^a^
Prenatal or at birth	3	1.94 (1.10, 3.40)	11.03	.004	81.87%
Within the first year	14	1.51 (1.30, 1.77)	34.71	<.001	62.54%
After the first year	3	2.09 (0.91, 4.79)	30.45	<.001	93.43%
Unspecified	6	2.01 (1.23, 3.28)	15.28	.009	67.27%

This table is based on the 26 studies with low or moderate risk of bias that investigated first-trimester maternal infection and overall congenital heart defects in offsprings. Pooled odds ratios (ORs) with 95% confidence intervals (CIs) are reported for each subgroup. Heterogeneity between subgroups was assessed using the Cochran’s *Q* test, its corresponding *P* value, and the *I*^2^ statistic. Heterogeneity measures were not calculated for subgroups with only one study (e.g. Oceania).

a Test for subgroup differences.

## Data Availability

The data underlying this article are available from the corresponding author upon reasonable request.

## Structured Graphical Abstract

Meta-analysis of any first-trimester maternal infection for overall congenital heart defects (CHD) in offspring, by specific type of infection. Summary forest plot presenting the association between specific type of maternal infection during the first-trimester of pregnancy and risk of overall CHD in the offspring. Studies that reported several groups of CHD were included using data from the group covering largest number of CHD or the group without extracardiac defects. Pooled odds ratios (OR) and their corresponding 95% confidence intervals (CI) were calculated using inverse variance weighted random-effects model. The quality score was calculated using the Newcastle-Ottawa Scale. Scores of 0-3, 4–6, and 7–9 are regarded as high, moderate, and low risk of bias, respectively. Main analyses were restricted to studies with low or moderate risk of bias.
