# Supplementary material for: First trimester maternal infections and offspring congenital heart defects: a meta-analysis
Source: Eur Heart J. Author manuscript; Available in PMC 2026 Feb 20. (PMC7618761; doi:10.1093/eurheartj/ehaf564)
Supplement: Supplementary data [file EMS212203-supplement-Supplementary_data.zip › Supplement_11Aug.pdf]

First trimester maternal infections and offspring congenital heart defects: a systematic review and meta-analysis

Supplementary Material

Supplementary Text S1. Post-hoc analysis on studies with fewer than 50 CHD cases 2

Table S1. Characteristics of five additional studies reporting fewer than 50 CHD cases 3

Table S2. Full search strategy in Embase, PubMed, Web of Science, Scopus, and the Cochrane Library 5

Table S3. Characteristic of 9 studies of maternal infections at other/unspecific timepoints during pregnancy and risk of congenital heart defects in offspring 22

Table S4. Detailed characteristics of 39 included studies of maternal infections and risk of congenital heart defects in offspring 25

Table S5. Quality assessment of 39 included studies using the Newcastle-Ottawa Scale 26

Table S6. Sensitivity analysis of any maternal infection for overall congenital heart defects in offspring 29

Figure S1. Funnel plots of any first trimester maternal infection for overall congenital heart defects in offspring: A) observed studies; B) together with imputed studies 30

Figure S2. Meta-analysis of any first trimester maternal infection for congenital heart defects in offspring, by specific type of heart defects 31

Figure S3. Meta-analysis of any first trimester maternal infection for overall congenital heart defects in offspring, by subgroups 37

Figure S4. Meta-analysis of any first trimester maternal infection for congenital heart defects without extracardiac defects and with extracardiac defects among the same population 38

Figure S5. Leave-one-out sensitivity analysis 39

Figure S6. Post-hoc risk-of-bias assessment of 39 included studies using the Risk Of Bias In Non-randomized Studies - of Exposure tool 40

Supplementary Text S1. Post-hoc analysis on studies with fewer than 50 CHD cases

Our pre-specified study protocol foresaw the exclusion of studies reporting fewer than 50 congenital heart defect (CHD) cases. This threshold was based on empirical guidance from previous meta-analyses, with the aim to improve the stability and precision of pooled effect estimates and ensure adequate statistical information and comparability across studies.

To further examine the potential for publication bias introduced by excluding small studies, we conducted a post-hoc sensitivity analysis that included studies originally excluded solely due to having fewer than 50 CHD cases, but which otherwise met all eligibility criteria.

We identified five studies with fewer than 50 CHD cases and otherwise meeting all eligibility criteria (characteristics in **Table S1**). Among them, three (Garand-2023, Calvert-2023, and Ma-2017) investigated maternal infections during the first trimester and were included in the post-hoc sensitivity analysis. The remaining two studies (Lai-1998 and Coffey-1963) did not meet the exposure timing criterion (i.e., did not focus on first-trimester infections) and were not used in the sensitivity analysis.

Including these three additional studies led to a pooled odds ratio of 1.66 (95% CI: 1.43, 1.91), an estimate highly consistent with main analyses (Odds ratio: 1.63; 95% CI: 1.41, 1.88).

Table S1. Characteristics of studies reporting fewer than 50 CHD cases and otherwise meeting all eligibility criteria

Study	Study region	Study design	Study period	Study setting	Sample size	Investigated exposures	Exposure timing	Reported outcomes	Matching/adjusting variables	Results[‡]	Quality score[§]
Garand et al. (2023) [1]	USA (North America)	Case-control	NA	Population-based	16 CHD cases and 88 controls	Coxsackievirus B	1st trimester	CHD	NA	4.545 (1.259, 16.385)	6
Calvert et al. (2023) [2]	UK (Europe)	Case-control	May 2020-April 2022	Population-based	29 CHD cases and 6267 non-CHD cases	SARS-CoV-2 infection	From 6 weeks before pregnancy to 19 weeks and 6 days of gestation	CHD	Matching variables: maternal age at conception, gestational week of infection, and season of conception	0.955 (0.407, 2.242)	8
Ma et al. (2017) [3]	China (Asia)	Case-control	NA	Hospital based	32 CHD cases and 32 controls	Upper respiratory infection (23.44%);[†] Hepatitis B (9.38%)	Early pregnancy	CHD	Matching variables: sex and age difference within 1 year	Upper respiratory infection: 5.800 (1.446, 23.195) Hepatitis B: 0.467 (0.079, 2.753)	6
Lai et al. (1998) [4]	USA (North America)	Cohort study	May 1990-Jan. 1994	Population-based	43 CHD cases and 496 non-CHD cases	HIV	During pregnancy	CHD	NA	1.356 (0.627, 2.933)	7
Coffey et al. (1963) [5]	Ireland (Europe)	Cohort study	1957-1962	Population-based	9 CHD cases and 1297 non-CHD cases	Maternal influenza	During pregnancy	CHD	NA	1.252 (0.334, 4.679)	5

This table shows the characteristics of 5 additional studies reporting fewer than 50 CHD cases and otherwise meeting all eligibility criteria. Among them, three studies (Garand-2023, Calvert-2023, and Ma-2017) were incorporated into a post-hoc sensitivity analysis.

CHD: congenital heart defects; NA: not applicable.

[†] Variable with the highest prevalence and used as the quantitative summary for any infection in pooled analyses (in studies reporting associations for more than one infection).

[‡] Crude odds ratio calculated by the authors.

[§] Risk of bias was assessed using the Newcastle-Ottawa Scale. Scores of 0-3 indicate a high risk of bias, scores of 4-6 indicate a moderate risk of bias, and scores of 7-9 indicate a low risk of bias.

References:

1. Garand M, Huang SSY, Goessling LS, Wan F, Santillan DA, Santillan MK, et al. Virome Analysis and Association of Positive Coxsackievirus B Serology during Pregnancy with Congenital Heart Disease. Microorganisms 2023;11. doi: 10.3390/microorganisms11020262

2. Calvert C, Carruthers J, Denny C, Donaghy J, Hopcroft LEM, Hopkins L, et al. A population-based matched cohort study of major congenital anomalies following COVID-19 vaccination and SARS-CoV-2 infection. Nature Communications 2023;14. doi: 10.1038/s41467-022-35771-8

3. Ma FQ, Zhang GY, Gao JB, Fang C, Bo Y, Huang GY, et al. Clinical screening method and risk factors' analysis of congenital cardiovascular defects: A case control study from a Chinese local region. Clinical and Experimental Obstetrics and Gynecology 2017;44:515-520. doi: 10.12891/ceog3369.2017

4. Lai WW, Lipshultz SE, Easley KA, Starc TJ, Drant SE, Timothy Bricker J, et al. Prevalence of congenital cardiovascular malformations in children of human immunodeficiency virus-infected women: The prospective P2C2 HIV multicenter study. Journal of the American College of Cardiology 1998;32:1749-1755. doi: 10.1016/S0735-1097(98)00449-5

5. Coffey VP, Jessop WJ. Maternal influenza and congenital deformities. A follow-up study. Lancet 1963;1:748-751. doi: 10.1016/s0140-6736(63)91567-8

Table S2. Full search strategy in Embase, PubMed, Web of Science, Scopus, and the Cochrane Library

Embase: Embase.com<br><br>(Concept 1) AND (Concept 2) AND (Concept 3) NOT 'conference abstract':it<br><br>Concept 1:<br><br>('congenital heart disease'/de OR 'congenital heart malformation'/de OR ‘Congenital Heart Defect*’:ti,ab,kw OR ‘Congenital Heart Disease*’:ti,ab,kw OR ‘Congenital Cardiac Disease*’:ti,ab,kw OR ‘Congenital Cardiac Distress*’:ti,ab,kw OR ‘Congenital Heart Distress*’:ti,ab,kw OR ‘Congenital Heart Failure’:ti,ab,kw OR ‘Heart Congenital Disease*’:ti,ab,kw OR 'Heart Congenital Defect*’:ti,ab,kw OR 'Heart Congenital Anomal*’:ti,ab,kw OR 'Heart Congenital Malformation*’:ti,ab,kw OR ‘Neonatal Cardiopathy’:ti,ab,kw OR ‘Congenital Heart Malformation*’:ti,ab,kw OR ‘Congenital Malformations of the Heart’:ti,ab,kw OR ‘Congenital Malformation of the Heart’:ti,ab,kw OR ‘Congenital Heart Anomal*’:ti,ab,kw OR ‘Congenital Cardiac Defect*’:ti,ab,kw OR ‘Congenital Cardiac Malformation*’:ti,ab,kw OR ‘Congenital Cardiac Anomal*’:ti,ab,kw OR ‘Congenital Cardiovascular Disease*’:ti,ab,kw) OR ('heart atrium septum defect'/exp OR 'Atrial Septal Defect*’:ti,ab,kw OR 'Atrium Septum Defect*’:ti,ab,kw OR ‘Persistent Ostium Primum’:ti,ab,kw OR 'Atrial Heart Septal Defect*’:ti,ab,kw OR ‘Atrial Heart Shunt’:ti,ab,kw OR ‘Atrial Septum Defect*’:ti,ab,kw OR 'Atrium Heart Septal Defect*':ti,ab,kw OR 'Atrial Heart Septum Defect*':ti,ab,kw OR ‘Atrium Heart Septum Defect*’:ti,ab,kw OR ‘Atrium Septal Defect*’:ti,ab,kw OR ‘Atrium Septum Primum Defect*’:ti,ab,kw OR 'Atrial Septum Primum Defect*':ti,ab,kw OR ‘Atrium Septum Secundum Defect*’:ti,ab,kw OR 'Atrial Septum Secundum Defect*':ti,ab,kw OR ‘Cleft Heart Atrium’:ti,ab,kw OR ‘Interatrial Septal Defect*’:ti,ab,kw OR ‘Interatrial Septum Defect*’:ti,ab,kw OR ‘Interauricular Septal Defect*’:ti,ab,kw OR ‘Lutembacher*’:ti,ab,kw OR ‘Patent Ostium Secundum’:ti,ab,kw OR ‘Persistent Ostium Secundum’:ti,ab,kw OR ‘Secundum Atrial Defect*’:ti,ab,kw) OR ('heart ventricle septum defect'/exp OR ‘Ventricular Septal Defect*’:ti,ab,kw OR ‘Intraventricular Septal Defect*’:ti,ab,kw OR ‘Intraventricular Septum Defect*’:ti,ab,kw OR ‘Ventricle Septal Defect*’:ti,ab,kw OR ‘Ventricular Septum Defect*’:ti,ab,kw OR ‘Ventricle Septum Defect*’:ti,ab,kw OR ‘Interventricular Septal Defect*’:ti,ab,kw OR ‘Interventricular Septum Defect*’:ti,ab,kw OR ‘Ventricular Heart Septal Defect*’:ti,ab,kw OR ‘Ventricular Heart Septum Defect*’:ti,ab,kw OR ‘Ventricle Heart Septal Defect*’:ti,ab,kw OR ‘Ventricle Heart Septum Defect*’:ti,ab,kw OR ‘Ventricular Septal Perforation*’:ti,ab,kw OR ‘Ventricular Septum Perforation*’:ti,ab,kw OR ‘Ventricle Septal Perforation*’:ti,ab,kw OR ‘Ventricle Septum Perforation*’:ti,ab,kw OR ‘Interventricular Shunt*’:ti,ab,kw OR ‘Intraventricular Shunt*’:ti,ab,kw OR ‘Membranous Incomplete Sept*’:ti,ab,kw) OR ('aortic coarctation'/exp OR ‘Aortic Coarctation*’:ti,ab,kw OR ‘Aorta Coarctation*’:ti,ab,kw OR ‘Coarctation of Aorta’:ti,ab,kw OR ‘Coarctation of the Aorta’:ti,ab,kw OR ‘Aorta Dominant Coarctation*’:ti,ab,kw OR ‘Aortic Isthmus Stenosis':ti,ab,kw OR ‘Coarctatio Aortae’:ti,ab,kw) OR ('atrioventricular septal defect'/exp OR ‘Atrioventricular Septal Defect*':ti,ab,kw OR ‘Atrioventricular Canal Defect*':ti,ab,kw) OR ('heart right ventricle double outlet'/exp OR ‘Double Outlet Right Ventricle*’:ti,ab,kw OR ‘Taussig-Bing Anomal*’:ti,ab,kw OR ‘Double Outlet Right Heart Ventricle’:ti,ab,kw OR ‘Heart Ventricle Double Outlet Right’:ti,ab,kw OR ‘Right Heart Ventricle Double Outlet’:ti,ab,kw OR ‘Right Ventricle Double Outlet’:ti,ab,kw OR ‘Right Ventricular Double Outlet’:ti,ab,kw OR ‘Heart Right Ventricle Double Outlet’:ti,ab,kw) OR ('Ebstein anomaly'/exp OR ‘Ebstein*’:ti,ab,kw) OR ('hypoplastic left heart syndrome'/exp OR ‘Left Heart Hypoplasia Syndrome’:ti,ab,kw OR ‘Hypoplastic Left Heart Syndrome’:ti,ab,kw) OR ('aortic arch interruption'/exp OR 'Aortic Arch Interruption*’:ti,ab,kw OR 'Aorta Arch Interruption*’:ti,ab,kw) OR ('patent ductus arteriosus'/exp OR 'Patent Ductus Arterios*’:ti,ab,kw OR ‘Patency of the Ductus Arteriosus’:ti,ab,kw OR ‘Ductus Arteriosus Patency’:ti,ab,kw OR ‘Ductus Arteriosus Persisten*’:ti,ab,kw OR ‘Open Ductus Botalli’:ti,ab,kw OR ‘Patent Ductus Botalli’:ti,ab,kw OR ‘Persistent Ductus Arteriosus’:ti,ab,kw OR ‘Persistent Ductus Botalli’:ti,ab,kw OR ‘Truncus Arteriosus Persistens’:ti,ab,kw) OR ('pulmonary valve stenosis'/exp OR ‘Pulmonic Stenos*’:ti,ab,kw OR ‘Pulmonary Stenos*’:ti,ab,kw OR ‘Pulmonary Valve Stenos*’:ti,ab,kw OR ‘Pulmonary Valva Stenosis’:ti,ab,kw OR ‘Pulmonary Valva Stenoses’:ti,ab,kw OR ‘Pulmonal Stenos*’:ti,ab,kw OR ‘Lung Artery Valve Stenosis’:ti,ab,kw OR ‘Lung Artery Valve Stenoses’:ti,ab,kw OR ‘Lung Artery Valvular Stenosis’:ti,ab,kw OR ‘Lung Artery Valvular Stenoses’:ti,ab,kw OR ‘Lung Valve Stenosis’:ti,ab,kw OR ‘Lung Valve Stenoses’:ti,ab,kw) OR ('pulmonary valve stenosis'/exp OR ‘Pulmonary Atresia*’:ti,ab,kw OR ‘Pulmonary Valve Atresia*’:ti,ab,kw OR ‘Pulmonic Atresia*’:ti,ab,kw OR ‘Pulmonic Valve Atresia*’:ti,ab,kw OR ‘Pulmonary Artery Atresia*’:ti,ab,kw OR ‘Pulmonic Artery Atresia*’:ti,ab,kw OR ‘Lung Atresia*’:ti,ab,kw OR ‘Lung Artery Atresia*’:ti,ab,kw OR ‘Lung Valve Atresia*’:ti,ab,kw OR ‘Lung Artery Valve Atresia*’:ti,ab,kw) OR ('heart single ventricle'/exp OR ‘Univentricular Heart*’:ti,ab,kw OR ‘Complex Single Ventricle*’:ti,ab,kw OR ‘Single Heart Ventricle*’:ti,ab,kw OR ‘Heart Single Ventricle*’:ti,ab,kw OR

‘Monoventricular Heart*’:ti,ab,kw OR ‘Cor Monoventriculare’:ti,ab,kw OR ‘Cor Triloculare Bia*’:ti,ab,kw) OR ('Fallot tetralogy'/exp OR ‘Fallot*’:ti,ab,kw) OR ('lung vein drainage anomaly'/exp OR 'scimitar syndrome'/exp OR 'Scimitar Syndrome':ti,ab,kw OR ‘Scimitar Anomaly’:ti,ab,kw OR ‘Anomalous Pulmonary Venous Return’:ti,ab,kw OR ‘Pulmonary Venous Return Anomaly’:ti,ab,kw OR ‘Anomalous Lung Vein Drainage’:ti,ab,kw OR ‘Anomalous Pulmonary Vein Drainage’:ti,ab,kw OR ‘Anomalous Pulmonary Venous‘:ti,ab,kw OR ‘Lung Venous Drainage Anomaly’:ti,ab,kw OR ‘Lung Vein Drainage Anomaly’:ti,ab,kw OR ‘Lung Venous Return Anomaly’:ti,ab,kw OR ‘Pulmonary Venous Drainage Anomaly’:ti,ab,kw) OR ('great vessels transposition'/exp OR ‘Transposition of Great Arter*’:ti,ab,kw OR ‘Transposition of Great Vessel*’:ti,ab,kw OR ‘Great Vessels Transposition*’:ti,ab,kw OR ‘Great Vessel Transposition*’:ti,ab,kw OR ‘Great Arteries Transposition*’:ti,ab,kw OR ‘Great Artery Transposition*’:ti,ab,kw OR ‘Great Vessels Dextrotransposition*’:ti,ab,kw OR ‘Great Vessels Levotransposition*’:ti,ab,kw OR ‘Levotransposition of Great Vessel*’:ti,ab,kw OR ‘Dextrotransposition of Great Vessel*’:ti,ab,kw OR ‘Dextro-Looped Transposition of the Great Arter*’:ti,ab,kw OR ‘Dextro Looped Transposition of the Great Arter*’:ti,ab,kw OR ‘Levo-Looped Transposition of the Great Arter*’:ti,ab,kw OR ‘Large Vessel Transposition*’:ti,ab,kw) OR ('tricuspid valve atresia'/exp OR ‘Tricuspid Atresia*’:ti,ab,kw OR ‘Tricuspid Valve Atresia*’:ti,ab,kw OR ‘Absent Right Atrioventricular Connection*’:ti,ab,kw OR ‘Right Atrioventricular Cardiac Valve Atresia*’:ti,ab,kw OR ‘Right Atrioventricular Cardiac Valvular Atresia*’:ti,ab,kw OR ‘Right Atrioventricular Heart Valve Atresia*’:ti,ab,kw OR ‘Right Atrioventricular Heart Valvular Atresia*’:ti,ab,kw OR ‘Right Atrioventricular Valve Atresia*’:ti,ab,kw OR ‘Right Atrioventricular Valvular Atresia*’:ti,ab,kw OR ‘Tricuspid Cardiac Valve Atresia*’:ti,ab,kw OR ‘Tricuspid Heart Valve Atresia*’:ti,ab,kw OR ‘Tricuspid Valvular Atresia*’:ti,ab,kw) OR ('arterial trunk'/exp OR ‘Arterial Trun*’:ti,ab,kw OR ‘Truncus Arteri*’:ti,ab,kw) OR ('aortic valve stenosis'/exp OR ‘Aortic Valve Stenos*’:ti,ab,kw OR ‘Aortic Stenos*’:ti,ab,kw OR ‘Aorta Valve Stenos*’:ti,ab,kw OR ‘Aortic Valvular Stenos*’:ti,ab,kw OR ‘Stenosed Aortic Valve*’:ti,ab,kw OR ‘Stenotic Aortic Valve*’:ti,ab,kw)

Concept 2:

('infection'/de OR ‘Infect*’:ti,ab,kw OR ‘Infest*’:ti,ab,kw OR ‘Inflammation*’:ti,ab,kw OR ‘TORCH*’:ti,ab,kw) OR ('coronavirus disease 2019'/exp OR nCoV:ti,ab,kw,ad,ff OR 2019nCoV:ti,ab,kw,ad,ff OR COVID:ti,ab,kw,ad,ff OR COVID19:ti,ab,kw,ad,ff OR 'Severe acute respiratory syndrome coronavirus 2'/exp OR 'severe acute respiratory syndrome 2':ti,ab,kw,ff OR 'sars cov 2':ti,ab,kw,ad,ff OR SARS2:ti,ab,kw,ad,ff OR 'cov 2':ti,ab,kw,ad,ff OR cov2:ti,ab,kw,ad,ff OR coronavirus*:ti,ab,kw,ad,ff OR ‘corona virus*’:ti,ab,kw,ad,ff OR 'betacoronavirus*':ti,ab,kw,ad,ff OR ‘wuhan virus’:ti,ab,kw OR ((wuhan:ti,ab,kw,ad,ff OR novel:ti,ab,kw,ad,ff OR 19:ti,ab,kw,ad,ff OR 2019:ti,ab,kw,ad,ff OR epidem*:ti,ab,kw OR epidemy:ti,ab,kw,ff OR epidemic*:ti,ab,kw,ad,ff OR pandem*:ti,ab,kw,ad,ff OR outbreak:ti,ab,kw,ad,ff OR new:ti,ab,kw,ad,ff) AND ('pneumonia virus*':ti,ab,kw OR cov:ti,ab,kw OR hcov:ti,ab,kw))) OR ('rubella'/exp OR 'Rubella virus'/exp OR ‘Rubella*’:ti,ab,kw OR ‘Three Day Measle*’:ti,ab,kw OR ‘German Measle*’:ti,ab,kw OR ‘Epidemic Roseola’:ti,ab,kw) OR ('hepatitis virus'/exp OR 'virus hepatitis'/exp OR 'Virus Hepatiti*':ti,ab,kw OR 'Viral Hepatiti*':ti,ab,kw OR 'Virus Liver Disease*':ti,ab,kw OR 'Viral Liver Disease*':ti,ab,kw OR 'Hepatitis Virus Infection*':ti,ab,kw OR 'Hepatitis Viral Infection*':ti,ab,kw OR 'Hepatitis due to Virus*':ti,ab,kw OR 'Hepatitis Caused by Virus*':ti,ab,kw) OR ('Human cytomegalovirus'/exp OR 'Cytomegalovirus*':ti,ab,kw OR 'Salivary Gland Virus*':ti,ab,kw OR 'Beta Herpes Virus 5’:ti,ab,kw OR ‘Human Herpesvirus 5’:ti,ab,kw OR ‘Human Herpesvirus V’:ti,ab,kw OR ‘Human Herpesvirus Type 5’:ti,ab,kw OR ‘Human Herpesvirus Type V’:ti,ab,kw OR ‘Herpes Simplex Virus 5’:ti,ab,kw OR ‘Herpes Simplex Virus V’:ti,ab,kw OR ‘Herpes Simplex Virus Type 5’:ti,ab,kw OR ‘Herpes Simplex Virus Type V’:ti,ab,kw OR ‘Herpes Virus 5’:ti,ab,kw OR ‘Herpes Virus V’:ti,ab,kw OR ‘Herpes Virus Type 5’:ti,ab,kw OR ‘Herpes Virus Type V’:ti,ab,kw) OR ('Human alphaherpesvirus 1'/exp OR ‘Human Alphaherpesvirus 1’:ti,ab,kw OR ‘Human Herpesvirus 1’:ti,ab,kw OR ‘Human Herpesvirus I’:ti,ab,kw OR ‘Human Herpesvirus Type 1’:ti,ab,kw OR ‘Human Herpesvirus Type I’:ti,ab,kw OR ‘Herpes Simplex Virus 1’:ti,ab,kw OR ‘Herpes Simplex Virus I’:ti,ab,kw OR ‘Herpes Simplex Virus Type 1’:ti,ab,kw OR ‘Herpes Simplex Virus Type I’:ti,ab,kw OR ‘Herpes Virus 1’:ti,ab,kw OR ‘Herpes Virus I’:ti,ab,kw OR ‘Herpes Virus Type 1’:ti,ab,kw OR ‘Herpes Virus Type I’:ti,ab,kw) OR ('Herpes simplex virus 2'/exp OR ‘Human Herpesvirus 2’:ti,ab,kw OR ‘Human Herpesvirus II’:ti,ab,kw OR ‘Human Herpesvirus Type 2’:ti,ab,kw OR ‘Human Herpesvirus Type II’:ti,ab,kw OR ‘Herpes Simplex Virus 2’:ti,ab,kw OR ‘Herpes Simplex Virus II’:ti,ab,kw OR ‘Herpes Simplex Virus Type 2’:ti,ab,kw OR ‘Herpes Simplex Virus Type II’:ti,ab,kw OR ‘Herpes Virus 2’:ti,ab,kw OR ‘Herpes Virus II’:ti,ab,kw OR ‘Herpes Virus Type 2’:ti,ab,kw OR ‘Herpes Virus Type II’:ti,ab,kw) OR ('Epstein Barr virus'/exp OR ‘Human Herpesvirus 4’:ti,ab,kw OR ‘Human Herpesvirus IV’:ti,ab,kw OR ‘Human Herpesvirus Type 4’:ti,ab,kw OR ‘Human Herpesvirus Type IV’:ti,ab,kw OR ‘Herpes Simplex Virus 4’:ti,ab,kw OR ‘Herpes Simplex Virus IV’:ti,ab,kw OR ‘Herpes Simplex Virus Type 4’:ti,ab,kw OR ‘Herpes Simplex Virus Type IV’:ti,ab,kw OR ‘Herpes Virus

4’:ti,ab,kw OR ‘Herpes Virus IV’:ti,ab,kw OR ‘Herpes Virus Type 4’:ti,ab,kw OR ‘Herpes Virus Type IV’:ti,ab,kw OR ‘Burkitt Herpesvirus’:ti,ab,kw OR ‘Burkitt Lymphoma Virus’:ti,ab,kw OR ‘Burkitts Lymphoma Virus’:ti,ab,kw OR ‘Burkitts Lymphoma Virus’:ti,ab,kw OR ‘Infectious Mononucleosis Virus*’:ti,ab,kw OR ‘Infectious Mononucleosis Herpetovirus’:ti,ab,kw OR ‘Mononucleosis Infectiosa Virus’:ti,ab,kw OR ‘Epstein-Barr Virus*’:ti,ab,kw OR ‘Epstein Barr Virus*’:ti,ab,kw OR ‘Barr Epstein Virus’:ti,ab,kw OR ‘Epstein Barr Herpetovirus’:ti,ab,kw OR ‘Epstein Virus’:ti,ab,kw) OR ('Varicella zoster virus'/exp OR ‘Human Herpesvirus 3’:ti,ab,kw OR ‘Human Herpesvirus III’:ti,ab,kw OR ‘Human Herpesvirus Type 3’:ti,ab,kw OR ‘Human Herpesvirus Type III’:ti,ab,kw OR ‘Herpes Simplex Virus 3’:ti,ab,kw OR ‘Herpes Simplex Virus III’:ti,ab,kw OR ‘Herpes Simplex Virus Type 3’:ti,ab,kw OR ‘Herpes Simplex Virus Type III’:ti,ab,kw OR ‘Herpes Virus 3’:ti,ab,kw OR ‘Herpes Virus III’:ti,ab,kw OR ‘Herpes Virus Type 3’:ti,ab,kw OR ‘Herpes Virus Type III’:ti,ab,kw OR ‘Chickenpox’:ti,ab,kw OR ‘Chicken Pox’:ti,ab,kw OR ‘Ocular Herpes Zoster Virus’:ti,ab,kw OR ‘Shingles’:ti,ab,kw OR ‘Zona Virus’:ti,ab,kw OR ‘Varicella-Zoster Virus*’:ti,ab,kw OR ‘Varicella Zoster’:ti,ab,kw OR ‘Varicella Foster Virus’:ti,ab,kw OR ‘Varicella Herpes Zoster Virus’:ti,ab,kw OR ‘Varicella Herpetovirus’:ti,ab,kw OR ‘Varicella Pneumonia Virus’:ti,ab,kw OR ‘Varicella Virus’:ti,ab,kw OR ‘Herpesvirus Varicellae’:ti,ab,kw OR ‘Herpes Zoster Virus*’:ti,ab,kw OR ‘Herpes Virus Varicellae’:ti,ab,kw OR ‘Herpes Zoster Varicella Virus’:ti,ab,kw) OR ('Lymphocytic choriomeningitis virus'/exp OR ‘Chorio Meningitis’:ti,ab,kw OR ‘Choriomeningitis’:ti,ab,kw) OR ('Zika virus'/exp OR ‘Zika Virus*’:ti,ab,kw OR ‘Zika Flavivirus*’:ti,ab,kw) OR ('West Nile virus'/exp OR ‘West Nile Virus*’:ti,ab,kw OR ‘Egypt 101 Virus*’:ti,ab,kw OR ‘Kunjin Virus*’:ti,ab,kw OR ‘West Nile Viral Disease’:ti,ab,kw OR ‘West Nile Flavivirus Infection’:ti,ab,kw OR ‘West Nile Infection*’:ti,ab,kw OR ‘West Nile Viral Infection*’:ti,ab,kw) OR ('Rift Valley fever virus'/exp OR ‘Rift Valley fever':ti,ab,kw OR ‘rift valley virus*':ti,ab,kw) OR ('Human parvovirus B19'/exp OR ‘B19 Virus':ti,ab,kw OR ‘Parvovirus B19*':ti,ab,kw) OR ('Human immunodeficiency virus'/exp OR ‘Human Immunodeficiency Virus*’:ti,ab,kw OR ‘Human T Cell Lymphotropic Virus Type III’:ti,ab,kw OR ‘Human T-Cell Leukemia Virus Type III’:ti,ab,kw OR ‘LAV-HTLV-III’:ti,ab,kw OR ‘Lymphadenopathy-Associated Virus*’:ti,ab,kw OR ‘Human T Lymphotropic Virus Type III’:ti,ab,kw OR ‘Acquired Immune Deficiency Syndrome’:ti,ab,kw OR ‘Acquired Immunodeficiency Syndrome’:ti,ab,kw OR ‘Aids Associated Lentivirus’:ti,ab,kw OR ‘Aids Associated Retrovirus’:ti,ab,kw OR ‘Aids Associated Virus’:ti,ab,kw OR ‘Aids Related Virus’:ti,ab,kw OR ‘Human Immuno Deficiency Virus’:ti,ab,kw OR ‘Immunodeficiency Associated Virus’:ti,ab,kw OR ‘Lymphadenopathy Associated Retrovirus’:ti,ab,kw) OR ('Coxsackie virus infection'/exp OR 'Coxsackievir*’:ti,ab,kw OR 'Coxsackie Vir*’:ti,ab,kw) OR ('Listeria monocytogenes'/exp OR ‘Listeria Monocytogenes’:ti,ab,kw OR ‘Listerella Hepatolytica’:ti,ab,kw OR ‘Listeriosis Monocytogenes’:ti,ab,kw OR ‘Bacterium Monocytogenes’:ti,ab,kw OR ‘Corynebacterium Infantisepticum’:ti,ab,kw OR ‘Corynebacterium Parvulum’:ti,ab,kw OR ‘Erysipelothrix Monocytogenes’:ti,ab,kw) OR ('Treponema pallidum'/exp OR ‘Treponema Pallid*':ti,ab,kw OR ‘Treponema Reiterii':ti,ab,kw OR ‘Spirochaeta Pallida':ti,ab,kw) OR ('Streptococcus agalactiae'/exp OR ‘Streptococcus B’:ti,ab,kw OR ‘Streptococcus Group B’:ti,ab,kw OR ‘Group B Streptococcus’:ti,ab,kw OR ‘Staphylococcus Agalactiae’:ti,ab,kw OR ‘Streptococcus Mastitidis’:ti,ab,kw OR ‘Streptococcus Nocardi’:ti,ab,kw OR ‘Streptococcus Agalactiae’:ti,ab,kw) OR ('Staphylococcus aureus'/exp OR ‘Staphylococcus Aureus’:ti,ab,kw OR ‘Staphylococcus Pyogenes Aureus’:ti,ab,kw OR ‘Staphylococcus Pyogenes citreus’:ti,ab,kw OR ‘Micrococcus Aureus’:ti,ab,kw OR ‘Micrococcus Pyogenes’:ti,ab,kw) OR ('Escherichia coli'/exp OR ‘Enterococcus Coli’:ti,ab,kw OR ‘Escherichia Coli’:ti,ab,kw OR ‘Bacillus Coli’:ti,ab,kw OR ‘Bacterium Coli’:ti,ab,kw OR ‘Alkalescens-Dispar Group’:ti,ab,kw OR ‘EAggEC’:ti,ab,kw OR ‘Bacillus Escherichii’:ti,ab,kw OR ‘Bacterium E3’:ti,ab,kw OR ‘Coli Bacillus’:ti,ab,kw OR ‘Coli Bacterium’:ti,ab,kw OR ‘Colibacillus’:ti,ab,kw OR ‘Colon Bacillus’:ti,ab,kw OR ‘Escherichia Alkalescens Dispart’:ti,ab,kw) OR ('Chlamydia trachomatis'/exp OR ‘Chlamydia Trachomatis’:ti,ab,kw OR ‘Chlamidia Trachomatis’:ti,ab,kw OR ‘Chlamydophila Trachomatis’:ti,ab,kw OR ‘Chlamydozoon Trachomatis’:ti,ab,kw OR ‘Rickettsia Trachomae’:ti,ab,kw OR ‘Rickettsia Trachomatis’:ti,ab,kw) OR ('Helicobacter pylori'/exp OR ‘Helicobacter Pylori’:ti,ab,kw OR ‘Helicobacter Nemestrinae’:ti,ab,kw OR ‘Campylobacter Pylori’:ti,ab,kw) OR ('Plasmodium falciparum'/exp OR 'Plasmodium Falciparum*’:ti,ab,kw) OR ('Plasmodium vivax'/exp OR ‘Plasmodium vivax*’:ti,ab,kw) OR ('Toxoplasma'/exp OR ‘Toxoplasma*’:ti,ab,kw OR ‘Toxoplasma Gondi*':ti,ab,kw OR ‘Toxoplasma Hominis':ti,ab,kw) OR ('Trypanosoma cruzi'/exp OR ‘Trypanosoma Cruz':ti,ab,kw OR ‘Schizotrypanum Cruzi':ti,ab,kw)

Concept 3:

'maternal exposure'/exp OR 'prenatal exposure delayed effect'/exp OR ‘Maternal’:ti,ab,kw OR ‘Transplacental':ti,ab,kw OR ‘Prenatal’:ti,ab,kw OR ‘Mother*’:ti,ab,kw OR ‘Pregnan*’:ti,ab,kw OR ‘Women’:ti,ab,kw OR ‘Woman’:ti,ab,kw OR ‘Female’:ti,ab,kw OR ‘In Utero’:ti,ab,kw OR ‘Uteroplacental’:ti,ab,kw

PubMed: (nih.gov)

(Concept 1) AND (Concept 2) AND (Concept 3)

Concept 1:

("Heart Defects, Congenital"[Mesh:NoExp] OR "Congenital Heart Defect*"[tiab] OR "Congenital Heart Disease*"[tiab] OR "Congenital Cardiac Disease*"[tiab] OR "Congenital Cardiac Distress"[tiab:~0] OR "Congenital Cardiac Distresses"[tiab:~0] OR "Congenital Heart Distress"[tiab:~0] OR "Congenital Heart Distresses"[tiab:~0] OR "Congenital Heart Failure"[tiab] OR "Heart Congenital Disease*"[tiab] OR "Heart Congenital Defect*"[tiab] OR "Heart Congenital Anomaly"[tiab:~0] OR "Heart Congenital Anomalies"[tiab:~0] OR "Heart Congenital Malformation*"[tiab] OR "Neonatal Cardiopathy"[tiab:~0] OR "Congenital Heart Malformation*"[tiab] OR "Congenital Malformation of the Heart"[tiab] OR "Congenital Malformations of the Heart"[tiab] OR "Congenital Heart Anomal*"[tiab] OR "Congenital Cardiac Defect*"[tiab] OR "Congenital Cardiac Malformation*"[tiab] OR "Congenital Cardiac Anomal*"[tiab] OR "Congenital Cardiovascular Disease*"[tiab]) OR ("Heart Septal Defects, Atrial"[Mesh] OR "Atrial Septal Defect*"[tiab] OR "Atrium septum defect*"[tiab] OR "Persistent Ostium Primum"[tiab] OR "Atrial Heart Septal Defect*"[tiab] OR "Atrial Heart Shunt"[tiab:~0] OR "Atrial Septum Defect*"[tiab] OR "Atrium Heart Septal Defect"[tiab:~0] OR "Atrium Heart Septal Defects"[tiab:~0] OR "Atrial Heart Septum Defect"[tiab:~0] OR "Atrial Heart Septum Defects"[tiab:~0] OR "Atrium Heart Septum Defect"[tiab:~0] OR "Atrium Heart Septum Defects"[tiab:~0] OR "Atrium Septal Defect*"[tiab] OR "Atrium Septum Primum Defect"[tiab:~0] OR "Atrium Septum Primum Defects"[tiab:~0] OR "Atrial Septum Primum Defect"[tiab:~0] OR "Atrial Septum Primum Defects"[tiab:~0] OR "Atrium Septum Secundum Defect"[tiab:~0] OR "Atrium Septum Secundum Defects"[tiab:~0] OR "Atrial Septum Secundum Defect"[tiab:~0] OR "Atrial Septum Secundum Defects"[tiab:~0] OR "Cleft Heart Atrium"[tiab:~0] OR "Interatrial Septal Defect*"[tiab] OR "Interatrial Septum Defect*"[tiab] OR "Interauricular Septal Defect*"[tiab] OR "Lutembacher*"[tiab] OR "Patent Ostium Secundum"[tiab] OR "Persistent Ostium Secundum"[tiab] OR "Secundum Atrial Defect*"[tiab]) OR ("Heart Septal Defects, Ventricular"[Mesh] OR "Ventricular Septal Defect*"[tiab] OR "Intraventricular Septal Defect*"[tiab] OR "Intraventricular Septum Defect*"[tiab] OR "Ventricle Septal Defect*"[tiab] OR "Ventricular Septum Defect*"[tiab] OR "Ventricle Septum Defect*"[tiab] OR "Interventricular Septal Defect*"[tiab] OR "Interventricular Septum Defect*"[tiab] OR "Ventricular Heart Septal Defect*"[tiab] OR "Ventricular Heart Septum Defect"[tiab:~0] OR "Ventricular Heart Septum Defects"[tiab:~0] OR "Ventricle Heart Septal Defect"[tiab:~0] OR "Ventricle Heart Septal Defects"[tiab:~0] OR "Ventricle Heart Septum Defect"[tiab:~0] OR "Ventricle Heart Septum Defects"[tiab:~0] OR "Ventricular Septal Perforation*"[tiab] OR "Ventricular Septum Perforation*"[tiab] OR "Ventricle Septal Perforation"[tiab:~0] OR "Ventricle Septal Perforations"[tiab:~0] OR "Ventricle Septum Perforation"[tiab:~0] OR "Ventricle Septum Perforations"[tiab:~0] OR "Interventricular Shunt*"[tiab] OR "Intraventricular Shunt*"[tiab] OR "Membranous Incomplete Septum"[tiab:~0] OR "Membranous Incomplete Septa"[tiab:~0]) OR ("Aortic Coarctation"[Mesh] OR "Aortic Coarctation*"[tiab] OR "Aorta Coarctation*"[tiab] OR "Coarctation of Aorta"[tiab] OR "Coarctation of the Aorta"[tiab] OR "Aorta Dominant Coarctation"[tiab:~0] OR "Aorta Dominant Coarctations"[tiab:~0] OR "Aortic Isthmus Stenosis"[tiab] OR "Coarctatio Aortae"[tiab]) OR ("Atrioventricular Septal Defect" [Supplementary Concept] OR "Atrioventricular Septal Defect*"[tiab] OR "Atrioventricular Canal Defect*"[tiab]) OR ("Double Outlet Right Ventricle"[Mesh] OR "Double Outlet Right Ventricle*"[tiab] OR "Taussig-Bing Anomal*"[tiab] OR "Double Outlet Right Heart Ventricle"[tiab:~0] OR "Right Ventricle Double Outlet"[tiab] OR "Right Ventricular Double Outlet"[tiab]) OR ("Ebstein Anomaly"[Mesh] OR "Ebstein*"[tiab]) OR ("Hypoplastic Left Heart Syndrome"[Mesh] OR "Left Heart Hypoplasia Syndrome"[tiab] OR "Hypoplastic Left Heart Syndrome"[tiab]) OR ("Aortic Arch Interruption, Facial Palsy, and Retinal Coloboma"[Supplementary Concept] OR "Aortic Arch Interruption*"[tiab] OR "Aorta Arch Interruption"[tiab:~0] OR "Aorta Arch Interruptions"[tiab:~0]) OR ("Ductus Arteriosus, Patent"[Mesh] OR "Patent Ductus Arterios*"[tiab] OR "Patency of the Ductus Arteriosus"[tiab] OR "Ductus Arteriosus Patency"[tiab] OR "Ductus Arteriosus Persisten*"[tiab] OR "Open Ductus Botalli"[tiab] OR "Patent Ductus Botalli"[tiab] OR "Persistent Ductus Arteriosus"[tiab] OR "Persistent Ductus Botalli"[tiab:~0] OR "Truncus Arteriosus Persistens"[tiab]) OR ("Pulmonary Valve Stenosis"[Mesh] OR "Pulmonic Stenos*"[tiab] OR "Pulmonary Stenos*"[tiab] OR "Pulmonary Valve Stenos*"[tiab] OR "Pulmonary Valva Stenosis"[tiab:~0] OR "Pulmonary Valva Stenoses"[tiab:~0] OR "Pulmonal Stenos*"[tiab] OR "Lung Artery Valve Stenosis"[tiab:~0] OR "Lung Artery Valve Stenoses"[tiab:~0] OR "Lung Artery Valvular Stenosis"[tiab:~0] OR "Lung Artery Valvular Stenoses"[tiab:~0] OR "Lung Valve Stenosis"[tiab:~0] OR "Lung Valve Stenoses"[tiab:~0]) OR ("Pulmonary Atresia"[Mesh] OR

“Pulmonary Atresia*”[tiab] OR “Pulmonary Valve Atresia*”[tiab] OR “Pulmonic Atresia*”[tiab] OR “Pulmonic Valve Atresia*”[tiab] OR “Pulmonary Artery Atresia*”[tiab] OR “Pulmonic Artery Atresia”[tiab:~0] OR “Pulmonic Artery Atresias”[tiab:~0] OR “Lung Atresia*”[tiab] OR “Lung Artery Atresia”[tiab:~0] OR “Lung Artery Atresias”[tiab:~0] OR “Lung Valve Atresia”[tiab:~0] OR “Lung Valve Atresias”[tiab:~0] OR “Lung Artery Valve Atresia”[tiab:~0] OR “Lung Artery Valve Atresias”[tiab:~0]) OR ("Univentricular Heart"[Mesh] OR “Univentricular Heart*”[tiab] OR “Complex Single Ventricle*”[tiab] OR “Single Heart Ventricle*”[tiab] OR “Heart Single Ventricle*”[tiab] OR “Monoventricular Heart*”[tiab] OR “Cor Monoventriculare”[tiab:~0] OR “Cor Triloculare Bia*”[tiab]) OR ("Tetralogy of Fallot"[Mesh] OR “Fallot*”[tiab]) OR ("Scimitar Syndrome"[Mesh] OR “Scimitar Syndrome”[tiab] OR “Scimitar Anomaly”[tiab] OR “Anomalous Pulmonary Venous Return”[tiab] OR “Pulmonary Venous Return Anomaly”[tiab] OR “Anomalous Lung Vein Drainage”[tiab:~0] OR “Anomalous Pulmonary Vein Drainage”[tiab] OR “Anomalous Pulmonary Venous”[tiab] OR “Lung Venous Drainage Anomaly”[tiab:~0] OR “Lung Vein Drainage Anomaly”[tiab:~0] OR “Lung Venous Return Anomaly”[tiab:~0] OR “Pulmonary Venous Drainage Anomaly”[tiab:~0]) OR ("Transposition of Great Vessels"[Mesh] OR “Transposition of Great Arter*”[tiab] OR “Transposition of Great Vessel*”[tiab] OR "Great Vessels Transposition*”[tiab] OR "Great Vessel Transposition*”[tiab] OR "Great Arteries Transposition*”[tiab] OR "Great Artery Transposition*”[tiab] OR "Great Vessels Dextrotransposition”[tiab:~0] OR "Great Vessels Dextrotranspositions”[tiab:~0] OR “Great Vessels Levotransposition”[tiab:~0] OR “Great Vessels Levotranspositions”[tiab:~0] OR "Levotransposition of Great Vessel”[tiab:~0] OR "Levotransposition of Great Vessesl”[tiab:~0] OR "Dextrotransposition of Great Vessel*”[tiab] OR "Dextro-Looped Transposition of the Great Arter*”[tiab] OR "Dextro Looped Transposition of the Great Arter*”[tiab] OR "Levo-Looped Transposition of the Great Arter*”[tiab] OR "Large Vessel Transposition*”[tiab]) OR ("Tricuspid Atresia"[Mesh] OR "Tricuspid Atresia*”[tiab] OR “Tricuspid Valve Atresia*”[tiab] OR “Absent Right Atrioventricular Connection*”[tiab] OR "Right Atrioventricular Cardiac Valve Atresia”[tiab:~0] OR "Right Atrioventricular Cardiac Valve Atresias”[tiab:~0] OR "Right Atrioventricular Cardiac Valvular Atresia”[tiab:~0] OR "Right Atrioventricular Cardiac Valvular Atresias”[tiab:~0] OR "Right Atrioventricular Heart Valve Atresia”[tiab:~0] OR "Right Atrioventricular Heart Valve Atresias”[tiab:~0] OR "Right Atrioventricular Heart Valvular Atresia”[tiab:~0] OR "Right Atrioventricular Heart Valvular Atresias”[tiab:~0] OR "Right Atrioventricular Valve Atresia*”[tiab] OR "Right Atrioventricular Valvular Atresia”[tiab:~0] OR "Right Atrioventricular Valvular Atresias”[tiab:~0] OR "Tricuspid Cardiac Valve Atresia”[tiab:~0] OR "Tricuspid Cardiac Valve Atresias”[tiab:~0] OR "Tricuspid Heart Valve Atresia”[tiab:~0] OR "Tricuspid Heart Valve Atresias”[tiab:~0] OR "Tricuspid Valvular Atresia*”[tiab]) OR ("Truncus Arteriosus"[Mesh] OR "Arterial Trun*”[tiab] OR "Truncus Arteri*”[tiab]) OR ("Aortic Valve Stenosis"[Mesh] OR “Aortic Valve Stenos*”[tiab] OR “Aortic Stenos*”[tiab] OR “Aorta Valve Stenosis”[tiab:~0] OR “Aorta Valve Stenoses”[tiab:~0] OR “Aortic Valvular Stenos*”[tiab] OR “Stenosed Aortic Valve*”[tiab] OR “Stenotic Aortic Valve*”[tiab])

Concept 2:

("Infections"[Mesh:NoExp] OR “Infect*”[tiab] OR “Infest*”[tiab] OR “Inflammation*”[tiab] OR “TORCH*”[tiab]) OR ("COVID-19"[MeSH] OR nCoV[tiab] OR 2019nCoV[tiab] OR COVID[tiab] OR COVID19[tiab] OR SARS2[tiab] OR "cov 2"[tiab] OR cov2[tiab] OR coronavirus*[tiab] OR "corona virus*"[tiab] OR betacoronavirus*[tiab] OR "severe acute respiratory syndrome 2"[tiab:~0] OR "Wuhan virus"[tiab] OR nCoV[ad] OR 2019nCoV[ad] OR COVID[ad] OR COVID19[ad] OR "SARS-Cov-2"[MeSH] OR SARS2[ad] OR "severe acute respiratory syndrome 2"[ad:~0] OR "cov 2"[ad] OR cov2[ad] OR coronavirus*[ad] OR "corona virus*"[ad] OR betacoronavirus*[ad] OR ((wuhan[tiab] OR novel[tiab] OR new[tiab] OR 19[tiab] OR 2019[tiab] OR epidem*[tiab] OR pandem*[tiab] OR outbreak[tiab] OR wuhan[ad] OR novel[ad] OR new[ad] OR 19[ad] OR 2019[ad] OR epidemy[ad] OR epidemic*[ad] OR pandem*[ad] OR outbreak[ad]) AND ("Coronavirus"[Mesh:NoExp] OR "Betacoronavirus"[Mesh:NoExp] OR "Coronavirus Infections"[Mesh:NoExp] OR "pneumonia virus*"[tiab] OR cov[tiab] OR hcov[tiab]))) OR ("Rubella"[Mesh] OR "Rubella virus"[Mesh] OR “Rubella*”[tiab] OR “Three Day Measle*”[tiab] OR “German Measle*”[tiab] OR “Epidemic Roseola”[tiab:~0]) OR ("Hepatitis, Viral, Human"[Mesh] OR “Virus Hepatiti*”[tiab] OR “Viral Hepatiti*”[tiab] OR “Virus Liver Disease*”[tiab] OR “Viral Liver Disease*”[tiab] OR “Hepatitis Virus Infection*”[tiab] OR “Hepatitis Viral Infection*”[tiab] OR “Hepatitis due to Virus”[tiab:~0] OR “Hepatitis due to Viruses”[tiab:~0] OR “Hepatitis Caused by Virus”[tiab:~0] OR “Hepatitis Caused by Viruses”[tiab:~0]) OR ("Cytomegalovirus"[Mesh] OR “Cytomegalovirus*”[tiab] OR “Salivary Gland Virus*”[tiab] OR “Beta Herpes Virus 5”[tiab:~0] OR “Human Herpesvirus 5”[tiab] OR “Human Herpesvirus V”[tiab:~0] OR “Human Herpesvirus Type 5”[tiab] OR “Human Herpesvirus Type V”[tiab:~0] OR “Herpes Simplex Virus 5”[tiab:~0] OR “Herpes Simplex Virus V”[tiab:~0] OR “Herpes Simplex Virus Type 5”[tiab:~0] OR “Herpes Simplex Virus Type V”[tiab:~0] OR “Herpes Virus 5”[tiab] OR “Herpes Virus V”[tiab:~0] OR “Herpes Virus Type 5”[tiab:~0] OR “Herpes Virus Type

V”[tiab:~0]) OR ("Herpesvirus 1, Human"[Mesh] OR “Human Alphaherpesvirus 1”[tiab] OR “Human Herpesvirus 1”[tiab] OR “Human Herpesvirus I”[tiab] OR “Human Herpesvirus Type 1”[tiab] OR “Human Herpesvirus Type I”[tiab:~0] OR “Herpes Simplex Virus 1”[tiab] OR “Herpes Simplex Virus I”[tiab] OR “Herpes Simplex Virus Type 1”[tiab] OR “Herpes Simplex Virus Type I”[tiab] OR “Herpes Virus 1”[tiab] OR “Herpes Virus I”[tiab] OR “Herpes Virus Type 1”[tiab] OR “Herpes Virus Type I”[tiab]) OR ("Herpesvirus 2, Human"[Mesh] OR “Human Herpesvirus 2”[tiab] OR “Human Herpesvirus II”[tiab:~0] OR “Human Herpesvirus Type 2”[tiab] OR “Human Herpesvirus Type II”[tiab:~0] OR “Herpes Simplex Virus 2”[tiab] OR “Herpes Simplex Virus II”[tiab] OR “Herpes Simplex Virus Type 2”[tiab] OR “Herpes Simplex Virus Type II”[tiab] OR “Herpes Virus 2”[tiab] OR “Herpes Virus II”[tiab] OR “Herpes Virus Type 2”[tiab] OR “Herpes Virus Type II”[tiab]) OR ("Herpesvirus 4, Human"[Mesh] OR “Human Herpesvirus 4”[tiab] OR “Human Herpesvirus IV”[tiab:~0] OR “Human Herpesvirus Type 4”[tiab] OR “Human Herpesvirus Type IV”[tiab:~0] OR “Herpes Simplex Virus 4”[tiab:~0] OR “Herpes Simplex Virus IV”[tiab:~0] OR “Herpes Simplex Virus Type 4”[tiab:~0] OR “Herpes Simplex Virus Type IV”[tiab:~0] OR “Herpes Virus 4”[tiab] OR “Herpes Virus IV”[tiab:~0] OR “Herpes Virus Type 4”[tiab] OR “Herpes Virus Type IV”[tiab:~0] OR “Burkitt Herpesvirus”[tiab:~0] OR “Burkitt Lymphoma Virus”[tiab:~0] OR “Burkitt's Lymphoma Virus”[tiab:~0] OR “Burkitts Lymphoma Virus”[tiab:~0] OR “Infectious Mononucleosis Virus*”[tiab] OR “Infectious Mononucleosis Herpetovirus”[tiab:~0] OR “Mononucleosis Infectiosa Virus”[tiab:~0] OR “Epstein-Barr Virus*”[tiab] OR “Epstein Barr Virus*”[tiab] OR “Barr Epstein Virus”[tiab:~0] OR “Epstein Barr Herpetovirus”[tiab:~0] OR “Epstein Virus”[tiab]) OR ("Herpesvirus 3, Human"[Mesh] OR “Human Herpesvirus 3”[tiab] OR “Human Herpesvirus III”[tiab:~0] OR “Human Herpesvirus Type 3”[tiab:~0] OR “Human Herpesvirus Type III”[tiab:~0] OR “Herpes Simplex Virus 3”[tiab:~0] OR “Herpes Simplex Virus III”[tiab:~0] OR “Herpes Simplex Virus Type 3”[tiab:~0] OR “Herpes Simplex Virus Type III”[tiab:~0] OR “Herpes Virus 3”[tiab] OR “Herpes Virus III”[tiab:~0] OR “Herpes Virus Type 3”[tiab] OR “Herpes Virus Type III”[tiab:~0] OR “Chickenpox”[tiab] OR “Chicken Pox”[tiab] OR “Ocular Herpes Zoster Virus”[tiab:~0] OR “Shingles”[tiab] OR “Zona Virus”[tiab] OR “Varicella-Zoster Virus*”[tiab] OR “Varicella Zoster”[tiab] OR “Varicella Foster Virus”[tiab:~0] OR “Varicella Herpes Zoster Virus”[tiab] OR “Varicella Herpetovirus”[tiab:~0] OR “Varicella Pneumonia Virus”[tiab:~0] OR “Varicella Virus”[tiab] OR “Herpesvirus Varicellae”[tiab] OR “Herpes Zoster Virus*”[tiab] OR “Herpes Virus Varicellae”[tiab:~0] OR “Herpes Zoster Varicella Virus”[tiab:~0]) OR ("Lymphocytic choriomeningitis virus"[Mesh] OR “Chorio Meningitis”[tiab:~0] OR “Choriomeningitis ”[tiab]) OR ("Zika Virus"[Mesh] OR “Zika Virus*”[tiab] OR “Zika Flavivirus*”[tiab]) OR ("West Nile virus"[Mesh] OR "West Nile Virus*"[tiab] OR “Egypt 101 Virus*"[tiab] OR “Kunjin Virus*"[tiab] OR “West Nile Viral Disease"[tiab:~0] OR “West Nile Flavivirus Infection"[tiab:~0] OR “West Nile Infection*"[tiab] OR “West Nile Viral Infection*"[tiab]) OR ("Rift Valley fever virus"[Mesh] OR “Rift Valley Fever”[tiab] OR “Rift Valley Virus*”[tiab]) OR ("Parvovirus B19, Human"[Mesh] OR “B19 Virus*”[tiab] OR “Parvovirus B19*”[tiab]) OR ("HIV"[Mesh] OR “Human Immunodeficiency Virus*”[tiab] OR “Human T Cell Lymphotropic Virus Type III”[tiab] OR “Human T-Cell Leukemia Virus Type III”[tiab] OR “LAV-HTLV-III”[tiab] OR “Lymphadenopathy-Associated Virus*”[tiab] OR “Human T Lymphotropic Virus Type III”[tiab] OR “Acquired Immune Deficiency Syndrome ”[tiab] OR “Acquired Immunodeficiency Syndrome”[tiab] OR “Aids Associated Lentivirus”[tiab:~0] OR “Aids Associated Retrovirus”[tiab] OR “Aids Associated Virus”[tiab] OR “Aids Related Virus”[tiab] OR “Human Immuno Deficiency Virus”[tiab] OR “Immunodeficiency Associated Virus”[tiab:~0] OR “Lymphadenopathy Associated Retrovirus”[tiab]) OR ("Coxsackievirus Infections"[Mesh] OR “Coxsackievir*”[tiab] OR “Coxsackie Vir*”[tiab]) OR ("Listeria monocytogenes"[Mesh] OR “Listeria Monocytogenes”[tiab] OR “Listerella Hepatolytica”[tiab:~0] OR “Listeriosis Monocytogenes”[tiab:~0] OR “Bacterium Monocytogenes”[tiab:~0] OR “Corynebacterium Infantisepticum”[tiab] OR “Corynebacterium Parvulum”[tiab] OR “Erysipelothrix Monocytogenes”[tiab]) OR ("Treponema pallidum"[Mesh] OR "Treponema Pallid*”[tiab] OR "Treponema Reiterii”[tiab:~0] OR "Spirochaeta Pallida”[tiab]) OR ("Streptococcus agalactiae"[Mesh] OR "Streptococcus B”[tiab] OR "Streptococcus Group B”[tiab] OR "Group B Streptococcus”[tiab] OR "Staphylococcus Agalactiae”[tiab] OR "Streptococcus Mastitidis”[tiab] OR "Streptococcus Nocardi”[tiab:~0] OR "Streptococcus Agalactiae”[tiab]) OR ("Staphylococcus aureus"[Mesh] OR "Staphylococcus Aureus”[tiab] OR "Staphylococcus Pyogenes aureus”[tiab] OR "Staphylococcus Pyogenes Citreus”[tiab:~0] OR "Micrococcus Aureus”[tiab] OR "Micrococcus Pyogenes”[tiab]) OR ("Escherichia coli"[Mesh] OR “Enterococcus Coli”[tiab] OR “Escherichia Coli”[tiab] OR "Bacillus Coli”[tiab] OR "Bacterium Coli”[tiab] OR "Alkalescens-Dispar Group”[tiab] OR "EAggEC”[tiab] OR "Bacillus Escherichii”[tiab:~0] OR "Bacterium E3”[tiab:~0] OR "Coli Bacillus”[tiab] OR "Coli Bacterium”[tiab] OR "Colibacillus”[tiab] OR "Colon Bacillus”[tiab] OR "Escherichia Alkalescens Dispart”[tiab:~0]) OR ("Chlamydia trachomatis"[Mesh] OR “Chlamydia Trachomatis”[tiab] OR "Chlamidia Trachomatis”[tiab] OR "Chlamydophila Trachomatis”[tiab:~0] OR "Chlamydozoon Trachomatis”[tiab] OR "Rickettsia Trachomae”[tiab:~0] OR "Rickettsia Trachomatis”[tiab:~0]) OR ("Helicobacter pylori"[Mesh] OR “Helicobacter Pylori”[tiab] OR "Helicobacter Nemestrinae”[tiab] OR "Campylobacter Pylori”[tiab]) OR ("Plasmodium falciparum"[Mesh] OR “Plasmodium Falciparum*”[tiab]) OR ("Plasmodium vivax"[Mesh] OR “Plasmodium Vivax*”[tiab]) OR ("Toxoplasma"[Mesh] OR “Toxoplasma*”[tiab]

OR "Toxoplasma Gondi*"[tiab] OR "Toxoplasma Hominis"[tiab]) OR ("Trypanosoma cruzi"[Mesh] OR "Trypanosoma cruz*"[tiab] OR "Schizotrypanum Cruzi"[tiab])

Concept 3:

"Maternal Exposure"[Mesh] OR "Prenatal Exposure Delayed Effects"[Mesh] OR "Maternal-Fetal Exchange"[Mesh] OR "Maternal"[tiab] OR "Transplacental"[tiab] OR "Prenatal"[tiab] OR "Mother*"[tiab] OR "Pregnan*"[tiab] OR "Women"[tiab] OR "Woman"[tiab] OR "Female"[tiab] OR "In Utero"[tiab] OR "Uteroplacental"[tiab]

Web of Science: Web of Science Core Collection

Editions:

(1) Science Citation Index Expanded: (SCI-EXPANDED)--1955-present

(2) Social Sciences Citation Index: (SSCI)--1956-present

(3) Arts & Humanities Citation Index: (AHCI)--1975-present

(4) Conference Proceedings Citation Index – Science: (CPCI-S)--1990-present

(5) Conference Proceedings Citation Index – Social Science & Humanities: (CPCI-SSH)--1990-present

(6) Emerging Sources Citation Index: (ESCI)--2019-present

(Concept 1) AND (Concept 2) AND (Concept 3) NOT DT=("meeting abstract")

Concept 1:

TS=(("Congenital Heart Defect*" OR "Congenital Heart Disease*" OR "Congenital Cardiac Disease*" OR "Congenital Cardiac Distress" OR "Congenital Cardiac Distresses" OR "Congenital Heart Distress" OR "Congenital Heart Distresses" OR "Congenital Heart Failure" OR "Heart Congenital Disease*" OR "Heart Congenital Defect*" OR "Heart Congenital Anomaly" OR "Heart Congenital Anomalies" OR "Heart Congenital Malformation*" OR "Neonatal Cardiopathy" OR "Congenital Heart Malformation*" OR "Congenital Malformation of the Heart" OR "Congenital Malformations of the Heart" OR "Congenital Heart Anomal*" OR "Congenital Cardiac Defect*" OR "Congenital Cardiac Malformation*" OR "Congenital Cardiac Anomal*" OR "Congenital Cardiovascular Disease*") OR ("Atrial Septal Defect*" OR "Atrium septum defect*" OR "Persistent Ostium Primum" OR "Atrial Heart Septal Defect*" OR "Atrial Heart Shunt" OR "Atrial Septum Defect*" OR "Atrium Heart Septal Defect" OR "Atrium Heart Septal Defects" OR "Atrial Heart Septum Defect" OR "Atrial Heart Septum Defects" OR "Atrium Heart Septum Defect" OR "Atrium Heart Septum Defects" OR "Atrium Septal Defect*" OR "Atrium Septum Primum Defect" OR "Atrium Septum Primum Defects" OR "Atrial Septum Primum Defect" OR "Atrial Septum Primum Defects" OR "Atrium Septum Secundum Defect" OR "Atrium Septum Secundum Defects" OR "Atrial Septum Secundum Defect" OR "Atrial Septum Secundum Defects" OR "Cleft Heart Atrium" OR "Interatrial Septal Defect*" OR "Interatrial Septum Defect*" OR "Interauricular Septal Defect*" OR "Lutembacher*" OR "Patent Ostium Secundum" OR "Persistent Ostium Secundum" OR "Secundum Atrial Defect*") OR ("Ventricular Septal Defect*" OR "Intraventricular Septal Defect*" OR "Intraventricular Septum Defect*" OR "Ventricle Septal Defect*" OR "Ventricular Septum Defect*" OR "Ventricle Septum Defect*" OR "Interventricular Septal Defect*" OR "Interventricular Septum Defect*" OR "Ventricular Heart Septal Defect*" OR "Ventricular Heart Septum Defect" OR "Ventricular Heart Septum Defects" OR "Ventricle Heart Septal Defect" OR "Ventricle Heart Septal Defects" OR "Ventricle Heart Septum Defect" OR "Ventricle Heart Septum Defects" OR "Ventricular Septal Perforation*" OR "Ventricular Septum Perforation*" OR "Ventricle Septal Perforation" OR "Ventricle Septal Perforations" OR "Ventricle Septum Perforation" OR "Ventricle Septum Perforations" OR "Interventricular Shunt*" OR "Intraventricular Shunt*" OR "Membranous Incomplete Septum" OR "Membranous Incomplete Septa") OR ("Aortic Coarctation*" OR "Aorta Coarctation*" OR "Coarctation of Aorta" OR "Coarctation of the Aorta" OR "Aorta Dominant Coarctation" OR "Aorta Dominant Coarctations" OR "Aortic Isthmus Stenosis" OR "Coarctatio Aortae") OR ("Atrioventricular Septal Defect*" OR "Atrioventricular Canal Defect*") OR ("Double

Outlet Right Ventricle*” OR “Taussig-Bing Anomal*” OR “Double Outlet Right Heart Ventricle” OR “Right Ventricle Double Outlet” OR “Right Ventricular Double Outlet”) OR (“Ebstein*”) OR ("Left Heart Hypoplasia Syndrome” OR “Hypoplastic Left Heart Syndrome”) OR ("Aortic Arch Interruption*” OR “Aorta Arch Interruption” OR “Aorta Arch Interruptions”) OR (“Patent Ductus Arterios*” OR “Patency of the Ductus Arteriosus” OR “Ductus Arteriosus Patency” OR “Ductus Arteriosus Persisten*” OR “Open Ductus Botalli” OR “Patent Ductus Botalli” OR “Persistent Ductus Arteriosus” OR “Persistent Ductus Botalli” OR “Truncus Arteriosus Persistens”) OR (“Pulmonic Stenos*” OR “Pulmonary Stenos*” OR “Pulmonary Valve Stenos*” OR “Pulmonary Valva Stenosis” OR “Pulmonary Valva Stenoses” OR “Pulmonal Stenos*” OR “Lung Artery Valve Stenosis” OR “Lung Artery Valve Stenoses” OR “Lung Artery Valvular Stenosis” OR “Lung Artery Valvular Stenoses” OR “Lung Valve Stenosis” OR “Lung Valve Stenoses”) OR (“Pulmonary Atresia*” OR “Pulmonary Valve Atresia*” OR “Pulmonic Atresia*” OR “Pulmonic Valve Atresia*” OR “Pulmonary Artery Atresia*” OR “Pulmonic Artery Atresia” OR “Pulmonic Artery Atresias” OR “Lung Atresia*” OR “Lung Artery Atresia” OR “Lung Artery Atresias” OR “Lung Valve Atresia” OR “Lung Valve Atresias” OR “Lung Artery Valve Atresia” OR “Lung Artery Valve Atresias”) OR (“Univentricular Heart*” OR “Complex Single Ventricle*” OR “Single Heart Ventricle*” OR “Heart Single Ventricle*” OR “Monoventricular Heart*” OR “Cor Monoventriculare” OR “Cor Triloculare Bia*”) OR (“Fallot*”) OR (“Scimitar Syndrome” OR “Scimitar Anomaly” OR “Anomalous Pulmonary Venous Return” OR “Pulmonary Venous Return Anomaly” OR “Anomalous Lung Vein Drainage” OR “Anomalous Pulmonary Vein Drainage” OR “Anomalous Pulmonary Venous” OR “Lung Venous Drainage Anomaly” OR “Lung Vein Drainage Anomaly” OR “Lung Venous Return Anomaly” OR “Pulmonary Venous Drainage Anomaly”) OR ("Transposition of Great Arter*” OR “Transposition of Great Vessel*” OR "Great Vessels Transposition*” OR "Great Vessel Transposition*” OR "Great Arteries Transposition*” OR "Great Artery Transposition*” OR "Great Vessels Dextrotransposition” OR "Great Vessels Dextrotranspositions” OR “Great Vessels Levotransposition” OR “Great Vessels Levotranspositions” OR "Levotransposition of Great Vessel” OR "Levotransposition of Great Vessesl” OR "Dextrotransposition of Great Vessel*” OR "Dextro-Looped Transposition of the Great Arter*” OR "Dextro Looped Transposition of the Great Arter*” OR "Levo-Looped Transposition of the Great Arter*” OR "Large Vessel Transposition*”) OR ("Tricuspid Atresia*” OR “Tricuspid Valve Atresia*” OR “Absent Right Atrioventricular Connection*” OR "Right Atrioventricular Cardiac Valve Atresia” OR "Right Atrioventricular Cardiac Valve Atresias” OR "Right Atrioventricular Cardiac Valvular Atresia” OR "Right Atrioventricular Cardiac Valvular Atresias” OR "Right Atrioventricular Heart Valve Atresia” OR "Right Atrioventricular Heart Valve Atresias” OR "Right Atrioventricular Heart Valvular Atresia” OR "Right Atrioventricular Heart Valvular Atresias” OR "Right Atrioventricular Valve Atresia*” OR "Right Atrioventricular Valvular Atresia” OR "Right Atrioventricular Valvular Atresias” OR "Tricuspid Cardiac Valve Atresia” OR "Tricuspid Cardiac Valve Atresias” OR "Tricuspid Heart Valve Atresia” OR "Tricuspid Heart Valve Atresias” OR "Tricuspid Valvular Atresia*”) OR ("Arterial Trun*” OR "Truncus Arteri*”) OR (“Aortic Valve Stenos*” OR “Aortic Stenos*” OR “Aorta Valve Stenosis” OR “Aorta Valve Stenoses” OR “Aortic Valvular Stenos*” OR “Stenosed Aortic Valve*” OR “Stenotic Aortic Valve*”))

Concept 2:

TS=((“Infect*” OR “Infest*” OR “Inflammation*” OR “TORCH*”) OR (“nCoV” OR “2019nCoV” OR “COVID” OR “COVID19” OR “SARS2” OR “cov 2” OR “cov2” OR “coronavirus*” OR “corona virus*” OR "betacoronavirus*" OR "severe acute respiratory syndrome 2" OR “wuhan virus” OR ((“wuhan” OR “novel” OR “19” OR “2019” OR “epidem*” OR “pandem*” OR “outbreak” OR “new”) AND (”pneumonia virus*” OR “cov” OR “hcov”))) OR (“Rubella*” OR “Three Day Measle*” OR “German Measle*” OR “Epidemic Roseola”) OR (“Virus Hepatiti*” OR “Viral Hepatiti*” OR “Virus Liver Disease*” OR “Viral Liver Disease*” OR “Hepatitis Virus Infection*” OR “Hepatitis Viral Infection*” OR “Hepatitis due to Virus*” OR “Hepatitis Caused by Virus*”) OR (“Cytomegalovirus*” OR “Salivary Gland Virus*” OR “Beta Herpes Virus 5” OR “Human Herpesvirus 5” OR “Human Herpesvirus V” OR “Human Herpesvirus Type 5” OR “Human Herpesvirus Type V” OR “Herpes Simplex Virus 5” OR “Herpes Simplex Virus V” OR “Herpes Simplex Virus Type 5” OR “Herpes Simplex Virus Type V” OR “Herpes Virus 5” OR “Herpes Virus V” OR “Herpes Virus Type 5” OR “Herpes Virus Type V”) OR (“Human Alphaherpesvirus 1” OR “Human Herpesvirus 1” OR “Human Herpesvirus I” OR “Human Herpesvirus Type 1” OR “Human Herpesvirus Type I” OR “Herpes Simplex Virus 1” OR “Herpes Simplex Virus I” OR “Herpes Simplex Virus Type 1” OR “Herpes Simplex Virus Type I” OR “Herpes Virus 1” OR “Herpes Virus I” OR “Herpes Virus Type 1” OR “Herpes Virus Type I”) OR (“Human Herpesvirus 2” OR “Human Herpesvirus II” OR “Human Herpesvirus Type 2” OR “Human Herpesvirus Type II” OR “Herpes Simplex Virus 2” OR “Herpes Simplex Virus II” OR “Herpes Simplex Virus Type 2” OR “Herpes Simplex Virus Type II” OR “Herpes Virus 2” OR “Herpes Virus II” OR “Herpes Virus Type 2” OR “Herpes Virus Type II”) OR (“Human Herpesvirus 4” OR “Human Herpesvirus IV” OR “Human Herpesvirus

Type 4” OR “Human Herpesvirus Type IV” OR “Herpes Simplex Virus 4” OR “Herpes Simplex Virus IV” OR “Herpes Simplex Virus Type 4” OR “Herpes Simplex Virus Type IV” OR “Herpes Virus 4” OR “Herpes Virus IV” OR “Herpes Virus Type 4” OR “Herpes Virus Type IV” OR “Burkitt Herpesvirus” OR “Burkitt Lymphoma Virus” OR “Burkitt's Lymphoma Virus” OR “Burkitts Lymphoma Virus” OR “Infectious Mononucleosis Virus*” OR “Infectious Mononucleosis Herpetovirus” OR “Mononucleosis Infectiosa Virus” OR “Epstein-Barr Virus*” OR “Epstein Barr Virus*” OR “Barr Epstein Virus” OR “Epstein Barr Herpetovirus” OR “Epstein Virus”) OR (“Human Herpesvirus 3” OR “Human Herpesvirus III” OR “Human Herpesvirus Type 3” OR “Human Herpesvirus Type III” OR “Herpes Simplex Virus 3” OR “Herpes Simplex Virus III” OR “Herpes Simplex Virus Type 3” OR “Herpes Simplex Virus Type III” OR “Herpes Virus 3” OR “Herpes Virus III” OR “Herpes Virus Type 3” OR “Herpes Virus Type III” OR “Chickenpox” OR “Chicken Pox” OR “Ocular Herpes Zoster Virus” OR “Shingles” OR “Zona Virus” OR “Varicella-Zoster Virus*” OR “Varicella Zoster” OR “Varicella Foster Virus” OR “Varicella Herpes Zoster Virus” OR “Varicella Herpetovirus” OR “Varicella Pneumonia Virus” OR “Varicella Virus” OR “Herpesvirus Varicellae” OR “Herpes Zoster Virus*” OR “Herpes Virus Varicellae” OR “Herpes Zoster Varicella Virus”) OR (“Chorio Meningitis” OR “Choriomeningitis “) OR (“Zika Virus*” OR “Zika Flavivirus*”) OR ("West Nile Virus*” OR “Egypt 101 Virus*” OR “Kunjin Virus*” OR “West Nile Viral Disease” OR “West Nile Flavivirus Infection” OR “West Nile Infection*” OR “West Nile Viral Infection*”) OR (“Rift Valley Fever” OR “Rift Valley Virus*”) OR (“B19 Virus*” OR “Parvovirus B19*”) OR (“Human Immunodeficiency Virus*” OR “Human T Cell Lymphotropic Virus Type III” OR “Human T-Cell Leukemia Virus Type III” OR “LAV-HTLV-III” OR “Lymphadenopathy-Associated Virus*” OR “Human T Lymphotropic Virus Type III” OR “Acquired Immune Deficiency Syndrome “ OR “Acquired Immunodeficiency Syndrome” OR “Aids Associated Lentivirus” OR “Aids Associated Retrovirus” OR “Aids Associated Virus” OR “Aids Related Virus” OR “Human Immuno Deficiency Virus” OR “Immunodeficiency Associated Virus” OR “Lymphadenopathy Associated Retrovirus”) OR (“Coxsackievir*” OR “Coxsackie Vir*”) OR (“Listeria Monocytogenes” OR “Listerella Hepatolytica” OR “Listeriosis Monocytogenes” OR “Bacterium Monocytogenes” OR “Corynebacterium Infantisepticum” OR “Corynebacterium Parvulum” OR “Erysipelothrix Monocytogenes”) OR ("Treponema Pallid*” OR "Treponema Reiterii” OR "Spirochaeta Pallida”) OR ("Streptococcus B” OR "Streptococcus Group B” OR "Group B Streptococcus” OR "Staphylococcus Agalactiae” OR "Streptococcus Mastitidis” OR "Streptococcus Nocardi” OR "Streptococcus Agalactiae”) OR ("Staphylococcus Aureus” OR "Staphylococcus Pyogenes aureus” OR "Staphylococcus Pyogenes Citreus” OR "Micrococcus Aureus” OR "Micrococcus Pyogenes”) OR (“Enterococcus Coli” OR “Escherichia Coli” OR "Bacillus Coli” OR "Bacterium Coli” OR "Alkalescens-Dispar Group” OR "EAggEC” OR "Bacillus Escherichii” OR "Bacterium E3” OR "Coli Bacillus” OR "Coli Bacterium” OR "Colibacillus” OR "Colon Bacillus” OR "Escherichia Alkalescens Dispart”) OR (“Chlamydia Trachomatis” OR "Chlamidia Trachomatis” OR "Chlamydophila Trachomatis” OR "Chlamydozoon Trachomatis” OR "Rickettsia Trachomae” OR "Rickettsia Trachomatis”) OR (“Helicobacter Pylori” OR "Helicobacter Nemestrinae” OR "Campylobacter Pylori”) OR (“Plasmodium Falciparum*”) OR (“Plasmodium Vivax*”) OR (“Toxoplasma*” OR "Toxoplasma Gondi*” OR "Toxoplasma Hominis”) OR ("Trypanosoma cruz*” OR “Schizotrypanum Cruzi”))

Concept 3:

TS=(“Maternal” OR "Transplacental” OR “Prenatal” OR “Mother*” OR “Pregnan*” OR “Women” OR “Woman” OR “Female” OR “In Utero” OR “Uteroplacental”)

Scopus: Scopus

(Concept 1) AND (Concept 2) AND (Concept 3)

Concept 1:

TITLE-ABS(("Congenital Heart Defect*” OR "Congenital Heart Disease*” OR "Congenital Cardiac Disease*” OR "Congenital Cardiac Distress” OR "Congenital Cardiac Distresses” OR "Congenital Heart Distress” OR "Congenital Heart Distresses” OR "Congenital Heart Failure” OR "Heart Congenital Disease*” OR “Heart Congenital Defect*” OR “Heart Congenital Anomaly” OR “Heart Congenital Anomalies” OR “Heart Congenital Malformation*” OR "Neonatal Cardiopathy” OR “Congenital Heart Malformation*” OR “Congenital Malformation of the Heart” OR “Congenital Malformations of the Heart” OR “Congenital Heart Anomal*” OR “Congenital Cardiac Defect*” OR “Congenital Cardiac Malformation*” OR “Congenital Cardiac Anomal*” OR “Congenital Cardiovascular

Disease*") OR ("Atrial Septal Defect*" OR "Atrium septum defect*" OR "Persistent Ostium Primum" OR "Atrial Heart Septal Defect*" OR "Atrial Heart Shunt" OR "Atrial Septum Defect*" OR "Atrium Heart Septal Defect" OR "Atrium Heart Septal Defects" OR "Atrial Heart Septum Defect" OR "Atrial Heart Septum Defects" OR "Atrium Heart Septum Defect" OR "Atrium Heart Septum Defects" OR "Atrium Septal Defect*" OR "Atrium Septum Primum Defect" OR "Atrium Septum Primum Defects" OR "Atrial Septum Primum Defect" OR "Atrial Septum Primum Defects" OR "Atrium Septum Secundum Defect" OR "Atrium Septum Secundum Defects" OR "Atrial Septum Secundum Defect" OR "Atrial Septum Secundum Defects" OR "Cleft Heart Atrium" OR "Interatrial Septal Defect*" OR "Interatrial Septum Defect*" OR "Interauricular Septal Defect*" OR "Lutembacher*" OR "Patent Ostium Secundum" OR "Persistent Ostium Secundum" OR "Secundum Atrial Defect*") OR ("Ventricular Septal Defect*" OR "Intraventricular Septal Defect*" OR "Intraventricular Septum Defect*" OR "Ventricle Septal Defect*" OR "Ventricular Septum Defect*" OR "Ventricle Septum Defect*" OR "Interventricular Septal Defect*" OR "Interventricular Septum Defect*" OR "Ventricular Heart Septal Defect*" OR "Ventricular Heart Septum Defect" OR "Ventricular Heart Septum Defects" OR "Ventricle Heart Septal Defect" OR "Ventricle Heart Septal Defects" OR "Ventricle Heart Septum Defect" OR "Ventricle Heart Septum Defects" OR "Ventricular Septal Perforation*" OR "Ventricular Septum Perforation*" OR "Ventricle Septal Perforation" OR "Ventricle Septal Perforations" OR "Ventricle Septum Perforation" OR "Ventricle Septum Perforations" OR "Interventricular Shunt*" OR "Intraventricular Shunt*" OR "Membranous Incomplete Septum" OR "Membranous Incomplete Septa") OR ("Aortic Coarctation*" OR "Aorta Coarctation*" OR "Coarctation of Aorta" OR "Coarctation of the Aorta" OR "Aorta Dominant Coarctation" OR "Aorta Dominant Coarctations" OR "Aortic Isthmus Stenosis" OR "Coarctatio Aortae") OR ("Atrioventricular Septal Defect*" OR "Atrioventricular Canal Defect*") OR ("Double Outlet Right Ventricle*" OR "Taussig-Bing Anomal*" OR "Double Outlet Right Heart Ventricle" OR "Right Ventricle Double Outlet" OR "Right Ventricular Double Outlet") OR ("Ebstein*") OR ("Left Heart Hypoplasia Syndrome" OR "Hypoplastic Left Heart Syndrome") OR ("Aortic Arch Interruption*" OR "Aorta Arch Interruption" OR "Aorta Arch Interruptions") OR ("Patent Ductus Arterios*" OR "Patency of the Ductus Arteriosus" OR "Ductus Arteriosus Patency" OR "Ductus Arteriosus Persisten*" OR "Open Ductus Botalli" OR "Patent Ductus Botalli" OR "Persistent Ductus Arteriosus" OR "Persistent Ductus Botalli" OR "Truncus Arteriosus Persistens") OR ("Pulmonic Stenos*" OR "Pulmonary Stenos*" OR "Pulmonary Valve Stenos*" OR "Pulmonary Valva Stenosis" OR "Pulmonary Valva Stenoses" OR "Pulmonal Stenos*" OR "Lung Artery Valve Stenosis" OR "Lung Artery Valve Stenoses" OR "Lung Artery Valvular Stenosis" OR "Lung Artery Valvular Stenoses" OR "Lung Valve Stenosis" OR "Lung Valve Stenoses") OR ("Pulmonary Atresia*" OR "Pulmonary Valve Atresia*" OR "Pulmonic Atresia*" OR "Pulmonic Valve Atresia*" OR "Pulmonary Artery Atresia*" OR "Pulmonic Artery Atresia" OR "Pulmonic Artery Atresias" OR "Lung Atresia*" OR "Lung Artery Atresia" OR "Lung Artery Atresias" OR "Lung Valve Atresia" OR "Lung Valve Atresias" OR "Lung Artery Valve Atresia" OR "Lung Artery Valve Atresias") OR ("Univentricular Heart*" OR "Complex Single Ventricle*" OR "Single Heart Ventricle*" OR "Heart Single Ventricle*" OR "Monoventricular Heart*" OR "Cor Monoventriculare" OR "Cor Triloculare Bia*") OR ("Fallot*") OR ("Scimitar Syndrome" OR "Scimitar Anomaly" OR "Anomalous Pulmonary Venous Return" OR "Pulmonary Venous Return Anomaly" OR "Anomalous Lung Vein Drainage" OR "Anomalous Pulmonary Vein Drainage" OR "Anomalous Pulmonary Venous" OR "Lung Venous Drainage Anomaly" OR "Lung Vein Drainage Anomaly" OR "Lung Venous Return Anomaly" OR "Pulmonary Venous Drainage Anomaly") OR ("Transposition of Great Arter*" OR "Transposition of Great Vessel*" OR "Great Vessels Transposition*" OR "Great Vessel Transposition*" OR "Great Arteries Transposition*" OR "Great Artery Transposition*" OR "Great Vessels Dextrotransposition" OR "Great Vessels Dextrotranspositions" OR "Great Vessels Levotransposition" OR "Great Vessels Levotranspositions" OR "Levotransposition of Great Vessel" OR "Levotransposition of Great Vessesl" OR "Dextrotransposition of Great Vessel*" OR "Dextro-Looped Transposition of the Great Arter*" OR "Dextro Looped Transposition of the Great Arter*" OR "Levo-Looped Transposition of the Great Arter*" OR "Large Vessel Transposition*") OR ("Tricuspid Atresia*" OR "Tricuspid Valve Atresia*" OR "Absent Right Atrioventricular Connection*" OR "Right Atrioventricular Cardiac Valve Atresia" OR "Right Atrioventricular Cardiac Valve Atresias" OR "Right Atrioventricular Cardiac Valvular Atresia" OR "Right Atrioventricular Cardiac Valvular Atresias" OR "Right Atrioventricular Heart Valve Atresia" OR "Right Atrioventricular Heart Valve Atresias" OR "Right Atrioventricular Heart Valvular Atresia" OR "Right Atrioventricular Heart Valvular Atresias" OR "Right Atrioventricular Valve Atresia*" OR "Right Atrioventricular Valvular Atresia" OR "Right Atrioventricular Valvular Atresias" OR "Tricuspid Cardiac Valve Atresia" OR "Tricuspid Cardiac Valve Atresias" OR "Tricuspid Heart Valve Atresia" OR "Tricuspid Heart Valve Atresias" OR "Tricuspid Valvular Atresia*") OR ("Arterial Trun*" OR "Truncus Arteri*") OR ("Aortic Valve Stenos*" OR "Aortic Stenos*" OR "Aorta Valve Stenosis" OR "Aorta Valve Stenoses" OR "Aortic Valvular Stenos*" OR "Stenosed Aortic Valve*" OR "Stenotic Aortic Valve*")) OR AUTHKEY (("Congenital Heart Defect*" OR "Congenital Heart Disease*" OR "Congenital Cardiac Disease*" OR "Congenital Cardiac Distress" OR "Congenital Cardiac Distresses" OR "Congenital Heart Distress" OR "Congenital Heart Distresses" OR "Congenital

Heart Failure" OR "Heart Congenital Disease*" OR "Heart Congenital Defect*" OR "Heart Congenital Anomaly" OR "Heart Congenital Anomalies" OR "Heart Congenital Malformation*" OR "Neonatal Cardiopathy" OR "Congenital Heart Malformation*" OR "Congenital Malformation of the Heart" OR "Congenital Malformations of the Heart" OR "Congenital Heart Anomal*" OR "Congenital Cardiac Defect*" OR "Congenital Cardiac Malformation*" OR "Congenital Cardiac Anomal*" OR "Congenital Cardiovascular Disease*") OR ("Atrial Septal Defect*" OR "Atrium septum defect*" OR "Persistent Ostium Primum" OR "Atrial Heart Septal Defect*" OR "Atrial Heart Shunt" OR "Atrial Septum Defect*" OR "Atrium Heart Septal Defect" OR "Atrium Heart Septal Defects" OR "Atrial Heart Septum Defect" OR "Atrial Heart Septum Defects" OR "Atrium Heart Septum Defect" OR "Atrium Heart Septum Defects" OR "Atrium Septal Defect*" OR "Atrium Septum Primum Defect" OR "Atrium Septum Primum Defects" OR "Atrial Septum Primum Defect" OR "Atrial Septum Primum Defects" OR "Atrium Septum Secundum Defect" OR "Atrium Septum Secundum Defects" OR "Atrial Septum Secundum Defect" OR "Atrial Septum Secundum Defects" OR "Cleft Heart Atrium" OR "Interatrial Septal Defect*" OR "Interatrial Septum Defect*" OR "Interauricular Septal Defect*" OR "Lutembacher*" OR "Patent Ostium Secundum" OR "Persistent Ostium Secundum" OR "Secundum Atrial Defect*") OR ("Ventricular Septal Defect*" OR "Intraventricular Septal Defect*" OR "Intraventricular Septum Defect*" OR "Ventricle Septal Defect*" OR "Ventricular Septum Defect*" OR "Ventricle Septum Defect*" OR "Interventricular Septal Defect*" OR "Interventricular Septum Defect*" OR "Ventricular Heart Septal Defect*" OR "Ventricular Heart Septum Defect" OR "Ventricular Heart Septum Defects" OR "Ventricle Heart Septal Defect" OR "Ventricle Heart Septal Defects" OR "Ventricle Heart Septum Defect" OR "Ventricle Heart Septum Defects" OR "Ventricular Septal Perforation*" OR "Ventricular Septum Perforation*" OR "Ventricle Septal Perforation" OR "Ventricle Septal Perforations" OR "Ventricle Septum Perforation" OR "Ventricle Septum Perforations" OR "Interventricular Shunt*" OR "Intraventricular Shunt*" OR "Membranous Incomplete Septum" OR "Membranous Incomplete Septa") OR ("Aortic Coarctation*" OR "Aorta Coarctation*" OR "Coarctation of Aorta" OR "Coarctation of the Aorta" OR "Aorta Dominant Coarctation" OR "Aorta Dominant Coarctations" OR "Aortic Isthmus Stenosis" OR "Coarctatio Aortae") OR ("Atrioventricular Septal Defect*" OR "Atrioventricular Canal Defect*") OR ("Double Outlet Right Ventricle*" OR "Taussig-Bing Anomal*" OR "Double Outlet Right Heart Ventricle" OR "Right Ventricle Double Outlet" OR "Right Ventricular Double Outlet") OR ("Ebstein*") OR ("Left Heart Hypoplasia Syndrome" OR "Hypoplastic Left Heart Syndrome") OR ("Aortic Arch Interruption*" OR "Aorta Arch Interruption" OR "Aorta Arch Interruptions") OR ("Patent Ductus Arterios*" OR "Patency of the Ductus Arteriosus" OR "Ductus Arteriosus Patency" OR "Ductus Arteriosus Persisten*" OR "Open Ductus Botalli" OR "Patent Ductus Botalli" OR "Persistent Ductus Arteriosus" OR "Persistent Ductus Botalli" OR "Truncus Arteriosus Persistens") OR ("Pulmonic Stenos*" OR "Pulmonary Stenos*" OR "Pulmonary Valve Stenos*" OR "Pulmonary Valva Stenosis" OR "Pulmonary Valva Stenoses" OR "Pulmonal Stenos*" OR "Lung Artery Valve Stenosis" OR "Lung Artery Valve Stenoses" OR "Lung Artery Valvular Stenosis" OR "Lung Artery Valvular Stenoses" OR "Lung Valve Stenosis" OR "Lung Valve Stenoses") OR ("Pulmonary Atresia*" OR "Pulmonary Valve Atresia*" OR "Pulmonic Atresia*" OR "Pulmonic Valve Atresia*" OR "Pulmonary Artery Atresia*" OR "Pulmonic Artery Atresia" OR "Pulmonic Artery Atresias" OR "Lung Atresia*" OR "Lung Artery Atresia" OR "Lung Artery Atresias" OR "Lung Valve Atresia" OR "Lung Valve Atresias" OR "Lung Artery Valve Atresia" OR "Lung Artery Valve Atresias") OR ("Univentricular Heart*" OR "Complex Single Ventricle*" OR "Single Heart Ventricle*" OR "Heart Single Ventricle*" OR "Monoventricular Heart*" OR "Cor Monoventriculare" OR "Cor Triloculare Bia*") OR ("Fallot*") OR ("Scimitar Syndrome" OR "Scimitar Anomaly" OR "Anomalous Pulmonary Venous Return" OR "Pulmonary Venous Return Anomaly" OR "Anomalous Lung Vein Drainage" OR "Anomalous Pulmonary Vein Drainage" OR "Anomalous Pulmonary Venous" OR "Lung Venous Drainage Anomaly" OR "Lung Vein Drainage Anomaly" OR "Lung Venous Return Anomaly" OR "Pulmonary Venous Drainage Anomaly") OR ("Transposition of Great Arter*" OR "Transposition of Great Vessel*" OR "Great Vessels Transposition*" OR "Great Vessel Transposition*" OR "Great Arteries Transposition*" OR "Great Artery Transposition*" OR "Great Vessels Dextrotransposition" OR "Great Vessels Dextrotranspositions" OR "Great Vessels Levotransposition" OR "Great Vessels Levotranspositions" OR "Levotransposition of Great Vessel" OR "Levotransposition of Great Vessesl" OR "Dextrotransposition of Great Vessel*" OR "Dextro-Looped Transposition of the Great Arter*" OR "Dextro Looped Transposition of the Great Arter*" OR "Levo-Looped Transposition of the Great Arter*" OR "Large Vessel Transposition*") OR ("Tricuspid Atresia*" OR "Tricuspid Valve Atresia*" OR "Absent Right Atrioventricular Connection*" OR "Right Atrioventricular Cardiac Valve Atresia" OR "Right Atrioventricular Cardiac Valve Atresias" OR "Right Atrioventricular Cardiac Valvular Atresia" OR "Right Atrioventricular Cardiac Valvular Atresias" OR "Right Atrioventricular Heart Valve Atresia" OR "Right Atrioventricular Heart Valve Atresias" OR "Right Atrioventricular Heart Valvular Atresia" OR "Right Atrioventricular Heart Valvular Atresias" OR "Right Atrioventricular Valve Atresia*" OR "Right Atrioventricular Valvular Atresia" OR "Right Atrioventricular Valvular Atresias" OR "Tricuspid Cardiac Valve Atresia" OR "Tricuspid Cardiac Valve Atresias" OR "Tricuspid Heart Valve Atresia" OR "Tricuspid

Heart Valve Atresias" OR "Tricuspid Valvular Atresia*") OR ("Arterial Trun*" OR "Truncus Arteri*") OR ("Aortic Valve Stenos*" OR "Aortic Stenos*" OR "Aorta Valve Stenosis" OR "Aorta Valve Stenoses" OR "Aortic Valvular Stenos*" OR "Stenosed Aortic Valve*" OR "Stenotic Aortic Valve*"))

Concept 2

TITLE-ABS (("Infect*" OR "Infest*" OR "Inflammation*" OR "TORCH*") OR ("nCoV" OR "2019nCoV" OR "COVID" OR "COVID19" OR "SARS2" OR "cov 2" OR "cov2" OR "coronavirus*" OR "corona virus*" OR "betacoronavirus*" OR "severe acute respiratory syndrome 2" OR "wuhan virus" OR (("wuhan" OR "novel" OR "19" OR "2019" OR "epidem*" OR "pandem*" OR "outbreak" OR "new") AND ("pneumonia virus*" OR "cov" OR "hcov"))) OR ("Rubella*" OR "Three Day Measle*" OR "German Measle*" OR "Epidemic Roseola") OR ("Virus Hepatiti*" OR "Viral Hepatiti*" OR "Virus Liver Disease*" OR "Viral Liver Disease*" OR "Hepatitis Virus Infection*" OR "Hepatitis Viral Infection*" OR "Hepatitis due to Virus*" OR "Hepatitis Caused by Virus*") OR ("Cytomegalovirus*" OR "Salivary Gland Virus*" OR "Beta Herpes Virus 5" OR "Human Herpesvirus 5" OR "Human Herpesvirus V" OR "Human Herpesvirus Type 5" OR "Human Herpesvirus Type V" OR "Herpes Simplex Virus 5" OR "Herpes Simplex Virus V" OR "Herpes Simplex Virus Type 5" OR "Herpes Simplex Virus Type V" OR "Herpes Virus 5" OR "Herpes Virus V" OR "Herpes Virus Type 5" OR "Herpes Virus Type V") OR ("Human Alphaherpesvirus 1" OR "Human Herpesvirus 1" OR "Human Herpesvirus I" OR "Human Herpesvirus Type 1" OR "Human Herpesvirus Type I" OR "Herpes Simplex Virus 1" OR "Herpes Simplex Virus I" OR "Herpes Simplex Virus Type 1" OR "Herpes Simplex Virus Type I" OR "Herpes Virus 1" OR "Herpes Virus I" OR "Herpes Virus Type 1" OR "Herpes Virus Type I") OR ("Human Herpesvirus 2" OR "Human Herpesvirus II" OR "Human Herpesvirus Type 2" OR "Human Herpesvirus Type II" OR "Herpes Simplex Virus 2" OR "Herpes Simplex Virus II" OR "Herpes Simplex Virus Type 2" OR "Herpes Simplex Virus Type II" OR "Herpes Virus 2" OR "Herpes Virus II" OR "Herpes Virus Type 2" OR "Herpes Virus Type II") OR ("Human Herpesvirus 4" OR "Human Herpesvirus IV" OR "Human Herpesvirus Type 4" OR "Human Herpesvirus Type IV" OR "Herpes Simplex Virus 4" OR "Herpes Simplex Virus IV" OR "Herpes Simplex Virus Type 4" OR "Herpes Simplex Virus Type IV" OR "Herpes Virus 4" OR "Herpes Virus IV" OR "Herpes Virus Type 4" OR "Herpes Virus Type IV" OR "Burkitt Herpesvirus" OR "Burkitt Lymphoma Virus" OR "Burkitt's Lymphoma Virus" OR "Burkitts Lymphoma Virus" OR "Infectious Mononucleosis Virus*" OR "Infectious Mononucleosis Herpetovirus" OR "Mononucleosis Infectiosa Virus" OR "Epstein-Barr Virus*" OR "Epstein Barr Virus*" OR "Barr Epstein Virus" OR "Epstein Barr Herpetovirus" OR "Epstein Virus") OR ("Human Herpesvirus 3" OR "Human Herpesvirus III" OR "Human Herpesvirus Type 3" OR "Human Herpesvirus Type III" OR "Herpes Simplex Virus 3" OR "Herpes Simplex Virus III" OR "Herpes Simplex Virus Type 3" OR "Herpes Simplex Virus Type III" OR "Herpes Virus 3" OR "Herpes Virus III" OR "Herpes Virus Type 3" OR "Herpes Virus Type III" OR "Chickenpox" OR "Chicken Pox" OR "Ocular Herpes Zoster Virus" OR "Shingles" OR "Zona Virus" OR "Varicella-Zoster Virus*" OR "Varicella Zoster" OR "Varicella Foster Virus" OR "Varicella Herpes Zoster Virus" OR "Varicella Herpetovirus" OR "Varicella Pneumonia Virus" OR "Varicella Virus" OR "Herpesvirus Varicellae" OR "Herpes Zoster Virus*" OR "Herpes Virus Varicellae" OR "Herpes Zoster Varicella Virus") OR ("Chorio Meningitis" OR "Choriomeningitis ") OR ("Zika Virus*" OR "Zika Flavivirus*") OR ("West Nile Virus*" OR "Egypt 101 Virus*" OR "Kunjin Virus*" OR "West Nile Viral Disease" OR "West Nile Flavivirus Infection" OR "West Nile Infection*" OR "West Nile Viral Infection*") OR ("Rift Valley Fever" OR "Rift Valley Virus*") OR ("B19 Virus*" OR "Parvovirus B19*") OR ("Human Immunodeficiency Virus*" OR "Human T Cell Lymphotropic Virus Type III" OR "Human T-Cell Leukemia Virus Type III" OR "LAV-HTLV-III" OR "Lymphadenopathy-Associated Virus*" OR "Human T Lymphotropic Virus Type III" OR "Acquired Immune Deficiency Syndrome " OR "Acquired Immunodeficiency Syndrome" OR "Aids Associated Lentivirus" OR "Aids Associated Retrovirus" OR "Aids Associated Virus" OR "Aids Related Virus" OR "Human Immuno Deficiency Virus" OR "Immunodeficiency Associated Virus" OR "Lymphadenopathy Associated Retrovirus") OR ("Coxsackievir*" OR "Coxsackie Vir*") OR ("Listeria Monocytogenes" OR "Listerella Hepatolytica" OR "Listeriosis Monocytogenes" OR "Bacterium Monocytogenes" OR "Corynebacterium Infantisepticum" OR "Corynebacterium Parvulum" OR "Erysipelothrix Monocytogenes") OR ("Treponema Pallid*" OR "Treponema Reiterii" OR "Spirochaeta Pallida") OR ("Streptococcus B" OR "Streptococcus Group B" OR "Group B Streptococcus" OR "Staphylococcus Agalactiae" OR "Streptococcus Mastitidis" OR "Streptococcus Nocardi" OR "Streptococcus Agalactiae") OR ("Staphylococcus Aureus" OR "Staphylococcus Pyogenes aureus" OR "Staphylococcus Pyogenes Citreus" OR "Micrococcus Aureus" OR "Micrococcus Pyogenes") OR ("Enterococcus Coli" OR "Escherichia Coli" OR "Bacillus Coli" OR "Bacterium Coli" OR "Alkalescens-Dispar Group" OR "EAggEC" OR "Bacillus Escherichii" OR "Bacterium E3" OR "Coli Bacillus" OR "Coli Bacterium" OR "Colibacillus" OR "Colon Bacillus" OR "Escherichia Alkalescens Dispart") OR ("Chlamydia Trachomatis" OR "Chlamidia Trachomatis" OR "Chlamydophila Trachomatis" OR "Chlamydozoon Trachomatis"

OR "Rickettsia Trachomae" OR "Rickettsia Trachomatis") OR ("Helicobacter Pylori" OR "Helicobacter Nemestrinae" OR "Campylobacter Pylori") OR ("Plasmodium Falciparum*") OR ("Plasmodium Vivax*") OR ("Toxoplasma*" OR "Toxoplasma Gondi*" OR "Toxoplasma Hominis") OR ("Trypanosoma cruz*" OR "Schizotrypanum Cruzi")) OR AUTHKEY (("Infect*" OR "Infest*" OR "Inflammation*" OR "TORCH*") OR ("nCoV" OR "2019nCoV" OR "COVID" OR "COVID19" OR "SARS2" OR "cov 2" OR "cov2" OR "coronavirus*" OR "corona virus*" OR "betacoronavirus*" OR "severe acute respiratory syndrome 2" OR "wuhan virus" OR (("wuhan" OR "novel" OR "19" OR "2019" OR "epidem*" OR "pandem*" OR "outbreak" OR "new") AND ("pneumonia virus*" OR "cov" OR "hcov"))) OR ("Rubella*" OR "Three Day Measle*" OR "German Measle*" OR "Epidemic Roseola") OR ("Virus Hepatiti*" OR "Viral Hepatiti*" OR "Virus Liver Disease*" OR "Viral Liver Disease*" OR "Hepatitis Virus Infection*" OR "Hepatitis Viral Infection*" OR "Hepatitis due to Virus*" OR "Hepatitis Caused by Virus*") OR ("Cytomegalovirus*" OR "Salivary Gland Virus*" OR "Beta Herpes Virus 5" OR "Human Herpesvirus 5" OR "Human Herpesvirus V" OR "Human Herpesvirus Type 5" OR "Human Herpesvirus Type V" OR "Herpes Simplex Virus 5" OR "Herpes Simplex Virus V" OR "Herpes Simplex Virus Type 5" OR "Herpes Simplex Virus Type V" OR "Herpes Virus 5" OR "Herpes Virus V" OR "Herpes Virus Type 5" OR "Herpes Virus Type V") OR ("Human Alphaherpesvirus 1" OR "Human Herpesvirus 1" OR "Human Herpesvirus I" OR "Human Herpesvirus Type 1" OR "Human Herpesvirus Type I" OR "Herpes Simplex Virus 1" OR "Herpes Simplex Virus I" OR "Herpes Simplex Virus Type 1" OR "Herpes Simplex Virus Type I" OR "Herpes Virus 1" OR "Herpes Virus I" OR "Herpes Virus Type 1" OR "Herpes Virus Type I") OR ("Human Herpesvirus 2" OR "Human Herpesvirus II" OR "Human Herpesvirus Type 2" OR "Human Herpesvirus Type II" OR "Herpes Simplex Virus 2" OR "Herpes Simplex Virus II" OR "Herpes Simplex Virus Type 2" OR "Herpes Simplex Virus Type II" OR "Herpes Virus 2" OR "Herpes Virus II" OR "Herpes Virus Type 2" OR "Herpes Virus Type II") OR ("Human Herpesvirus 4" OR "Human Herpesvirus IV" OR "Human Herpesvirus Type 4" OR "Human Herpesvirus Type IV" OR "Herpes Simplex Virus 4" OR "Herpes Simplex Virus IV" OR "Herpes Simplex Virus Type 4" OR "Herpes Simplex Virus Type IV" OR "Herpes Virus 4" OR "Herpes Virus IV" OR "Herpes Virus Type 4" OR "Herpes Virus Type IV" OR "Burkitt Herpesvirus" OR "Burkitt Lymphoma Virus" OR "Burkitt's Lymphoma Virus" OR "Burkitts Lymphoma Virus" OR "Infectious Mononucleosis Virus*" OR "Infectious Mononucleosis Herpetovirus" OR "Mononucleosis Infectiosa Virus" OR "Epstein-Barr Virus*" OR "Epstein Barr Virus*" OR "Barr Epstein Virus" OR "Epstein Barr Herpetovirus" OR "Epstein Virus") OR ("Human Herpesvirus 3" OR "Human Herpesvirus III" OR "Human Herpesvirus Type 3" OR "Human Herpesvirus Type III" OR "Herpes Simplex Virus 3" OR "Herpes Simplex Virus III" OR "Herpes Simplex Virus Type 3" OR "Herpes Simplex Virus Type III" OR "Herpes Virus 3" OR "Herpes Virus III" OR "Herpes Virus Type 3" OR "Herpes Virus Type III" OR "Chickenpox" OR "Chicken Pox" OR "Ocular Herpes Zoster Virus" OR "Shingles" OR "Zona Virus" OR "Varicella-Zoster Virus*" OR "Varicella Zoster" OR "Varicella Foster Virus" OR "Varicella Herpes Zoster Virus" OR "Varicella Herpetovirus" OR "Varicella Pneumonia Virus" OR "Varicella Virus" OR "Herpesvirus Varicellae" OR "Herpes Zoster Virus*" OR "Herpes Virus Varicellae" OR "Herpes Zoster Varicella Virus") OR ("Chorio Meningitis" OR "Choriomeningitis ") OR ("Zika Virus*" OR "Zika Flavivirus*") OR ("West Nile Virus*" OR "Egypt 101 Virus*" OR "Kunjin Virus*" OR "West Nile Viral Disease" OR "West Nile Flavivirus Infection" OR "West Nile Infection*" OR "West Nile Viral Infection*") OR ("Rift Valley Fever" OR "Rift Valley Virus*") OR ("B19 Virus*" OR "Parvovirus B19*") OR ("Human Immunodeficiency Virus*" OR "Human T Cell Lymphotropic Virus Type III" OR "Human T-Cell Leukemia Virus Type III" OR "LAV-HTLV-III" OR "Lymphadenopathy-Associated Virus*" OR "Human T Lymphotropic Virus Type III" OR "Acquired Immune Deficiency Syndrome " OR "Acquired Immunodeficiency Syndrome" OR "Aids Associated Lentivirus" OR "Aids Associated Retrovirus" OR "Aids Associated Virus" OR "Aids Related Virus" OR "Human Immuno Deficiency Virus" OR "Immunodeficiency Associated Virus" OR "Lymphadenopathy Associated Retrovirus") OR ("Coxsackievir*" OR "Coxsackie Vir*") OR ("Listeria Monocytogenes" OR "Listerella Hepatolytica" OR "Listeriosis Monocytogenes" OR "Bacterium Monocytogenes" OR "Corynebacterium Infantisepticum" OR "Corynebacterium Parvulum" OR "Erysipelothrix Monocytogenes") OR ("Treponema Pallid*" OR "Treponema Reiterii" OR "Spirochaeta Pallida") OR ("Streptococcus B" OR "Streptococcus Group B" OR "Group B Streptococcus" OR "Staphylococcus Agalactiae" OR "Streptococcus Mastitidis" OR "Streptococcus Nocardi" OR "Streptococcus Agalactiae") OR ("Staphylococcus Aureus" OR "Staphylococcus Pyogenes aureus" OR "Staphylococcus Pyogenes Citreus" OR "Micrococcus Aureus" OR "Micrococcus Pyogenes") OR ("Enterococcus Coli" OR "Escherichia Coli" OR "Bacillus Coli" OR "Bacterium Coli" OR "Alkalescens-Dispar Group" OR "EAggEC" OR "Bacillus Escherichii" OR "Bacterium E3" OR "Coli Bacillus" OR "Coli Bacterium" OR "Colibacillus" OR "Colon Bacillus" OR "Escherichia Alkalescens Dispart") OR ("Chlamydia Trachomatis" OR "Chlamidia Trachomatis" OR "Chlamydophila Trachomatis" OR "Chlamydozoon Trachomatis" OR "Rickettsia Trachomae" OR "Rickettsia Trachomatis") OR ("Helicobacter Pylori" OR "Helicobacter Nemestrinae" OR "Campylobacter Pylori") OR ("Plasmodium Falciparum*") OR ("Plasmodium Vivax*") OR ("Toxoplasma*" OR "Toxoplasma Gondi*" OR "Toxoplasma Hominis") OR ("Trypanosoma cruz*" OR

“Schizotrypanum Cruzi”))

Concept 3:

TITLE-ABS (“Maternal” OR "Transplacental” OR “Prenatal” OR “Mother*” OR “Pregnan*” OR “Women” OR “Woman” OR “Female” OR “In Utero” OR “Uteroplacental”) OR AUTHKEY (“Maternal” OR "Transplacental” OR “Prenatal” OR “Mother*” OR “Pregnan*” OR “Women” OR “Woman” OR “Female” OR “In Utero” OR “Uteroplacental”)

Cochrane Library: Cochrane Library: CENTRAL & CDSR

(Concept 1) AND (Concept 2) AND (Concept 3)

Concept 1:

#1:

([mh ^“Heart Defects, Congenital”] OR [mh “Heart Septal Defects, Atrial”] OR [mh “Heart Septal Defects, Ventricular”] OR [mh “Aortic Coarctation”] OR [mh “Double Outlet Right Ventricle“] OR [mh “Ebstein Anomaly”] OR [mh “Hypoplastic Left Heart Syndrome”] OR [mh “Ductus Arteriosus, Patent“] OR [mh “Pulmonary Valve Stenosis”] OR [mh “Pulmonary Atresia”] OR [mh “Univentricular Heart”] OR [mh “Tetralogy of Fallot”] OR [mh “Scimitar Syndrome“] OR [mh “Transposition of Great Vessels”] OR [mh “Tricuspid Atresia”] OR [mh “Truncus Arteriosus”] OR [mh “Aortic Valve Stenosis”])

#2:

(((Congenital NEXT Heart NEXT Defect*) OR (Congenital NEXT Heart NEXT Disease*) OR (Congenital NEXT Cardiac NEXT Disease*) OR (Congenital NEXT Cardiac NEXT Distress) OR (Congenital NEXT Cardiac NEXT Distresses) OR (Congenital NEXT Heart NEXT Distress) OR (Congenital NEXT Heart NEXT Distresses) OR (Congenital NEXT Heart NEXT Failure) OR (Heart NEXT Congenital NEXT Disease*) OR (Heart NEXT Congenital NEXT Defect*) OR (Heart NEXT Congenital NEXT Anomaly) OR (Heart NEXT Congenital NEXT Anomalies) OR (Heart NEXT Congenital NEXT Malformation*) OR (Neonatal NEXT Cardiopathy) OR (Congenital NEXT Heart NEXT Malformation*) OR (Congenital NEXT Malformation NEXT of NEXT the NEXT Heart) OR (Congenital NEXT Malformations NEXT of NEXT the NEXT Heart) OR (Congenital NEXT Heart NEXT Anomal*) OR (Congenital NEXT Cardiac NEXT Defect*) OR (Congenital NEXT Cardiac NEXT Malformation*) OR (Congenital NEXT Cardiac NEXT Anomal*) OR (Congenital NEXT Cardiovascular NEXT Disease*)) OR ((Atrial NEXT Septal NEXT Defect*) OR (Atrium NEXT septum NEXT defect*) OR (Persistent NEXT Ostium NEXT Primum) OR (Atrial NEXT Heart NEXT Septal NEXT Defect*) OR (Atrial NEXT Heart NEXT Shunt) OR (Atrial NEXT Septum NEXT Defect*) OR (Atrium NEXT Heart NEXT Septal NEXT Defect) OR (Atrium NEXT Heart NEXT Septal NEXT Defects) OR (Atrial NEXT Heart NEXT Septum NEXT Defect) OR (Atrial NEXT Heart NEXT Septum NEXT Defects) OR (Atrium NEXT Heart NEXT Septum NEXT Defect) OR (Atrium NEXT Heart NEXT Septum NEXT Defects) OR (Atrium NEXT Septal NEXT Defect*) OR (Atrium NEXT Septum NEXT Primum NEXT Defect) OR (Atrium NEXT Septum NEXT Primum NEXT Defects) OR (Atrial NEXT Septum NEXT Primum NEXT Defect) OR (Atrial NEXT Septum NEXT Primum NEXT Defects) OR (Atrium NEXT Septum NEXT Secundum NEXT Defect) OR (Atrium NEXT Septum NEXT Secundum NEXT Defects) OR (Atrial NEXT Septum NEXT Secundum NEXT Defect) OR (Atrial NEXT Septum NEXT Secundum NEXT Defects) OR (Cleft NEXT Heart NEXT Atrium) OR (Interatrial NEXT Septal NEXT Defect*) OR (Interatrial NEXT Septum NEXT Defect*) OR (Interauricular NEXT Septal NEXT Defect*) OR (Lutembacher*) OR (Patent NEXT Ostium NEXT Secundum) OR (Persistent NEXT Ostium NEXT Secundum) OR (Secundum NEXT Atrial NEXT Defect*)) OR ((Ventricular NEXT Septal NEXT Defect*) OR (Intraventricular NEXT Septal NEXT Defect*) OR (Intraventricular NEXT Septum NEXT Defect*) OR (Ventricle NEXT Septal NEXT Defect*) OR (Ventricular NEXT Septum NEXT Defect*) OR (Ventricle NEXT Septum NEXT Defect*) OR (Interventricular NEXT Septal NEXT Defect*) OR (Interventricular NEXT Septum NEXT Defect*) OR (Ventricular NEXT Heart NEXT Septal NEXT Defect*) OR (Ventricular NEXT Heart NEXT Septum NEXT Defect) OR (Ventricular NEXT Heart NEXT Septum NEXT Defects) OR (Ventricle NEXT Heart NEXT Septal NEXT Defect) OR (Ventricle NEXT Heart NEXT Septal NEXT Defects) OR (Ventricle NEXT Heart NEXT Septum NEXT Defect) OR (Ventricle NEXT Heart NEXT Septum NEXT Defects) OR (Ventricular NEXT Septal NEXT Perforation*) OR (Ventricular NEXT Septum NEXT Perforation*) OR (Ventricle NEXT Septal NEXT Perforation) OR (Ventricle NEXT

Septal NEXT Perforations) OR (Ventricle NEXT Septum NEXT Perforation) OR (Ventricle NEXT Septum NEXT Perforations) OR (Interventricular NEXT Shunt*) OR (Intraventricular NEXT Shunt*) OR (Membranous NEXT Incomplete NEXT Septum) OR (Membranous NEXT Incomplete NEXT Septa)) OR ((Aortic NEXT Coarctation*) OR (Aorta NEXT Coarctation*) OR (Coarctation NEXT of NEXT Aorta) OR (Coarctation NEXT of NEXT the NEXT Aorta) OR (Aorta NEXT Dominant NEXT Coarctation) OR (Aorta NEXT Dominant NEXT Coarctations) OR (Aortic NEXT Isthmus NEXT Stenosis) OR (Coarctatio NEXT Aortae)) OR ((Atrioventricular NEXT Septal NEXT Defect*) OR (Atrioventricular NEXT Canal NEXT Defect*)) OR ((Double NEXT Outlet NEXT Right NEXT Ventricle*) OR (Taussig-Bing NEXT Anomal*) OR (Double NEXT Outlet NEXT Right NEXT Heart NEXT Ventricle) OR (Right Ventricle Double Outlet) OR (Right Ventricular Double Outlet)) OR ((Ebstein*)) OR ((Left NEXT Heart NEXT Hypoplasia NEXT Syndrome) OR (Hypoplastic NEXT Left NEXT Heart NEXT Syndrome)) OR ((Aortic NEXT Arch NEXT Interruption*) OR (Aorta NEXT Arch NEXT Interruption) OR (Aorta NEXT Arch NEXT Interruptions)) OR ((Patent NEXT Ductus NEXT Arterios*) OR (Patency NEXT of NEXT the NEXT Ductus NEXT Arteriosus) OR (Ductus NEXT Arteriosus NEXT Patency) OR (Ductus NEXT Arteriosus NEXT Persisten*) OR (Open NEXT Ductus NEXT Botalli) OR (Patent NEXT Ductus NEXT Botalli) OR (Persistent NEXT Ductus NEXT Arteriosus) OR (Persistent NEXT Ductus NEXT Botalli) OR (Truncus NEXT Arteriosus NEXT Persistens)) OR ((Pulmonic NEXT Stenos*) OR (Pulmonary NEXT Stenos*) OR (Pulmonary NEXT Valve NEXT Stenos*) OR (Pulmonary NEXT Valva NEXT Stenosis) OR (Pulmonary NEXT Valva NEXT Stenoses) OR (Pulmonal NEXT Stenos*) OR (Lung NEXT Artery NEXT Valve NEXT Stenosis) OR (Lung NEXT Artery NEXT Valve NEXT Stenoses) OR (Lung NEXT Artery NEXT Valvular NEXT Stenosis) OR (Lung NEXT Artery NEXT Valvular NEXT Stenoses) OR (Lung NEXT Valve NEXT Stenosis) OR (Lung NEXT Valve NEXT Stenoses)) OR ((Pulmonary NEXT Atresia*) OR (Pulmonary NEXT Valve NEXT Atresia*) OR (Pulmonic NEXT Atresia*) OR (Pulmonic NEXT Valve NEXT Atresia*) OR (Pulmonary NEXT Artery NEXT Atresia*) OR (Pulmonic NEXT Artery NEXT Atresia) OR (Pulmonic NEXT Artery NEXT Atresias) OR (Lung NEXT Atresia*) OR (Lung NEXT Artery NEXT Atresia) OR (Lung NEXT Artery NEXT Atresias) OR (Lung NEXT Valve NEXT Atresia) OR (Lung NEXT Valve NEXT Atresias) OR (Lung NEXT Artery NEXT Valve NEXT Atresia) OR (Lung NEXT Artery NEXT Valve NEXT Atresias)) OR ((Univentricular NEXT Heart*) OR (Complex NEXT Single NEXT Ventricle*) OR (Single NEXT Heart NEXT Ventricle*) OR (Heart NEXT Single NEXT Ventricle*) OR (Monoventricular NEXT Heart*) OR (Cor NEXT Monoventriculare) OR (Cor NEXT Triloculare NEXT Bia*)) OR ((Fallot*)) OR ((Scimitar NEXT Syndrome) OR (Scimitar NEXT Anomaly) OR (Anomalous NEXT Pulmonary NEXT Venous NEXT Return) OR (Pulmonary NEXT Venous NEXT Return NEXT Anomaly) OR (Anomalous NEXT Lung NEXT Vein NEXT Drainage) OR (Anomalous NEXT Pulmonary NEXT Vein NEXT Drainage) OR (Anomalous NEXT Pulmonary NEXT Venous) OR (Lung NEXT Venous NEXT Drainage NEXT Anomaly) OR (Lung NEXT Vein NEXT Drainage NEXT Anomaly) OR (Lung NEXT Venous NEXT Return NEXT Anomaly) OR (Pulmonary NEXT Venous NEXT Drainage NEXT Anomaly)) OR ((Transposition NEXT of NEXT Great NEXT Arter*) OR (Transposition NEXT of NEXT Great NEXT Vessel*) OR (Great NEXT Vessels NEXT Transposition*) OR (Great NEXT Vessel NEXT Transposition*) OR (Great NEXT Arteries NEXT Transposition*) OR (Great NEXT Artery NEXT Transposition*) OR (Great NEXT Vessels NEXT Dextrotransposition) OR (Great NEXT Vessels NEXT Dextrotranspositions) OR (Great NEXT Vessels NEXT Levotransposition) OR (Great NEXT Vessels NEXT Levotranspositions) OR (Levotransposition NEXT of NEXT Great NEXT Vessel) OR (Levotransposition NEXT of NEXT Great NEXT Vessesl) OR (Dextrotransposition NEXT of NEXT Great NEXT Vessel*) OR (Dextro-Looped NEXT Transposition NEXT of NEXT the NEXT Great NEXT Arter*) OR (Dextro NEXT Looped NEXT Transposition NEXT of NEXT the NEXT Great NEXT Arter*) OR (Levo-Looped NEXT Transposition NEXT of NEXT the NEXT Great NEXT Arter*) OR (Large NEXT Vessel NEXT Transposition*)) OR ((Tricuspid NEXT Atresia*) OR (Tricuspid NEXT Valve NEXT Atresia*) OR (Absent NEXT Right NEXT Atrioventricular NEXT Connection*) OR (Right NEXT Atrioventricular NEXT Cardiac NEXT Valve NEXT Atresia) OR (Right NEXT Atrioventricular NEXT Cardiac NEXT Valve NEXT Atresias) OR (Right NEXT Atrioventricular NEXT Cardiac NEXT Valvular NEXT Atresia) OR (Right NEXT Atrioventricular NEXT Cardiac NEXT Valvular NEXT Atresias) OR (Right NEXT Atrioventricular NEXT Heart NEXT Valve NEXT Atresia) OR (Right NEXT Atrioventricular NEXT Heart NEXT Valve NEXT Atresias) OR (Right NEXT Atrioventricular NEXT Heart NEXT Valvular NEXT Atresia) OR (Right NEXT Atrioventricular NEXT Heart NEXT Valvular NEXT Atresias) OR (Right NEXT Atrioventricular NEXT Valve NEXT Atresia*) OR (Right NEXT Atrioventricular NEXT Valvular NEXT Atresia) OR (Right NEXT Atrioventricular NEXT Valvular NEXT Atresias) OR (Tricuspid NEXT Cardiac NEXT Valve NEXT Atresia) OR (Tricuspid NEXT Cardiac NEXT Valve NEXT Atresias) OR (Tricuspid NEXT Heart NEXT Valve NEXT Atresia) OR (Tricuspid NEXT Heart NEXT Valve NEXT Atresias) OR (Tricuspid NEXT Valvular NEXT Atresia*)) OR ((Arterial NEXT Trun*) OR (Truncus NEXT Arteri*)) OR ((Aortic NEXT Valve NEXT Stenos*) OR (Aortic NEXT Stenos*) OR (Aorta NEXT Valve NEXT Stenosis) OR (Aorta NEXT Valve NEXT Stenoses) OR (Aortic NEXT Valvular NEXT Stenos*) OR (Stenosed NEXT Aortic NEXT Valve*) OR (Stenotic NEXT Aortic NEXT Valve*))):ti,ab,kw

#3：

#1 OR #2

Concept 2:

#4:

([mh ^"Infections"] OR [mh "SARS-CoV-2"] OR [mh "COVID-19"] OR [mh "Rubella"] OR [mh "Rubella virus"] OR [mh "Hepatitis, Viral, Human"] OR [mh "Cytomegalovirus"] OR [mh "Herpesvirus 1, Human"] OR [mh "Herpesvirus 2, Human"] OR [mh "Herpesvirus 4, Human"] OR [mh "Herpesvirus 3, Human"] OR [mh "Lymphocytic choriomeningitis virus"] OR [mh "Zika Virus"] OR [mh "West Nile virus"] OR [mh "Rift Valley fever virus"] OR [mh "Parvovirus B19, Human"] OR [mh "HIV"] OR [mh "Coxsackievirus Infection"] OR [mh "Listeria monocytogenes"] OR [mh "Treponema pallidum"] OR [mh "Streptococcus agalactiae"] OR [mh "Staphylococcus aureus"] OR [mh "Escherichia coli"] OR [mh "Chlamydia trachomatis"] OR [mh "Helicobacter pylori"] OR [mh "Plasmodium falciparum"] OR [mh "Plasmodium vivax"] OR [mh "Toxoplasma"] OR [mh "Trypanosoma cruzi"])

#5:

(((Infect*) OR (Infest*) OR (Inflammation*) OR (TORCH*)) OR ((nCoV OR 2019nCoV OR COVID OR COVID19 OR SARS2 OR "cov 2" OR cov2 OR coronavirus* OR (corona NEXT virus*) OR betacoronavirus* OR "severe acute respiratory syndrome 2" OR "Wuhan virus" OR ((wuhan OR novel OR new OR 19 OR 2019 OR epidem* OR pandem* OR outbreak) AND ([mh ^"coronavirus"] OR [mh ^" Betacoronavirus"] OR [mh ^"Coronavirus Infections"] OR (pneumonia NEXT virus*) OR cov OR hcov)))) OR ((Rubella*) OR (Three NEXT Day NEXT Measle*) OR (German NEXT Measle*) OR (Epidemic NEXT Roseola)) OR ((Virus NEXT Hepatiti*) OR (Viral NEXT Hepatiti*) OR (Virus NEXT Liver NEXT Disease*) OR (Viral NEXT Liver NEXT Disease*) OR (Hepatitis NEXT Virus NEXT Infection*) OR (Hepatitis NEXT Viral NEXT Infection*) OR (Hepatitis NEXT due NEXT to NEXT Virus*) OR (Hepatitis NEXT Caused NEXT by NEXT Virus*)) OR ((Cytomegalovirus*) OR (Salivary NEXT Gland NEXT Virus*) OR (Beta NEXT Herpes NEXT Virus 5) OR (Human NEXT Herpesvirus NEXT 5) OR (Human NEXT Herpesvirus NEXT V) OR (Human NEXT Herpesvirus NEXT Type 5) OR (Human NEXT Herpesvirus NEXT Type NEXT V) OR (Herpes NEXT Simplex NEXT Virus NEXT 5) OR (Herpes NEXT Simplex NEXT Virus NEXT V) OR (Herpes NEXT Simplex NEXT Virus NEXT Type 5) OR (Herpes NEXT Simplex NEXT Virus NEXT Type NEXT V) OR (Herpes NEXT Virus NEXT 5) OR (Herpes NEXT Virus NEXT V) OR (Herpes NEXT Virus NEXT Type NEXT 5) OR (Herpes NEXT Virus NEXT Type NEXT V)) OR ((Human NEXT Alphaherpesvirus NEXT 1) OR (Human NEXT Herpesvirus NEXT 1) OR (Human NEXT Herpesvirus NEXT I) OR (Human NEXT Herpesvirus NEXT Type NEXT 1) OR (Human NEXT Herpesvirus NEXT Type NEXT I) OR (Herpes NEXT Simplex NEXT Virus NEXT 1) OR (Herpes NEXT Simplex NEXT Virus I) OR (Herpes NEXT Simplex NEXT Virus NEXT Type 1) OR (Herpes NEXT Simplex NEXT Virus NEXT Type NEXT I) OR (Herpes NEXT Virus NEXT 1) OR (Herpes NEXT Virus NEXT I) OR (Herpes NEXT Virus NEXT Type NEXT 1) OR (Herpes NEXT Virus NEXT Type NEXT I)) OR ((Human NEXT Herpesvirus NEXT 2) OR (Human NEXT Herpesvirus NEXT II) OR (Human NEXT Herpesvirus NEXT Type NEXT 2) OR (Human NEXT Herpesvirus NEXT Type NEXT II) OR (Herpes NEXT Simplex NEXT Virus NEXT 2) OR (Herpes NEXT Simplex NEXT Virus NEXT II) OR (Herpes NEXT Simplex NEXT Virus NEXT Type NEXT 2) OR (Herpes NEXT Simplex NEXT Virus NEXT Type NEXT II) OR (Herpes NEXT Virus NEXT 2) OR (Herpes NEXT Virus NEXT II) OR (Herpes NEXT Virus NEXT Type NEXT 2) OR (Herpes NEXT Virus NEXT Type NEXT II)) OR ((Human NEXT Herpesvirus NEXT 4) OR (Human NEXT Herpesvirus NEXT IV) OR (Human NEXT Herpesvirus NEXT Type NEXT 4) OR (Human NEXT Herpesvirus NEXT Type NEXT IV) OR (Herpes NEXT Simplex NEXT Virus NEXT 4) OR (Herpes NEXT Simplex NEXT Virus NEXT IV) OR (Herpes NEXT Simplex NEXT Virus NEXT Type NEXT 4) OR (Herpes NEXT Simplex NEXT Virus NEXT Type NEXT IV) OR (Herpes NEXT Virus NEXT 4) OR (Herpes NEXT Virus NEXT IV) OR (Herpes NEXT Virus NEXT Type NEXT 4) OR (Herpes NEXT Virus NEXT Type NEXT IV) OR (Burkitt NEXT Herpesvirus) OR (Burkitt NEXT Lymphoma NEXT Virus) OR (Burkitt's NEXT Lymphoma NEXT Virus) OR (Burkitts NEXT Lymphoma NEXT Virus) OR (Infectious NEXT Mononucleosis NEXT Virus*) OR (Infectious NEXT Mononucleosis NEXT Herpetovirus) OR (Mononucleosis NEXT Infectiosa NEXT Virus) OR (Epstein-Barr NEXT Virus*) OR (Epstein NEXT Barr NEXT Virus*) OR (Barr NEXT Epstein NEXT Virus) OR (Epstein NEXT Barr NEXT Herpetovirus) OR (Epstein NEXT Virus)) OR ((Human NEXT Herpesvirus NEXT 3) OR (Human NEXT Herpesvirus NEXT III) OR (Human NEXT Herpesvirus NEXT Type NEXT 3) OR (Human NEXT Herpesvirus NEXT Type NEXT III) OR (Herpes NEXT Simplex NEXT Virus NEXT 3) OR (Herpes NEXT Simplex NEXT Virus NEXT III) OR (Herpes NEXT Simplex NEXT Virus NEXT Type NEXT 3) OR (Herpes NEXT Simplex NEXT Virus NEXT Type NEXT III) OR (Herpes NEXT Virus NEXT 3) OR (Herpes NEXT Virus NEXT III) OR (Herpes NEXT Virus NEXT Type NEXT 3) OR (Herpes NEXT Virus NEXT Type NEXT III) OR (Chickenpox) OR (Chicken NEXT Pox) OR (Ocular NEXT Herpes NEXT Zoster NEXT

Virus) OR (Shingles) OR (Zona NEXT Virus) OR (Varicella-Zoster NEXT Virus*) OR (Varicella NEXT Zoster) OR (Varicella NEXT Foster NEXT Virus) OR (Varicella NEXT Herpes NEXT Zoster NEXT Virus) OR (Varicella NEXT Herpetovirus) OR (Varicella NEXT Pneumonia NEXT Virus) OR (Varicella NEXT Virus) OR (Herpesvirus NEXT Varicellae) OR (Herpes NEXT Zoster NEXT Virus*) OR (Herpes NEXT Virus NEXT Varicellae) OR (Herpes NEXT Zoster NEXT Varicella NEXT Virus)) OR ((Chorio NEXT Meningitis) OR (Choriomeningitis)) OR ((Zika NEXT Virus*) OR (Zika NEXT Flavivirus*)) OR ((West NEXT Nile NEXT Virus*) OR (Egypt NEXT 101 NEXT Virus*) OR (Kunjin NEXT Virus*) OR (West NEXT Nile NEXT Viral NEXT Disease) OR (West NEXT Nile NEXT Flavivirus NEXT Infection) OR (West NEXT Nile NEXT Infection*) OR (West NEXT Nile NEXT Viral NEXT Infection*)) OR ((Rift NEXT Valley NEXT Fever) OR (Rift NEXT Valley NEXT Virus*)) OR ((B19 NEXT Virus*) OR (Parvovirus NEXT B19*)) OR ((Human NEXT Immunodeficiency NEXT Virus*) OR (Human NEXT T NEXT Cell NEXT Lymphotropic NEXT Virus NEXT Type NEXT III) OR (Human NEXT T-Cell NEXT Leukemia NEXT Virus NEXT Type NEXT III) OR (LAV-HTLV-III) OR (Lymphadenopathy-Associated NEXT Virus*) OR (Human NEXT T NEXT Lymphotropic NEXT Virus NEXT Type NEXT III) OR (Acquired NEXT Immune NEXT Deficiency NEXT Syndrome) OR (Acquired NEXT Immunodeficiency NEXT Syndrome) OR (Aids NEXT Associated NEXT Lentivirus) OR (Aids NEXT Associated NEXT Retrovirus) OR (Aids NEXT Associated NEXT Virus) OR (Aids NEXT Related NEXT Virus) OR (Human NEXT Immuno NEXT Deficiency NEXT Virus) OR (Immunodeficiency NEXT Associated NEXT Virus) OR (Lymphadenopathy NEXT Associated NEXT Retrovirus)) OR ((Coxsackievir*) OR (Coxsackie NEXT Vir*)) OR ((Listeria NEXT Monocytogenes) OR (Listerella NEXT Hepatolytica) OR (Listeriosis NEXT Monocytogenes) OR (Bacterium NEXT Monocytogenes) OR (Corynebacterium NEXT Infantisepticum) OR (Corynebacterium NEXT Parvulum) OR (Erysipelothrix NEXT Monocytogenes)) OR ((Treponema NEXT Pallid*) OR (Treponema NEXT Reiterii) OR (Spirochaeta NEXT Pallida)) OR ((Streptococcus NEXT B) OR (Streptococcus NEXT Group NEXT B) OR (Group NEXT B NEXT Streptococcus) OR (Staphylococcus NEXT Agalactiae) OR (Streptococcus NEXT Mastitidis) OR (Streptococcus NEXT Nocardi) OR (Streptococcus NEXT Agalactiae)) OR ((Staphylococcus NEXT Aureus) OR (Staphylococcus NEXT Pyogenes NEXT Aureus) OR (Staphylococcus NEXT Pyogenes NEXT Citreus) OR (Micrococcus NEXT Aureus) OR (Micrococcus NEXT Pyogenes)) OR ((Enterococcus NEXT Coli) OR (Escherichia NEXT Coli) OR (Bacillus NEXT Coli) OR (Bacterium NEXT Coli) OR (Alkalescens-Dispar NEXT Group) OR (EAggEC) OR (Bacillus NEXT Escherichii) OR (Bacterium NEXT E3) OR (Coli NEXT Bacillus) OR (Coli NEXT Bacterium) OR (Colibacillus) OR (Colon NEXT Bacillus) OR (Escherichia NEXT Alkalescens NEXT Dispart)) OR ((Chlamydia NEXT Trachomatis) OR (Chlamidia NEXT Trachomatis) OR (Chlamydophila NEXT Trachomatis) OR (Chlamydozoon NEXT Trachomatis) OR (Rickettsia NEXT Trachomae) OR (Rickettsia NEXT Trachomatis)) OR ((Helicobacter NEXT Pylori) OR (Helicobacter NEXT Nemestrinae) OR (Campylobacter NEXT Pylori)) OR ((Plasmodium NEXT Falciparum*)) OR ((Plasmodium NEXT Vivax*)) OR ((Toxoplasma*) OR (Toxoplasma NEXT Gondi*) OR (Toxoplasma NEXT Hominis)) OR ((Trypanosoma NEXT cruz*) OR (Schizotrypanum NEXT Cruzi))):ti,ab,kw

#6:

#4 OR #5

Concept 3:

#7:

([mh "Maternal Exposure"] OR [mh “Prenatal Exposure Delayed Effects”] OR [mh “Maternal-Fetal Exchange”])

#8:

((Maternal) OR (Transplacental) OR (Prenatal) OR (Mother*) OR (Pregnan*) OR (Women) OR (Woman) OR (Female) OR (In NEXT Utero) OR (Uteroplacental)):ti,ab,kw

#9:

#7 OR #8

COMBINEREN:

#10:

#3 AND #6 AND #9

Table S3. Characteristic of 9 studies of maternal infections at other/unspecific timepoints during pregnancy and risk of congenital heart defects in offspring

Study	Study region	Study design	Study period	Study setting	Sample size	Investigated exposures	Exposure timing	Reported outcomes	Matching/adjusting variables
Strzelecka et al. (2023) [34]	Poland (Europe)	Cohort study	Oct. 2021-May 2022	Hospital based	55 CHD cases and 182 non-CHD cases	SARS-CoV-2 infection	NA	CHD	NA
Mamun et al. (2023) [35]	Bangladesh (Asia)	Case-control	July 2018-July 2019	Hospital based	260 CHD cases and 280 controls	Urinary tract infection (2.78%);† rubella (1.11%); cytomegalovirus (0.37%); dengue (1.67%); chicken pox (1.67%)	During pregnancy	CHD	NA
Appiah et al. (2023) [12]	USA (North America)	Cohort study	2011-2020	Population-based	22,928 CCHD cases and 35,359,360 non-CCHD cases	Infection (2.62%);† sexually transmitted infection; gonorrhea (0.28%); syphilis (0.10%); chlamydia; hepatitis B (0.22%); hepatitis C (0.39%)	During pregnancy	CCHD	Adjusted for maternal age, race and ethnicity, education, marital status, type of insurance, parity, time of prenatal care was initiated, prepregnancy BMI, prepregnancy diabetes, prepregnancy hypertension, gestational diabetes, gestational hypertension, hypertension eclampsia, history of preterm birth, history of cesarean section, use of fertility treatments, and weight gain during pregnancy.
Yan et al. (2022) [36]	China (Asia)	Cross-sectional	2014-2020	Hospital based	42,814 CHD cases and 5,028,985 non-CHD cases	Viral infection	During the perinatal period	CHD	NA
Mohammed et al. (2022) [37]	Iraq (Asia)	Case-control	Feb.1, 2022-May 1, 2022	Hospital based	50 CHD cases and 50 controls	Rubella infection (23%);†COVID-19 infection (8%)	During pregnancy	CHD	NA

Study	Study region	Study design	Study period	Study setting	Sample size	Investigated exposures	Exposure timing	Reported outcomes	Matching/adjusting variables
Ebeh et al. (2021) [38]	USA (North America)	Cross-sectional	2017	Population-based	2,130 CCHD cases and 3,859,817 non-CCHD cases	Gonorrhea infection (0.29%); syphilis infection (0.10%); chlamydia infection (1.83%);[†] hepatitis B infection (0.23%); hepatitis C infection (0.47%)	During pregnancy	CCHD	NA
Liang et al. (2017) [16]	China (Asia)	Cohort study	NA	Hospital based	145 CHD cases and 5,236 non-CHD cases	Mflu (28.4%);[†] mumps (0.2%); measles (0.2%); rubella (0.2%); chickenpox (0.3%); hepatitis (1.0%)	During pregnancy	CHD	NA
Dong et al. (2016) [17]	USA (North America)	Cohort study	2012	Population-based	2,487 CCHD cases and 3,334,424 controls	Chlamydia infection	During pregnancy	All CCHDs; isolated CCHDs	Adjusted for infant's sex, gestational age at delivery, birth weight; and maternal age at delivery, race/ethnicity, education, prepregnancy BMI, parity, periconceptional smoking status, initiation time of prenatal care, prepregnancy diabetes, prepregnancy hypertension, and use of assisted reproductive technology.
Liu et al. (2015) [47]	China (Asia)	Cross-sectional	July 2009-June 2012	Population-based	1,817 CHD cases and 88,979 non-CHD cases	Infection	During pregnancy	CHD	Adjusted for children's screening age, birthweight, gestational age, maternal age, pre-pregnancy BMI, pregnant infection, contact with toxic substance, using medicines, pregnancy-induced hypertension, gestational diabetes and anaemia during pregnancy, history of mother with CHD, history of father with CHD, education of mother, education of father, smoking, family income, health history of parents, genetic history of parents, drinking of parents, and decoration (home interior decoration) during pregnancy.

This table shows the characteristics of 9 studies investigating maternal infection at other/unspecific timepoints during pregnancy and risk of congenital heart defects in offspring. Additional study characteristics are presented in Table S3 (with the grey background). A total of 30 studies investigating maternal infection during the first trimester and risk of congenital heart defects in offspring are presented in Table 1 and Table S3 (without the grey background).

APVR: anomalous pulmonary venous return; AS: aortic stenosis; ASD: atrial septal defect; AVSD: atrioventricular septal defect; BMI: body mass index; CCHD: cyanotic congenital heart defects; CHD: congenital heart defects; CoA: coarctation of the aorta; DORV: double outlet right ventricle; EA: Ebstein's anomaly; HLHS: hypoplastic left heart syndrome; IAA: interrupted aortic arch; NA: not applicable; OR: odds ratio; PA: pulmonary atresia; PDA: patent ductus arteriosus; PS: pulmonary stenosis; TA: tricuspid atresia; TGA: transposition of the great arteries; TOF: tetralogy of Fallot; TS: truncus stenosis; VSD: ventricular septal defect.

[†] Variable with the highest prevalence and used as the quantitative summary for any infection in pooled analyses (in studies reporting associations for more than one infection).

Table S4. Detailed characteristics of 39 included studies of maternal infections and risk of congenital heart defects in offspring

This table provides details of the 39 studies included in this review, comprising

- 26 studies that investigated the association between maternal infections during the first trimester and overall congenital heart defects, and rated as low or moderate risk of bias (in blue, see also Table 1),
- 4 studies that investigated the association between maternal infections during the first trimester and subtype congenital heart defects, or rated as high risk of bias (in white, see also Table 1),
- 9 studies that investigated the association between maternal infections at other/unspecific timepoints during pregnancy and risk of congenital heart defects in offspring (included in sensitivity analyses, in grey, see also Table S2).

Detailed information for all 39 studies is available in the supplementary materials (xlsx file).

Table S5. Quality assessment of 39 included studies using the Newcastle-Ottawa Scale†

Case-control studies

	**Selection**				**Comparability ‡**	**Outcome**			
**Study**	Is the case definition adequate	Representativeness of the cases	Selection of controls	Definition of controls	Comparability of cases and controls on the basic of the design or analysis	Ascertainment of exposure	Same method of ascertainment for cases and controls	Non-response rate	**Total score**
Mátrai et al. (2023) [26]	★	★	★	★	★★	★	★		8
Mamun et al. (2023) [35]		★		★		★	★		4
Mohammed et al. (2022) [37]	★	★		★		★	★		5
Dolk et al. (2020) [39]	★	★	★	★	★★		★		7
Xia et al. (2019) [14]	★	★		★	★★		★		6
Lai et al. (2019) [40]	★	★		★	★★	★	★		7
Howley et al. (2018) [15]	★	★	★	★	★★	★	★		8
Feng et al. (2018) [42]	★	★		★	★★	★	★		7
Chen et al. (2016) [43]	★	★		★	★		★		5
Li et al. (2016) [44]	★	★		★	★	★	★		6
Ou et al. (2016) [45]	★		★	★	★★	★	★		7
Zou et al. (2015) [46]	★	★		★	★		★		5
Li et al. (2014) [48]	★	★		★	★★	★	★		7
Botto et al. (2014) [49]	★	★	★	★	★★	★	★		8
Taksande et al. (2013) [50]	★	★		★	★		★		5

Fung et al. (2013) [51]	★	★		★	★		★		5
Oster et al. (2011) [53]	★	★	★	★	★★		★		7
Liu et al. (2009) [18]	★	★		★	★		★		5
Acs et al. (2008) [29]		★	★	★	★★	★	★	★	8
Bánhidy et al. (2006) [27]		★	★	★	★	★	★	★	7
Acs et al. (2006) [28]		★	★	★	★★	★	★	★	8
Botto et al. (2001) [54]	★	★	★	★	★		★		6
Roguin et al. (1995) [55]	★	★		★			★		4
Tikkanen et al. (1991) [22]	★	★	★	★	★	★	★		7
Tikkanen et al. (1990) [23]	★	★	★	★	★★	★	★		8
Brown et al. (1972) [56]		★		★	★★	★	★		6

Cohort studies

	Selection				Comparability ‡	Outcome			
**Study**	Representativeness of the exposed cohort	Selection of the non-exposured cohort	Ascertainment of exposure	Demonstration that outcome of interest was not present at start analysis	Comparability of cohorts on the basis of the design or analysis	Assessment of outcome	Was follow-up long enough for outcomes to occur	Adequacy of follow up of cohort	**Total score**
Strzelecka et al. (2023) [57]		★		★		★			3
Chughtai et al. (2023) [11]	★	★	★	★	★★	★			7
Appiah et al. (2023) [12]	★	★		★	★★				5

Wang et al. (2022) [13]		★	★	★	★★	★	★	★	8
Liang et al. (2017) [16]		★		★					2
Dong et al. (2016) [17]	★	★		★	★★				5
Adams et al. (2012) [52]		★	★	★		★			4

Cross-sectional studies

Study	Selection			Comparability ‡	Outcome		Total score
	Representativeness of the sample	Non-respondents	Ascertainment of exposure	Comparability	Assessment of outcome	Statistical test	
Ruan et al. (2023) [24]	★			★	★	★	4
Yan et al. (2022) [36]					★		1
Ruan et al. (2021) [25]	★				★		2
Ebeh et al. (2021) [38]	★		★		★		3
Zhang et al. (2018) [41]	★			★	★	★	4
Liu et al. (2015) [47]	★			★★	★	★	5

† One star is awarded if the study meets corresponding assessment item within the Selection and Exposure categories.

‡ A maximum of two stars can be given for Comparability category, with maternal age being the most important confounder (i.e., the study adjusted for maternal age is awarded one star, plus other confounders is awarded two stars).

Table S6. Sensitivity analysis of any maternal infection for overall congenital heart defects in offspring

	Number of studies	Odds ratio (95% CI)	I^2
**Main analysis: studies with low or moderate risk of bias that investigated first-trimester maternal infection and overall congenital heart defects in offspring †**	**26**	**1.63 (1.41, 1.88)**	**76.36%**
Excluding studies did not adjust for or match on any covariate	25	1.64 (1.40, 1.91)	76.95%
Excluding studies investigating maternal infections more than 1 month before pregnancy	23	1.69 (1.43, 1.99)	77.37%
Excluding studies investigating maternal infections before pregnancy	21	1.62 (1.38, 1.91)	77.07%
Including studies investigating maternal infections occurring at timepoints other than 1st trimester (i.e., later or at unspecified time-points during pregnancy)	31	1.86 (1.43, 2.42)	96.12%
Including high risk of bias studies that investigated maternal infections during 1st trimester †	27	1.59 (1.37, 1.84)	83.01%
Including high risk of bias studies that investigated maternal infections other than 1st trimester †	36	1.64 (1.30, 2.07)	97.99%
Post-hoc: excluding studies reporting cyanotic congenital heart defects from studies at high risk of bias investigating maternal infections other than 1st trimester †	33	1.71 (1.32, 2.23)	98.03%
Post-hoc: including studies with fewer than 50 cases of congenital heart defects	29	1.66 (1.43, 1.91)	75.49%
Post-hoc: only including studies rated as low or moderate risk of bias according to the Risk Of Bias In Non-randomized Studies - of Exposures tool	25	1.64 (1.40, 1.91)	76.95%

This table presents the results of various sensitivity analyses examining the association between any maternal infection and overall congenital heart defects in offspring.

† Risk of bias was assessed using the Newcastle-Ottawa Scale. Scores of 0-3 indicate a high risk of bias, scores of 4-6 indicate a moderate risk of bias, and scores of 7-9 indicate a low risk of bias.

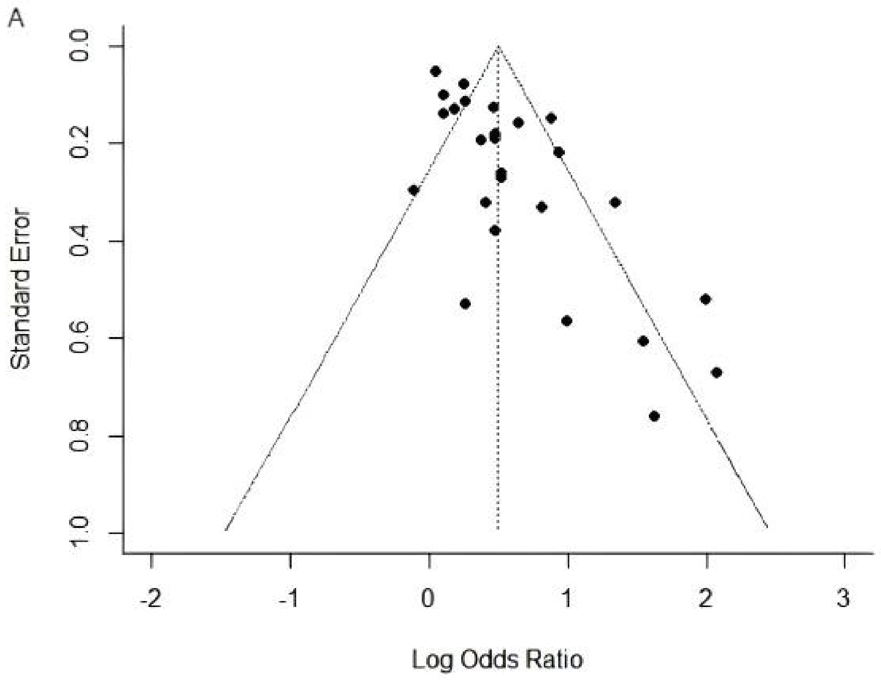

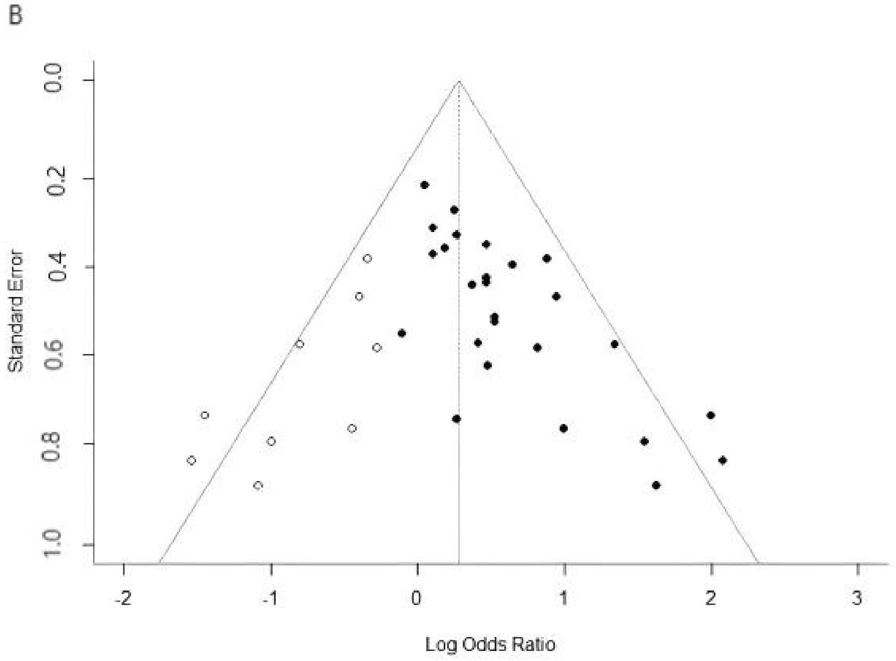

Observed OR (95% CI): 1.63 (1.41, 1.88)
Adjusted OR (95% CI) with imputed studies: 1.32 (1.12, 1.57)

Figure S1. Funnel plots of any first trimester maternal infection for overall congenital heart defects in offspring: A) observed studies; B) together with imputed studies

The plots are based on the 26 studies with low or moderate risk of bias that investigated first-trimester maternal infection and overall congenital heart defects in offspring.

Black dots: observed studies; white dots: imputed studies adjusted for publication bias.

Study	Type of infection	Infected cases/non-cases	Non-infected cases/non-cases	OR† (95% CI)	Weight	Quality score
**Ventricular septal defect**						
Mátrai 2023[26]	Influenza	79/1,750	2,783/ 84,964	1.38 [1.09; 1.72]	13.79%	8
Wang 2022[13]†	Hepatitis B virus	32/2,448	214/41,314	5.28 [3.66; 7.63]	11.08%	8
Xia 2019[14]	Upper respiratory tract infection/influenza	-	-	3.99 [1.94; 8.18]	5.80%	6
Howley 2018[15]†	Genitourinary infection	110/568	1,233/ 6,130	0.94 [0.75; 1.18]	13.82%	8
Feng 2018[42]	Influenza	-/109	-/2,344	1.20 [0.86; 1.67]	11.77%	7
Ou 2016[45]‡	Influenza	-	-	1.19 [0.71; 1.99]	8.45%	7
Botto 2014[49]†	Nonfebrile illness	168/1,022	843/6,746	1.20 [0.99; 1.45]	14.46%	8
Oster 2011[53]	Influenza	43/-	479/-	1.30 [0.92; 1.83]	11.54%	7
Botto 2001[54]‡	Influenza	15/114	172/2,704	2.00 [1.10; 3.60]	7.32%	6
Roguin 1995[56]‡	Urinary tract infeetion	2/43	54/932	0.80 [0.19; 3.40]	1.96%	4
**Random effects model**				**1.59 [1.16; 2.20]**	**100.00%**	
Heterogeneity: $I^2 = 88.13\%$, $\tau^2 = 0.21$, $\chi^2_9 = 75.85$ ($p < 0.001$)						
**Atrial septal defect**						
Mátrai 2023[26]	Influenza	28/1,801	1,865/85,882	0.72 [0.48; 1.02]	16.76%	8
Wang 2022[13]‡	Hepatitis B virus	4/2,476	166/41,362	0.74 [0.28; 2.00]	5.73%	8
Howley 2018[15]‡	Genitourinary infection	332/1,014	2,611/10,531	1.19 [1.04; 1.37]	23.53%	8
Feng 2018[42]	Influenza	-/109	-/2,344	1.12 [0.68; 1.85]	13.38%	7
Ou 2016[45]‡	Influenza	-	-	2.72 [1.17; 6.33]	7.19%	7
Botto 2014[49]‡	Nonfebrile illness	145/1,022	829/6,746	1.01 [0.83; 1.24]	21.99%	8
Oster 2011[53]	Influenza	10/-	186/-	0.73 [0.38; 1.42]	9.96%	7
Botto 2001[54]‡	Influenza	1/114	24/2,704	1.00 [0.10; 7.40]	1.46%	6
**Random effects model**				**1.03 [0.84; 1.26]**	**100.00%**	
Heterogeneity: $I^2 = 47.39\%$, $\tau^2 = 0.03$, $\chi^2_7 = 13.31$ ($p = 0.065$)						
**Tetralogy of Fallot**						
Wang 2022[13]‡	Hepatitis B virus	2/2,478	34/41,494	2.24 [0.53; 9.47]	4.25%	8
Xia 2019[14]	Upper respiratory tract infection/influenza	-	-	2.93 [1.56; 5.52]	14.67%	6
Howley 2018[15]‡	Genitourinary infection	105/1,014	1,057/10,531	1.09 [0.88; 1.36]	30.15%	8
Feng 2018[42]	Influenza	-/109	-/2,344	1.30 [0.43; 3.94]	6.65%	7
Botto 2014[49]†	Nonfebrile illness	102/1,022	561/6,746	1.15 [0.90; 1.45]	29.31%	8
Oster 2011[53]	Influenza	18/-	133/-	0.78 [0.37; 1.62]	12.09%	7
Botto 2001[54]‡	Influenza	1/114	48/2,704	0.50 [0.10; 3.60]	2.88%	6
**Random effects model**				**1.24 [0.94; 1.65]**	**100.00%**	
Heterogeneity: $I^2 = 47.24\%$, $\tau^2 = 0.05$, $\chi^2_6 = 11.37$ ($p = 0.078$)						
**Pulmonary stenosis**						
Wang 2022[13]‡	Hepatitis B virus	2/2,478	42/41,486	0.78 [0.18; 3.43]	4.56%	8
Howley 2018[15]‡	Genitourinary infection	151/1,014	1,375/10,531	1.13 [0.93; 1.37]	34.65%	8
Feng 2018[42]	Influenza	-/109	-/2,344	1.24 [0.39; 3.92]	6.93%	7
Ou 2016[45]‡	Influenza	-	-	0.62 [0.07; 5.66]	2.19%	7
Botto 2014[49]‡	Nonfebrile illness	80/1,022	542/6,746	0.92 [0.71; 1.19]	31.79%	8
Oster 2011[53]	Influenza	17/-	205/-	1.21 [0.71; 2.04]	19.86%	7
**Random effects model**				**1.06 [0.91; 1.23]**	**100.00%**	
Heterogeneity: $I^2 = 0\%$, $\tau^2 = 0$, $\chi^2_5 = 2.28$ ($p = 0.809$)						
**Transposition of the great arteries**						
Wang 2022[13]‡	Hepatitis B virus	0/2,480	28/41,500	0.29 [0.02; 4.81]	1.49%	8
Howley 2018[15]†	Genitourinary infection	68/1,014	683/10,531	0.99 [0.75; 1.31]	33.19%	8
Ou 2016[45]‡	Influenza	-	-	1.15 [0.07; 19.55]	1.47%	7
Botto 2014[49]‡	Nonfebrile illness	58/1,022	318/6,746	1.07 [0.79; 1.46]	31.75%	8
Oster 2011[53]	Influenza	18/-	185/-	1.21 [0.73; 2.02]	22.16%	7
Botto 2001[54]‡	Influenza	5/114	58/2,704	2.10 [0.80; 5.50]	9.94%	6
**Random effects model**				**1.07 [0.89; 1.29]**	**100.00%**	
Heterogeneity: $I^2 = 0\%$, $\tau^2 = 0$, $\chi^2_5 = 3.23$ ($p = 0.665$)						
**Anomalous pulmonary venous return**						
Howley 2018[15]‡	Genitourinary infection	17/1,014	277/10,531	0.62 [0.37; 1.04]	33.34%	8
Li 2014[46]	Influenza	8/104	30/312	0.68 [0.28; 1.65]	17.19%	7
Botto 2014[49]‡	Nonfebrile illness	8/1,022	148/6,746	0.85 [0.52; 1.41]	34.48%	8
Oster 2011[53]	Influenza	3/-	41/-	0.83 [0.25; 2.73]	10.75%	7
Botto 2001[54]‡	Influenza	1/114	11/2,704	2.20 [0.30; 16.90]	4.24%	6
**Random effects model**				**0.75 [0.55; 1.03]**	**100.00%**	
Heterogeneity: $I^2 = 0\%$, $\tau^2 = 0$, $\chi^2_4 = 1.93$ ($p = 0.748$)						
**Atrioventricular septal defect**						
Wang 2022[13]‡	Hepatitis B virus	0/2,480	52/41,476	0.16 [0.01; 2.58]	1.82%	8
Howley 2018[15]‡	Genitourinary infection	52/1,014	299/10,531	1.82 [1.33; 2.49]	37.93%	8
Botto 2014[49]†	Nonfebrile illness	22/1,022	87/6,746	1.46 [0.88; 2.43]	26.80%	8
Oster 2011[53]	Influenza	24/-	244/-	1.29 [0.82; 2.01]	29.93%	7
Botto 2001[54]†	Influenza	1/114	12/2,704	2.00 [0.30; 15.30]	3.51%	6
**Random effects model**				**1.55 [1.21; 1.99]**	**100.00%**	
Heterogeneity: $I^2 = 7.85\%$, $\tau^2 = < 0.01$, $\chi^2_4 = 4.34$ ($p = 0.362$)						
**Hypoplastic left heart syndrome**						
Howley 2018[15]‡	Genitourinary infection	68/1,014	568/10,531	1.26 [0.96; 1.65]	32.84%	8
Botto 2014[49]‡	Nonfebrile illness	49/1,022	301/6,746	0.96 [0.69; 1.33]	29.98%	8
Oster 2011[53]	Influenza	13/-	117/-	1.57 [0.86; 2.88]	18.12%	7
Botto 2001[54]‡	Influenza	2/114	30/2,704	1.60 [0.40; 6.70]	5.20%	6
Tikkanen 1990[23]	Respiratory infection	-	-	2.50 [1.20; 5.40]	13.86%	8
**Random effects model**				**1.30 [0.98; 1.72]**	**100.00%**	
Heterogeneity: $I^2 = 37.49\%$, $\tau^2 = 0.04$, $\chi^2_4 = 6.4$ ($p = 0.171$)						
**Aortic stenosis**						
Howley 2018[15]‡	Genitourinary infection	36/1,014	463/10,531	0.79 [0.54; 1.14]	39.67%	8
Botto 2014[49]‡‡	Nonfebrile illness	26/1,022	162/6,746	1.06 [0.68; 1.66]	34.80%	8
Oster 2011[53]	Influenza	8/-	54/-	1.53 [0.70; 3.34]	18.83%	7
Botto 2001[54]†	Influenza	2/114	12/2,704	4.00 [0.90; 17.90]	6.70%	6
**Random effects model**				**1.12 [0.73; 1.73]**	**100.00%**	
Heterogeneity: $I^2 = 50.28\%$, $\tau^2 = 0.09$, $\chi^2_3 = 6.03$ ($p = 0.110$)						
**Coarctation of the aorta**						
Howley 2018[15]‡	Genitourinary infection	101/1,014	1,036/10,531	1.08 [0.86; 1.35]	41.51%	8
Botto 2014[49]‡	Nonfebrile illness	53/1,022	309/6,746	1.10 [0.80; 1.51]	36.14%	8
Oster 2011[53]	Influenza	3/-	79/-	0.41 [0.13; 1.33]	8.54%	7
Botto 2001[54]‡	Influenza	7/114	42/2,704	3.80 [1.60; 8.80]	13.81%	6
**Random effects model**				**1.21 [0.77; 1.89]**	**100.00%**	
Heterogeneity: $I^2 = 72.41\%$, $\tau^2 = 0.13$, $\chi^2_3 = 10.87$ ($p = 0.012$)						
**Patent ductus arteriosus**						
Mátrai 2023[26]	Influenza	12/1,817	689/87,058	0.83 [0.44; 1.41]	45.87%	8
Wang 2022[13]‡	Hepatitis B virus	6/2,474	78/41,450	1.74 [0.76; 3.98]	29.52%	8
Feng 2018[42]	Influenza	-/109	-/2,344	1.38 [0.54; 3.52]	24.62%	7
**Random effects model**				**1.14 [0.72; 1.81]**	**100.00%**	
Heterogeneity: $I^2 = 12.88\%$, $\tau^2 = 0.02$, $\chi^2_2 = 2.3$ ($p = 0.317$)						
**Pulmonary atresia**						
Howley 2018[15]‡	Genitourinary infection	23/1,014	232/10,531	0.97 [0.62; 1.54]	44.30%	8
Botto 2014[49]†	Nonfebrile illness	14/1,022	88/6,746	0.83 [0.46; 1.52]	33.96%	8
Oster 2011[53]	Influenza	7/-	39/-	2.71 [1.16; 6.32]	21.74%	7
**Random effects model**				**1.19 [0.66; 2.15]**	**100.00%**	
Heterogeneity: $I^2 = 63.53\%$, $\tau^2 = 0.17$, $\chi^2_2 = 5.48$ ($p = 0.064$)						
**Tricuspid atresia**						
Howley 2018[15]†	Genitourinary infection	13/1,014	156/10,531	0.79 [0.42; 1.47]	58.70%	8
Oster 2011[53]	Influenza	7/-	26/-	6.04 [2.36; 15.42]	34.13%	7
Botto 2001[54]‡	Influenza	3/114	9/2,704	7.90 [0.30; 29.60]	7.17%	6
**Random effects model**				**2.88 [0.54; 15.36]**	**100.00%**	
Heterogeneity: $I^2 = 86.14\%$, $\tau^2 = 1.74$, $\chi^2_2 = 14.43$ ($p < 0.001$)						
**Ebstein's anomaly**						
Howley 2018[15]‡	Genitourinary infection	19/1,014	160/10,531	1.23 [0.75; 2.02]	89.37%	8
Botto 2001[54]†	Influenza	1/114	8/2,704	3.00 [0.40; 23.90]	10.63%	6
**Random effects model**				**1.29 [0.80; 2.09]**	**100.00%**	
Heterogeneity: $I^2 = 0\%$, $\tau^2 = 0$, $\chi^2_1 = 0.69$ ($p = 0.406$)						

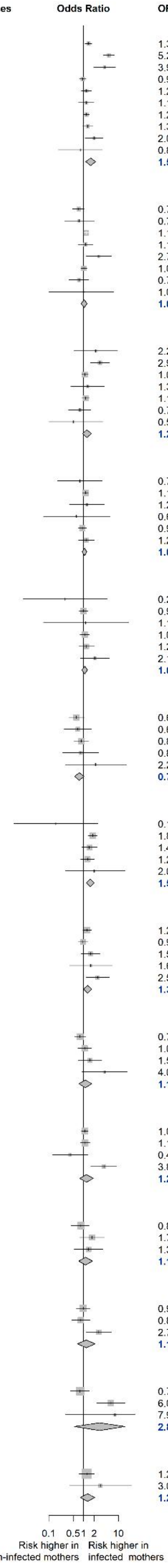

Figure S2. Meta-analysis of any first trimester maternal infection for congenital heart defects in offspring, by specific type of heart defects

Forest plot presenting the association between any maternal infection during the first trimester of pregnancy and risk of specific type of congenital heart defects in the offspring. Pooled OR and their corresponding 95% CI were calculated using inverse variance weighted random-effects model. The quality score was calculated using the Newcastle-Ottawa Scale. Scores of 0-3, 4-6, and 7-9 are regarded as high, moderate, and low risk of bias, respectively. Main analyses were restricted to studies with low or moderate risk of bias. OR = odds ratio. CI = confidence interval.

[†]: Wang 2022 reports relative risk, others report odds ratio.

[‡]: In studies reporting associations for more than one infection, the infection variable with the highest prevalence ("Type of infection" column) was used as the quantitative summary for any infection in pooled analyses.

A. By study region

Study	Type of infection	Infected cases/non-cases	Non-infected cases/non-cases	OR† (95% CI)	Weight	Quality score
**Asia**						
Ruan 2023[24]	Respiratory infection	168/609	707/3,540	1.30 [1.04, 1.61]	5.90%	4
Wang 2022[13 ‡]	Hepatitis B virus	50/2,430	514/41,014	2.41 [1.80, 3.21]	5.36%	8
Xia 2019[14]	Respiratory infection/influenza	-	-	2.56 [1.67, 3.92]	4.28%	6
Lai 2019[40 ‡]	Influenza	116/55	1,120/861	1.45 [0.99, 2.12]	4.63%	7
Zhang 2018[41]	Viral infection	84/23,990	64/22,837	3.81 [1.96, 6.92]	2.96%	4
Feng 2018[42]	Influenza	114/109	2,344/2,349	1.11 [0.84, 1.45]	5.49%	7
Chen 2016[43]	Viral infection	36/16	399/558	2.26 [1.18, 4.34]	2.86%	5
Li 2016[44 ‡]	Influenza	25/6	25/44	7.33 [2.65, 20.28]	1.56%	6
Ou 2016[45 ‡]	Influenza	243/-	3,613/-	1.60 [1.10, 2.32]	4.69%	7
Zou 2015[46]	Viral infection	-	-	5.08 [1.14, 22.57]	0.83%	5
Li 2014[48]	Influenza	103/104	191/312	1.60 [1.12, 2.28]	4.83%	7
Taksande 2013[50]	Viral infection	9/4	200/414	4.66 [1.42, 15.31]	1.21%	5
Liu 2009[18 ‡]	Infection	50/7	114/321	7.98 [2.14, 29.72]	1.03%	5
**Random effects model**				**2.12 [1.63, 2.75]**	**45.63%**	
Heterogeneity: I^2 = 76.15%, τ^2 = 0.14, χ^2_{12} = 50.31 (p < 0.001)						
Test for effect in subgroup: z = 5.62 (p < 0.001)						
**Europe**						
Mátrai 2023[26]	Influenza	190/1,639	7,296/80,451	1.28 [1.10, 1.49]	6.35%	8
Dolk 2020[39 ‡]	Vaginal infection	30/74	212/892	1.69 [1.01, 2.80]	3.69%	7
Acs 2008[29]	Recurrent genital herpes	7/26	4,472/38,125	1.30 [0.50, 4.00]	1.51%	8
Bánhidy 2006[27]	Urinary tract infection	61/514	4,418/37,637	0.90 [0.50, 1.60]	3.24%	7
Acs 2006[28]	Respiratory infection	183/1,221	4,296/36,930	1.20 [0.90, 1.50]	5.63%	8
Tikkanen 1991[22]	Respiratory infection	147/189	419/861	1.60 [1.25, 2.04]	5.70%	7
**Random effects model**				**1.33 [1.17, 1.50]**	**26.12%**	
Heterogeneity: I^2 = 10.57%, τ^2 = < 0.01, χ^2_5 = 5.59 (p = 0.348)						
Test for effect in subgroup: z = 4.44 (p < 0.001)						
**Oceania**						
Chughtai 2023[11 ‡]	Respiratory infection	14/1,533	7,671/1,433,656	1.68 [0.99, 2.84]	3.58%	7
**North America**						
Botto 2014[49 ‡]	Nonfebrile illness	1,039/1,022	5,981/6,746	1.05 [0.95, 1.16]	6.61%	8
Fung 2013[51 ‡]	Infection	190/14	1,369/141	1.50 [0.80, 2.80]	2.99%	5
Adams 2012[52]	Viral infection	4/20	62/833	2.69 [0.89, 8.11]	1.37%	4
Oster 2011[53]	Influenza	193/-	2,361/-	1.11 [0.91, 1.35]	6.06%	7
Botto 2001[54 ‡]	Respiratory infection	75/143	736/2,704	1.90 [1.40, 2.60]	5.20%	6
Brown 1972[56 ‡]	Coxsackie B4	14/17	125/244	1.61 [0.77, 3.37]	2.45%	6
**Random effects model**				**1.35 [1.06, 1.72]**	**24.67%**	
Heterogeneity: I^2 = 70.51%, τ^2 = 0.05, χ^2_5 = 16.96 (p = 0.005)						
Test for effect in subgroup: z = 2.41 (p = 0.016)						
**Random effects model**				**1.63 [1.41, 1.88]**	**100.00%**	
Heterogeneity: I^2 = 76.36%, τ^2 = 0.08, χ^2_{25} = 105.74 (p < 0.001)						
Test for overall effect: z = 6.63 (p < 0.001)						
Test for subgroup differences: χ^2_3 = 10.65, df = 3 (p = 0.014)						

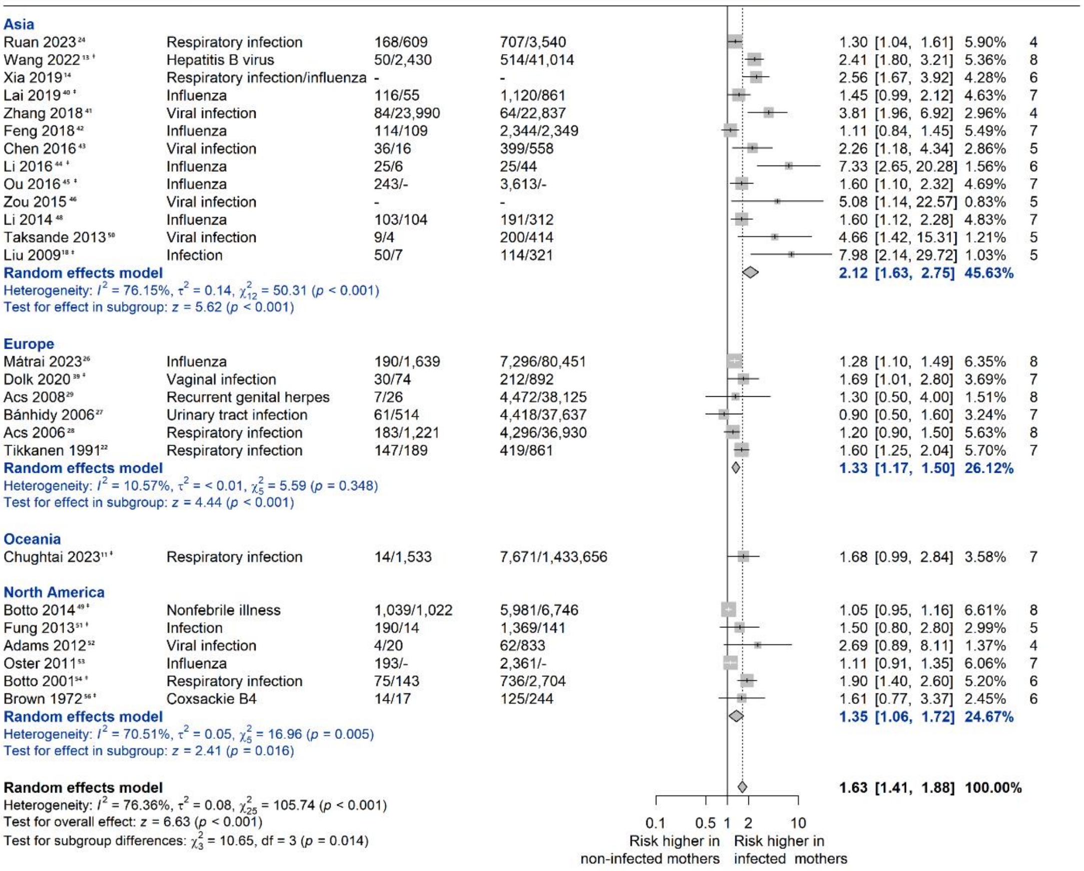

B. By study design

Study	Type of infection	Infected cases/non-cases	Non-infected cases/non-cases	OR† (95% CI)	Weight	Quality score
**Cross-sectional**						
Ruan 2023[24]	Respiratory infection	168/609	707/3,540	1.30 [1.04, 1.61]	5.90%	4
Zhang 2018[41]	Viral infection	84/23,990	64/22,837	3.81 [1.96, 6.92]	2.96%	4
**Random effects model**				**2.13 [0.74, 6.12]**	**8.87%**	
Heterogeneity: I^2 = 90%, τ^2 = 0.52, χ^2_1 = 10 (p = 0.002)						
Test for effect in subgroup: z = 1.41 (p = 0.159)						
**Case-control**						
Mátrai 2023[26]	Influenza	190/1,639	7,296/80,451	1.28 [1.10, 1.49]	6.35%	8
Dolk 2020[39 ‡]	Vaginal infection	30/74	212/892	1.69 [1.01, 2.80]	3.69%	7
Xia 2019[14]	Respiratory infection/influenza	-	-	2.56 [1.67, 3.92]	4.28%	6
Lai 2019[40 ‡]	Influenza	116/55	1,120/861	1.45 [0.99, 2.12]	4.63%	7
Feng 2018[42]	Influenza	114/109	2,344/2,349	1.11 [0.84, 1.45]	5.49%	7
Chen 2016[43]	Viral infection	36/16	399/558	2.26 [1.18, 4.34]	2.86%	5
Li 2016[44 ‡]	Influenza	25/6	25/44	7.33 [2.65, 20.28]	1.56%	6
Ou 2016[45 ‡]	Influenza	243/-	3,613/-	1.60 [1.10, 2.32]	4.69%	7
Zou 2015[46]	Viral infection	-	-	5.08 [1.14, 22.57]	0.83%	5
Li 2014[48]	Influenza	103/104	191/312	1.60 [1.12, 2.28]	4.83%	7
Botto 2014[49 ‡]	Nonfebrile illness	1,039/1,022	5,981/6,746	1.05 [0.95, 1.16]	6.61%	8
Taksande 2013[50]	Viral infection	9/4	200/414	4.66 [1.42, 15.31]	1.21%	5
Fung 2013[51 ‡]	Infection	190/14	1,369/141	1.50 [0.80, 2.80]	2.99%	5
Oster 2011[53]	Influenza	193/-	2,361/-	1.11 [0.91, 1.35]	6.06%	7
Liu 2009[18 ‡]	Infection	50/7	114/321	7.98 [2.14, 29.72]	1.03%	5
Acs 2008[29]	Recurrent genital herpes	7/26	4,472/38,125	1.30 [0.50, 4.00]	1.51%	8
Bánhidy 2006[27]	Urinary tract infection	61/514	4,418/37,637	0.90 [0.50, 1.60]	3.24%	7
Acs 2006[28]	Respiratory infection	183/1,221	4,296/36,930	1.20 [0.90, 1.50]	5.63%	8
Botto 2001[54 ‡]	Respiratory infection	75/143	736/2,704	1.90 [1.40, 2.60]	5.20%	6
Tikkanen 1991[22]	Respiratory infection	147/189	419/861	1.60 [1.25, 2.04]	5.70%	7
Brown 1972[56 ‡]	Coxsackie B4	14/17	125/244	1.61 [0.77, 3.37]	2.45%	6
**Random effects model**				**1.53 [1.32, 1.78]**	**80.82%**	
Heterogeneity: I^2 = 72.85%, τ^2 = 0.06, χ^2_{20} = 73.66 (p < 0.001)						
Test for effect in subgroup: z = 5.60 (p < 0.001)						
**Cohort study**						
Chughtai 2023[11 ‡]	Respiratory infection	14/1,533	7,671/1,433,656	1.68 [0.99, 2.84]	3.58%	7
Wang 2022[13 ‡]	Hepatitis B virus	50/2,430	514/41,014	2.41 [1.80, 3.21]	5.36%	8
Adams 2012[52]	Viral infection	4/20	62/833	2.69 [0.89, 8.11]	1.37%	4
**Random effects model**				**2.24 [1.75, 2.87]**	**10.31%**	
Heterogeneity: I^2 = 0%, τ^2 = 0, χ^2_2 = 1.5 (p = 0.474)						
Test for effect in subgroup: z = 6.39 (p < 0.001)						
**Random effects model**				**1.63 [1.41, 1.88]**	**100.00%**	
Heterogeneity: I^2 = 76.36%, τ^2 = 0.08, χ^2_{25} = 105.74 (p < 0.001)						
Test for overall effect: z = 6.63 (p < 0.001)						
Test for subgroup differences: χ^2_2 = 6.85, df = 2 (p = 0.033)						

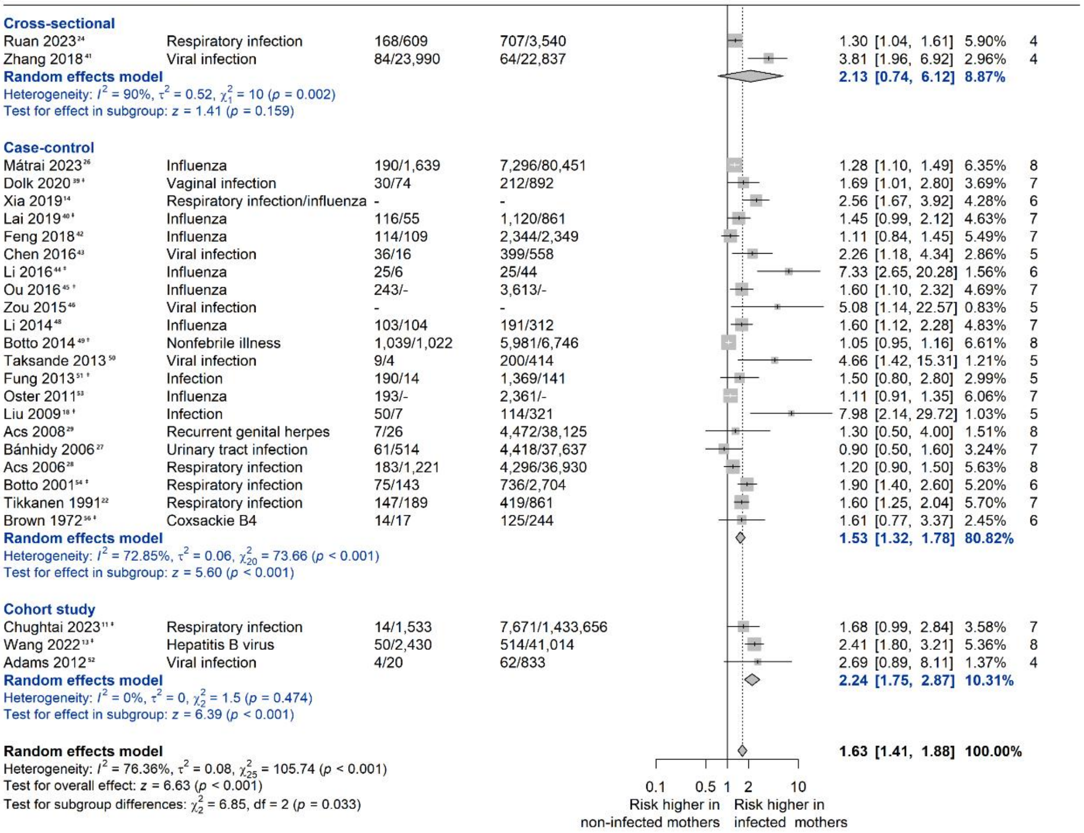

C. By study setting

Study	Type of infection	Infected cases/non-cases	Non-infected cases/non-cases	OR† (95% CI)	Weight	Quality score
**Population-based**						
Ruan 2023[24]	Respiratory infection	168/609	707/3,540	1.30 [1.04, 1.61]	5.90%	4
Mátrai 2023[26]	Influenza	190/1,639	7,296/80,451	1.28 [1.10, 1.49]	6.35%	8
Chughtai 2023[11‡]	Respiratory infection	14/1,533	7,671/1,433,656	1.68 [0.99, 2.84]	3.58%	7
Dolk 2020[39‡]	Vaginal infection	30/74	212/892	1.69 [1.01, 2.80]	3.69%	7
Zhang 2018[41]	Viral infection	84/23,990	64/22,837	3.81 [1.96, 6.92]	2.96%	4
Ou 2016[45‡]	Influenza	243/-	3,613/-	1.60 [1.10, 2.32]	4.69%	7
Botto 2014[49‡]	Nonfebrile illness	1,039/1,022	5,981/6,746	1.05 [0.95, 1.16]	6.61%	8
Oster 2011[53]	Influenza	193/-	2,361/-	1.11 [0.91, 1.35]	6.06%	7
Acs 2008[29]	Recurrent genital herpes	7/26	4,472/38,125	1.30 [0.50, 4.00]	1.51%	8
Bánhidy 2006[27]	Urinary tract infection	61/514	4,418/37,637	0.90 [0.50, 1.60]	3.24%	7
Acs 2006[28]	Respiratory infection	183/1,221	4,296/36,930	1.20 [0.90, 1.50]	5.63%	8
Botto 2001[54†]	Respiratory infection	75/143	736/2,704	1.90 [1.40, 2.60]	5.20%	6
Tikkanen 1991[22]	Respiratory infection	147/189	419/861	1.60 [1.25, 2.04]	5.70%	7
**Random effects model**				**1.38 [1.20, 1.59]**	**61.11%**	
Heterogeneity: $I^2 = 70.99\%$, $\tau^2 = 0.04$, $\chi^2_{12} = 41.36$ ($p < 0.001$)						
Test for effect in subgroup: $z = 4.47$ ($p < 0.001$)						
**Hospital based**						
Wang 2022[13‡]	Hepatitis B virus	50/2,430	514/41,014	2.41 [1.80, 3.21]	5.36%	8
Xia 2019[14]	Respiratory infection/influenza	-	-	2.56 [1.67, 3.92]	4.28%	6
Lai 2019[40‡]	Influenza	116/55	1,120/861	1.45 [0.99, 2.12]	4.63%	7
Feng 2018[42]	Influenza	114/109	2,344/2,349	1.11 [0.84, 1.45]	5.49%	7
Chen 2016[43]	Viral infection	36/16	399/558	2.26 [1.18, 4.34]	2.86%	5
Li 2016[44‡]	Influenza	25/6	25/44	7.33 [2.65, 20.28]	1.56%	6
Zou 2015[46]	Viral infection	-	-	5.08 [1.14, 22.57]	0.83%	5
Li 2014[48]	Influenza	103/104	191/312	1.60 [1.12, 2.28]	4.83%	7
Taksande 2013[50]	Viral infection	9/4	200/414	4.66 [1.42, 15.31]	1.21%	5
Fung 2013[51‡]	Infection	190/14	1,369/141	1.50 [0.80, 2.80]	2.99%	5
Adams 2012[52]	Viral infection	4/20	62/833	2.69 [0.89, 8.11]	1.37%	4
Liu 2009[18‡]	Infection	50/7	114/321	7.98 [2.14, 29.72]	1.03%	5
Brown 1972[56‡]	Coxsackie B4	14/17	125/244	1.61 [0.77, 3.37]	2.45%	6
**Random effects model**				**2.14 [1.62, 2.83]**	**38.89%**	
Heterogeneity: $I^2 = 68.58\%$, $\tau^2 = 0.14$, $\chi^2_{12} = 38.19$ ($p < 0.001$)						
Test for effect in subgroup: $z = 5.36$ ($p < 0.001$)						
**Random effects model**				**1.63 [1.41, 1.88]**	**100.00%**	
Heterogeneity: $I^2 = 76.36\%$, $\tau^2 = 0.08$, $\chi^2_{25} = 105.74$ ($p < 0.001$)						
Test for overall effect: $z = 6.63$ ($p < 0.001$)						
Test for subgroup differences: $\chi^2_1 = 7.51$, df = 1 ($p = 0.006$)						

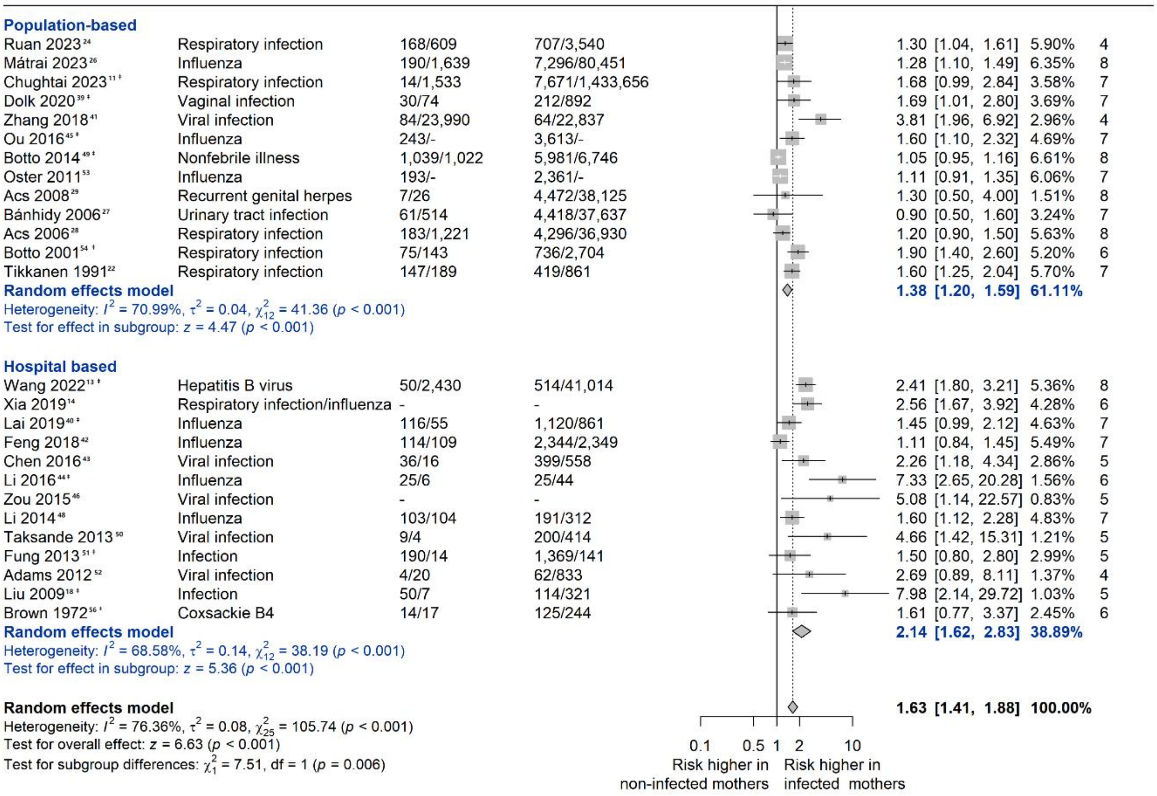

D. By infection assessment method

Study	Type of infection	Infected cases/non-cases	Non-infected cases/non-cases	OR† (95% CI)	Weight	Quality score
**Self-report**						
Ruan 2023[24]	Respiratory infection	168/609	707/3,540	1.30 [1.04, 1.61]	5.90%	4
Dolk 2020[39‡]	Vaginal infection	30/74	212/892	1.69 [1.01, 2.80]	3.69%	7
Xia 2019[14]	Respiratory infection/influenza	-	-	2.56 [1.67, 3.92]	4.28%	6
Zhang 2018[41]	Viral infection	84/23,990	64/22,837	3.81 [1.96, 6.92]	2.96%	4
Feng 2018[42]	Influenza	114/109	2,344/2,349	1.11 [0.84, 1.45]	5.49%	7
Chen 2016[43]	Viral infection	36/16	399/558	2.26 [1.18, 4.34]	2.86%	5
Li 2016[44‡]	Influenza	25/6	25/44	7.33 [2.65, 20.28]	1.56%	6
Ou 2016[45‡]	Influenza	243/-	3,613/-	1.60 [1.10, 2.32]	4.69%	7
Zou 2015[46]	Viral infection	-	-	5.08 [1.14, 22.57]	0.83%	5
Li 2014[48]	Influenza	103/104	191/312	1.60 [1.12, 2.28]	4.83%	7
Botto 2014[49‡]	Nonfebrile illness	1,039/1,022	5,981/6,746	1.05 [0.95, 1.16]	6.61%	8
Taksande 2013[50]	Viral infection	9/4	200/414	4.66 [1.42, 15.31]	1.21%	5
Fung 2013[51‡]	Infection	190/14	1,369/141	1.50 [0.80, 2.80]	2.99%	5
Oster 2011[53]	Influenza	193/-	2,361/-	1.11 [0.91, 1.35]	6.06%	7
Liu 2009[18‡]	Infection	50/7	114/321	7.98 [2.14, 29.72]	1.03%	5
Botto 2001[54‡]	Respiratory infection	75/143	736/2,704	1.90 [1.40, 2.60]	5.20%	6
Tikkanen 1991[22]	Respiratory infection	147/189	419/861	1.60 [1.25, 2.04]	5.70%	7
**Random effects model**				**1.75 [1.44, 2.13]**	**65.89%**	
Heterogeneity: $I^2 = 80.72\%$, $\tau^2 = 0.10$, $\chi^2_{16} = 82.97$ ($p < 0.001$)						
Test for effect in subgroup: $z = 5.63$ ($p < 0.001$)						
**Medical records**						
Mátrai 2023[26]	Influenza	190/1,639	7,296/80,451	1.28 [1.10, 1.49]	6.35%	8
Lai 2019[40‡]	Influenza	116/55	1,120/861	1.45 [0.99, 2.12]	4.63%	7
Acs 2008[29]	Recurrent genital herpes	7/26	4,472/38,125	1.30 [0.50, 4.00]	1.51%	8
Acs 2006[28]	Respiratory infection	183/1,221	4,296/36,930	1.20 [0.90, 1.50]	5.63%	8
**Random effects model**				**1.28 [1.13, 1.44]**	**18.11%**	
Heterogeneity: $I^2 = 0\%$, $\tau^2 = 0$, $\chi^2_3 = 0.66$ ($p = 0.883$)						
Test for effect in subgroup: $z = 3.92$ ($p < 0.001$)						
**Laboratory testing**						
Chughtai 2023[11‡]	Respiratory infection	14/1,533	7,671/1,433,656	1.68 [0.99, 2.84]	3.58%	7
Wang 2022[13‡]	Hepatitis B virus	50/2,430	514/41,014	2.41 [1.80, 3.21]	5.36%	8
Adams 2012[52]	Viral infection	4/20	62/833	2.69 [0.89, 8.11]	1.37%	4
Bánhidy 2006[27]	Urinary tract infection	61/514	4,418/37,637	0.90 [0.50, 1.60]	3.24%	7
Brown 1972[56‡]	Coxsackie B4	14/17	125/244	1.61 [0.77, 3.37]	2.45%	6
**Random effects model**				**1.71 [1.15, 2.56]**	**16.00%**	
Heterogeneity: $I^2 = 58.83\%$, $\tau^2 = 0.11$, $\chi^2_4 = 9.72$ ($p = 0.046$)						
Test for effect in subgroup: $z = 2.65$ ($p = 0.008$)						
**Random effects model**				**1.63 [1.41, 1.88]**	**100.00%**	
Heterogeneity: $I^2 = 76.36\%$, $\tau^2 = 0.08$, $\chi^2_{25} = 105.74$ ($p < 0.001$)						
Test for overall effect: $z = 6.63$ ($p < 0.001$)						
Test for subgroup differences: $\chi^2_2 = 8.18$, df = 2 ($p = 0.017$)						

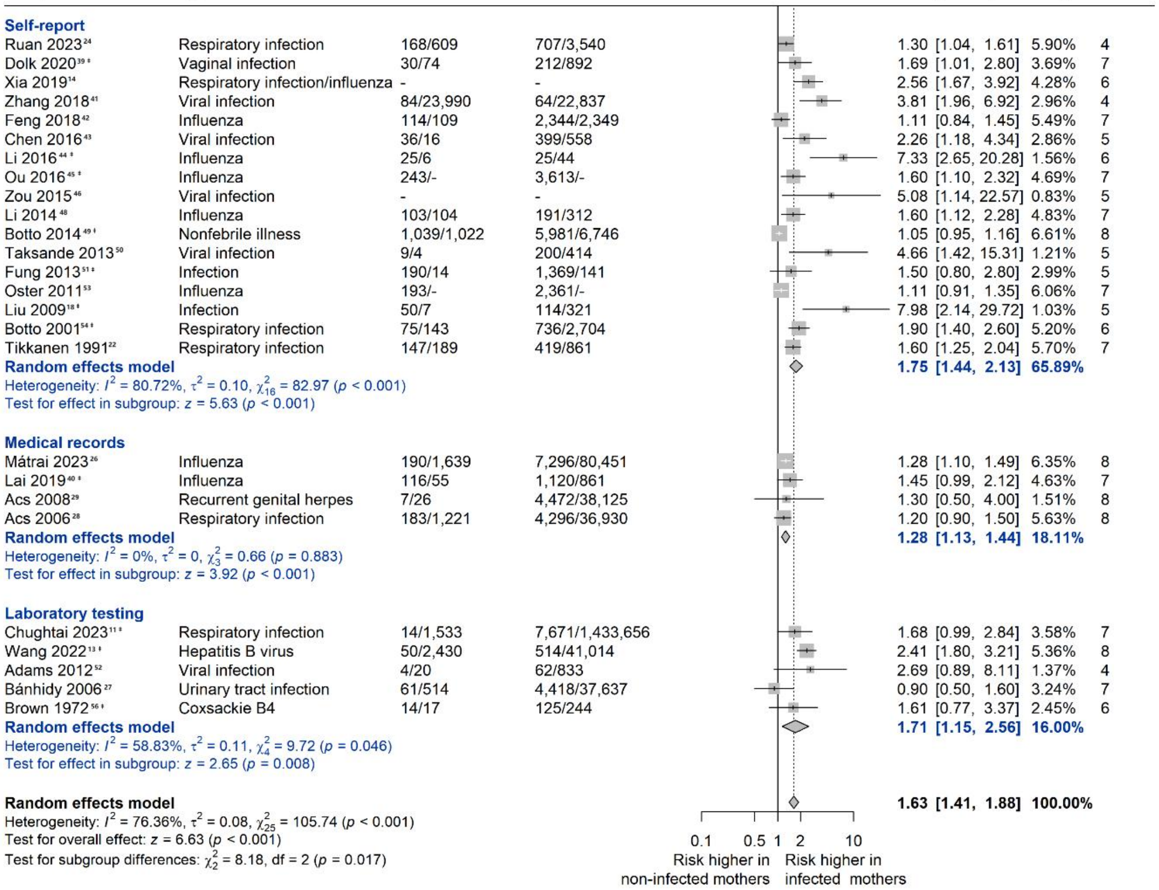

E. By adjustement for confounders or not

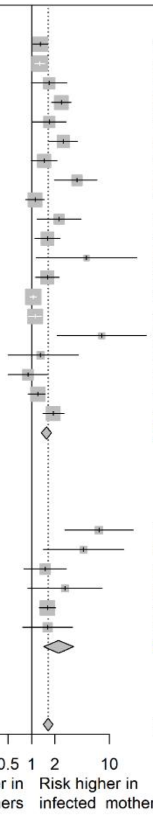

Study	Type of infection	Infected cases/non-cases	Non-infected cases/non-cases	OR† (95% CI)	Weight	Quality score
**Adjusted for confounders**						
Ruan 2023[24]	Respiratory infection	168/609	707/3,540	1.30 [1.04, 1.61]	5.90%	4
Mátrai 2023[26]	Influenza	190/1,639	7,296/80,451	1.28 [1.10, 1.49]	6.35%	8
Chughtai 2023[11] ‡	Respiratory infection	14/1,533	7,671/1,433,656	1.68 [0.99, 2.84]	3.58%	7
Wang 2022[13] ‡	Hepatitis B virus	50/2,430	514/41,014	2.41 [1.80, 3.21]	5.36%	8
Dolk 2020[39] ‡	Vaginal infection	30/74	212/892	1.69 [1.01, 2.80]	3.69%	7
Xia 2019[14]	Respiratory infection/influenza	-	-	2.56 [1.67, 3.92]	4.28%	6
Lai 2019[40] ‡	Influenza	116/55	1,120/861	1.45 [0.99, 2.12]	4.63%	7
Zhang 2018[41]	Viral infection	84/23,990	64/22,837	3.81 [1.96, 6.92]	2.96%	4
Feng 2018[42]	Influenza	114/109	2,344/2,349	1.11 [0.84, 1.45]	5.49%	7
Chen 2016[43]	Viral infection	36/16	399/558	2.26 [1.18, 4.34]	2.86%	5
Ou 2016[45] ‡	Influenza	243/-	3,613/-	1.60 [1.10, 2.32]	4.69%	7
Zou 2015[46]	Viral infection	-	-	5.08 [1.14, 22.57]	0.83%	5
Li 2014[48]	Influenza	103/104	191/312	1.60 [1.12, 2.28]	4.83%	7
Botto 2014[49] ‡	Nonfebrile illness	1,039/1,022	5,981/6,746	1.05 [0.95, 1.16]	6.61%	8
Oster 2011[53]	Influenza	193/-	2,361/-	1.11 [0.91, 1.35]	6.06%	7
Liu 2009[18] ‡	Infection	50/7	114/321	7.98 [2.14, 29.72]	1.03%	5
Acs 2008[29]	Recurrent genital herpes	7/26	4,472/38,125	1.30 [0.50, 4.00]	1.51%	8
Bánhidy 2006[27]	Urinary tract infection	61/514	4,418/37,637	0.90 [0.50, 1.60]	3.24%	7
Acs 2006[28]	Respiratory infection	183/1,221	4,296/36,930	1.20 [0.90, 1.50]	5.63%	8
Botto 2001[54] ‡	Respiratory infection	75/143	736/2,704	1.90 [1.40, 2.60]	5.20%	6
**Random effects model**				**1.55 [1.33, 1.81]**	**84.71%**	
Heterogeneity: $I^2 = 77.7\%$, $\tau^2 = 0.07$, $\chi^2_{19} = 85.21$ ($p < 0.001$)						
Test for effect in subgroup: $z = 5.66$ ($p < 0.001$)						
**Not adjusted for confounders**						
Li 2016[44] ‡	Influenza	25/6	25/44	7.33 [2.65, 20.28]	1.56%	6
Taksande 2013[50]	Viral infection	9/4	200/414	4.66 [1.42, 15.31]	1.21%	5
Fung 2013[51] ‡	Infection	190/14	1,369/141	1.50 [0.80, 2.80]	2.99%	5
Adams 2012[52]	Viral infection	4/20	62/833	2.69 [0.89, 8.11]	1.37%	4
Tikkanen 1991[22]	Respiratory infection	147/189	419/861	1.60 [1.25, 2.04]	5.70%	7
Brown 1972[56] ‡	Coxsackie B4	14/17	125/244	1.61 [0.77, 3.37]	2.45%	6
**Random effects model**				**2.23 [1.43, 3.47]**	**15.29%**	
Heterogeneity: $I^2 = 56.88\%$, $\tau^2 = 0.15$, $\chi^2_5 = 11.59$ ($p = 0.041$)						
Test for effect in subgroup: $z = 3.55$ ($p < 0.001$)						
**Random effects model**				**1.63 [1.41, 1.88]**	**100.00%**	
Heterogeneity: $I^2 = 76.36\%$, $\tau^2 = 0.08$, $\chi^2_{25} = 105.74$ ($p < 0.001$)						
Test for overall effect: $z = 6.63$ ($p < 0.001$)						
Test for subgroup differences: $\chi^2_1 = 2.31$, df = 1 ($p = 0.128$)						

F. By inclusion of pregnancy termination or not

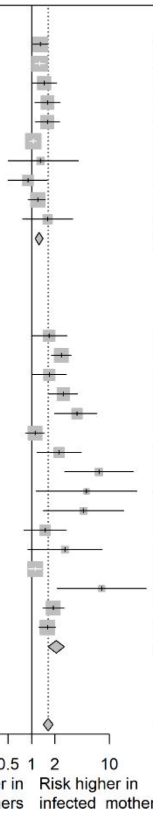

Study	Type of infection	Infected cases/non-cases	Non-infected cases/non-cases	OR† (95% CI)	Weight	Quality score
**Inclusion of pregnancy terminations**						
Ruan 2023[24]	Respiratory infection	168/609	707/3,540	1.30 [1.04, 1.61]	5.90%	4
Mátrai 2023[26]	Influenza	190/1,639	7,296/80,451	1.28 [1.10, 1.49]	6.35%	8
Lai 2019[40] ‡	Influenza	116/55	1,120/861	1.45 [0.99, 2.12]	4.63%	7
Ou 2016[45] ‡	Influenza	243/-	3,613/-	1.60 [1.10, 2.32]	4.69%	7
Li 2014[48]	Influenza	103/104	191/312	1.60 [1.12, 2.28]	4.83%	7
Botto 2014[49] ‡	Nonfebrile illness	1,039/1,022	5,981/6,746	1.05 [0.95, 1.16]	6.61%	8
Acs 2008[29]	Recurrent genital herpes	7/26	4,472/38,125	1.30 [0.50, 4.00]	1.51%	8
Bánhidy 2006[27]	Urinary tract infection	61/514	4,418/37,637	0.90 [0.50, 1.60]	3.24%	7
Acs 2006[28]	Respiratory infection	183/1,221	4,296/36,930	1.20 [0.90, 1.50]	5.63%	8
Brown 1972[56] ‡	Coxsackie B4	14/17	125/244	1.61 [0.77, 3.37]	2.45%	6
**Random effects model**				**1.25 [1.12, 1.41]**	**45.84%**	
Heterogeneity: $I^2 = 40.56\%$, $\tau^2 = 0.01$, $\chi^2_9 = 15.14$ ($p = 0.087$)						
Test for effect in subgroup: $z = 3.90$ ($p < 0.001$)						
**No inclusion of pregnancy terminations**						
Chughtai 2023[11] ‡	Respiratory infection	14/1,533	7,671/1,433,656	1.68 [0.99, 2.84]	3.58%	7
Wang 2022[13] ‡	Hepatitis B virus	50/2,430	514/41,014	2.41 [1.80, 3.21]	5.36%	8
Dolk 2020[39] ‡	Vaginal infection	30/74	212/892	1.69 [1.01, 2.80]	3.69%	7
Xia 2019[14]	Respiratory infection/influenza	-	-	2.56 [1.67, 3.92]	4.28%	6
Zhang 2018[41]	Viral infection	84/23,990	64/22,837	3.81 [1.96, 6.92]	2.96%	4
Feng 2018[42]	Influenza	114/109	2,344/2,349	1.11 [0.84, 1.45]	5.49%	7
Chen 2016[43]	Viral infection	36/16	399/558	2.26 [1.18, 4.34]	2.86%	5
Li 2016[44] ‡	Influenza	25/6	25/44	7.33 [2.65, 20.28]	1.56%	6
Zou 2015[46]	Viral infection	-	-	5.08 [1.14, 22.57]	0.83%	5
Taksande 2013[50]	Viral infection	9/4	200/414	4.66 [1.42, 15.31]	1.21%	5
Fung 2013[51] ‡	Infection	190/14	1,369/141	1.50 [0.80, 2.80]	2.99%	5
Adams 2012[52]	Viral infection	4/20	62/833	2.69 [0.89, 8.11]	1.37%	4
Oster 2011[53]	Influenza	193/-	2,361/-	1.11 [0.91, 1.35]	6.06%	7
Liu 2009[18] ‡	Infection	50/7	114/321	7.98 [2.14, 29.72]	1.03%	5
Botto 2001[54] ‡	Respiratory infection	75/143	736/2,704	1.90 [1.40, 2.60]	5.20%	6
Tikkanen 1991[22]	Respiratory infection	147/189	419/861	1.60 [1.25, 2.04]	5.70%	7
**Random effects model**				**2.08 [1.64, 2.63]**	**54.16%**	
Heterogeneity: $I^2 = 76.01\%$, $\tau^2 = 0.14$, $\chi^2_{15} = 62.52$ ($p < 0.001$)						
Test for effect in subgroup: $z = 6.03$ ($p < 0.001$)						
**Random effects model**				**1.63 [1.41, 1.88]**	**100.00%**	
Heterogeneity: $I^2 = 76.36\%$, $\tau^2 = 0.08$, $\chi^2_{25} = 105.74$ ($p < 0.001$)						
Test for overall effect: $z = 6.63$ ($p < 0.001$)						
Test for subgroup differences: $\chi^2_1 = 14.07$, df = 1 ($p < 0.001$)						

G. By risk of bias

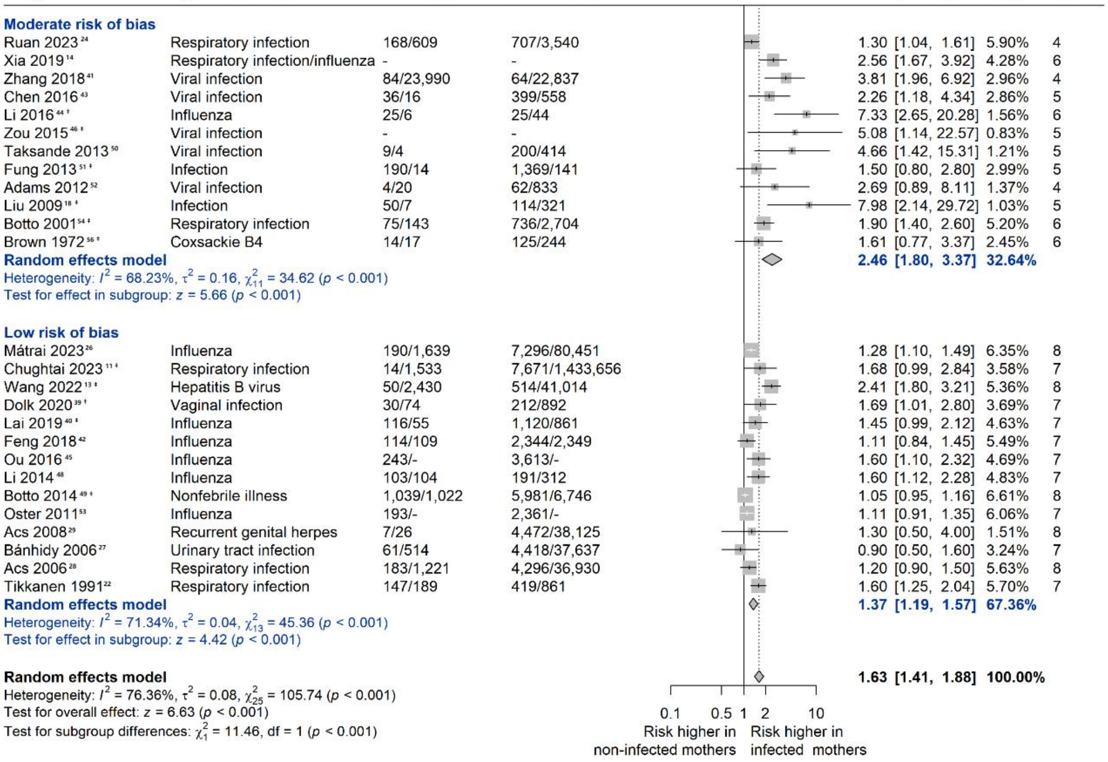

Study	Type of infection	Infected cases/non-cases	Non-infected cases/non-cases	Odds Ratio	OR† (95% CI)	Weight	Quality score
**Moderate risk of bias**							
Ruan 2023[24]	Respiratory infection	168/609	707/3,540		1.30 [1.04, 1.61]	5.90%	4
Xia 2019[14]	Respiratory infection/influenza	-	-		2.56 [1.67, 3.92]	4.28%	6
Zhang 2018[41]	Viral infection	84/23,990	64/22,837		3.81 [1.96, 6.92]	2.96%	4
Chen 2016[43]	Viral infection	36/16	399/558		2.26 [1.18, 4.34]	2.86%	5
Li 2016[44‡]	Influenza	25/6	25/44		7.33 [2.65, 20.28]	1.56%	6
Zou 2015[46‡]	Viral infection	-	-		5.08 [1.14, 22.57]	0.83%	5
Taksande 2013[50]	Viral infection	9/4	200/414		4.66 [1.42, 15.31]	1.21%	5
Fung 2013[51‡]	Infection	190/14	1,369/141		1.50 [0.80, 2.80]	2.99%	5
Adams 2012[52]	Viral infection	4/20	62/833		2.69 [0.89, 8.11]	1.37%	4
Liu 2009[18‡]	Infection	50/7	114/321		7.98 [2.14, 29.72]	1.03%	5
Botto 2001[54‡]	Respiratory infection	75/143	736/2,704		1.90 [1.40, 2.60]	5.20%	6
Brown 1972[56‡]	Coxsackie B4	14/17	125/244		1.61 [0.77, 3.37]	2.45%	6
**Random effects model**					**2.46 [1.80, 3.37]**	**32.64%**	
Heterogeneity: $I^2 = 68.23\%$, $\tau^2 = 0.16$, $\chi^2_{11} = 34.62$ ($p < 0.001$)							
Test for effect in subgroup: $z = 5.66$ ($p < 0.001$)							
**Low risk of bias**							
Mátrai 2023[26]	Influenza	190/1,639	7,296/80,451		1.28 [1.10, 1.49]	6.35%	8
Chughtai 2023[11‡]	Respiratory infection	14/1,533	7,671/1,433,656		1.68 [0.99, 2.84]	3.58%	7
Wang 2022[13‡]	Hepatitis B virus	50/2,430	514/41,014		2.41 [1.80, 3.21]	5.36%	8
Dolk 2020[39†]	Vaginal infection	30/74	212/892		1.69 [1.01, 2.80]	3.69%	7
Lai 2019[40‡]	Influenza	116/55	1,120/861		1.45 [0.99, 2.12]	4.63%	7
Feng 2018[42]	Influenza	114/109	2,344/2,349		1.11 [0.84, 1.45]	5.49%	7
Ou 2016[45]	Influenza	243/-	3,613/-		1.60 [1.10, 2.32]	4.69%	7
Li 2014[48]	Influenza	103/104	191/312		1.60 [1.12, 2.28]	4.83%	7
Botto 2014[49‡]	Nonfebrile illness	1,039/1,022	5,981/6,746		1.05 [0.95, 1.16]	6.61%	8
Oster 2011[53]	Influenza	193/-	2,361/-		1.11 [0.91, 1.35]	6.06%	7
Acs 2008[29]	Recurrent genital herpes	7/26	4,472/38,125		1.30 [0.50, 4.00]	1.51%	8
Bánhidy 2006[27]	Urinary tract infection	61/514	4,418/37,637		0.90 [0.50, 1.60]	3.24%	7
Acs 2006[28]	Respiratory infection	183/1,221	4,296/36,930		1.20 [0.90, 1.50]	5.63%	8
Tikkanen 1991[22]	Respiratory infection	147/189	419/861		1.60 [1.25, 2.04]	5.70%	7
**Random effects model**					**1.37 [1.19, 1.57]**	**67.36%**	
Heterogeneity: $I^2 = 71.34\%$, $\tau^2 = 0.04$, $\chi^2_{13} = 45.36$ ($p < 0.001$)							
Test for effect in subgroup: $z = 4.42$ ($p < 0.001$)							
**Random effects model**					**1.63 [1.41, 1.88]**	**100.00%**	
Heterogeneity: $I^2 = 76.36\%$, $\tau^2 = 0.08$, $\chi^2_{25} = 105.74$ ($p < 0.001$)							
Test for overall effect: $z = 6.63$ ($p < 0.001$)							
Test for subgroup differences: $\chi^2_1 = 11.46$, df = 1 ($p < 0.001$)							

H. By diagnosis timing (post-hoc analysis)

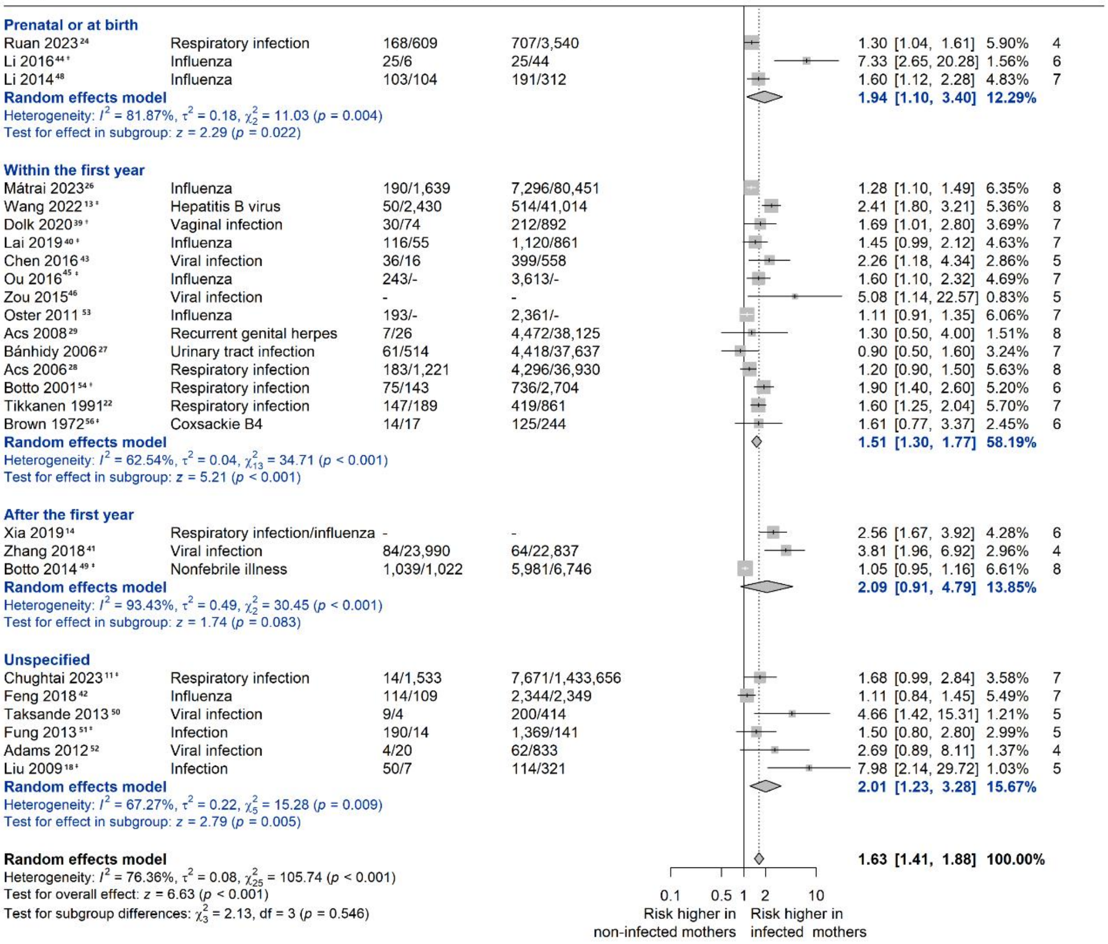

Study	Type of infection	Infected cases/non-cases	Non-infected cases/non-cases	Odds Ratio	OR (95% CI)	Weight	Quality score
**Prenatal or at birth**							
Ruan 2023[24]	Respiratory infection	168/609	707/3,540		1.30 [1.04, 1.61]	5.90%	4
Li 2016[44†]	Influenza	25/6	25/44		7.33 [2.65, 20.28]	1.56%	6
Li 2014[48]	Influenza	103/104	191/312		1.60 [1.12, 2.28]	4.83%	7
**Random effects model**					**1.94 [1.10, 3.40]**	**12.29%**	
Heterogeneity: $I^2 = 81.87\%$, $\tau^2 = 0.18$, $\chi^2_2 = 11.03$ ($p = 0.004$)							
Test for effect in subgroup: $z = 2.29$ ($p = 0.022$)							
**Within the first year**							
Mátrai 2023[26]	Influenza	190/1,639	7,296/80,451		1.28 [1.10, 1.49]	6.35%	8
Wang 2022[13‡]	Hepatitis B virus	50/2,430	514/41,014		2.41 [1.80, 3.21]	5.36%	8
Dolk 2020[39†]	Vaginal infection	30/74	212/892		1.69 [1.01, 2.80]	3.69%	7
Lai 2019[40‡]	Influenza	116/55	1,120/861		1.45 [0.99, 2.12]	4.63%	7
Chen 2016[43]	Viral infection	36/16	399/558		2.26 [1.18, 4.34]	2.86%	5
Ou 2016[45‡]	Influenza	243/-	3,613/-		1.60 [1.10, 2.32]	4.69%	7
Zou 2015[46]	Viral infection	-	-		5.08 [1.14, 22.57]	0.83%	5
Oster 2011[53]	Influenza	193/-	2,361/-		1.11 [0.91, 1.35]	6.06%	7
Acs 2008[29]	Recurrent genital herpes	7/26	4,472/38,125		1.30 [0.50, 4.00]	1.51%	8
Bánhidy 2006[27]	Urinary tract infection	61/514	4,418/37,637		0.90 [0.50, 1.60]	3.24%	7
Acs 2006[28]	Respiratory infection	183/1,221	4,296/36,930		1.20 [0.90, 1.50]	5.63%	8
Botto 2001[54‡]	Respiratory infection	75/143	736/2,704		1.90 [1.40, 2.60]	5.20%	6
Tikkanen 1991[22]	Respiratory infection	147/189	419/861		1.60 [1.25, 2.04]	5.70%	7
Brown 1972[56‡]	Coxsackie B4	14/17	125/244		1.61 [0.77, 3.37]	2.45%	6
**Random effects model**					**1.51 [1.30, 1.77]**	**58.19%**	
Heterogeneity: $I^2 = 62.54\%$, $\tau^2 = 0.04$, $\chi^2_{13} = 34.71$ ($p < 0.001$)							
Test for effect in subgroup: $z = 5.21$ ($p < 0.001$)							
**After the first year**							
Xia 2019[14]	Respiratory infection/influenza	-	-		2.56 [1.67, 3.92]	4.28%	6
Zhang 2018[41]	Viral infection	84/23,990	64/22,837		3.81 [1.96, 6.92]	2.96%	4
Botto 2014[49‡]	Nonfebrile illness	1,039/1,022	5,981/6,746		1.05 [0.95, 1.16]	6.61%	8
**Random effects model**					**2.09 [0.91, 4.79]**	**13.85%**	
Heterogeneity: $I^2 = 93.43\%$, $\tau^2 = 0.49$, $\chi^2_2 = 30.45$ ($p < 0.001$)							
Test for effect in subgroup: $z = 1.74$ ($p = 0.083$)							
**Unspecified**							
Chughtai 2023[11‡]	Respiratory infection	14/1,533	7,671/1,433,656		1.68 [0.99, 2.84]	3.58%	7
Feng 2018[42]	Influenza	114/109	2,344/2,349		1.11 [0.84, 1.45]	5.49%	7
Taksande 2013[50]	Viral infection	9/4	200/414		4.66 [1.42, 15.31]	1.21%	5
Fung 2013[51‡]	Infection	190/14	1,369/141		1.50 [0.80, 2.80]	2.99%	5
Adams 2012[52]	Viral infection	4/20	62/833		2.69 [0.89, 8.11]	1.37%	4
Liu 2009[18‡]	Infection	50/7	114/321		7.98 [2.14, 29.72]	1.03%	5
**Random effects model**					**2.01 [1.23, 3.28]**	**15.67%**	
Heterogeneity: $I^2 = 67.27\%$, $\tau^2 = 0.22$, $\chi^2_5 = 15.28$ ($p = 0.009$)							
Test for effect in subgroup: $z = 2.79$ ($p = 0.005$)							
**Random effects model**					**1.63 [1.41, 1.88]**	**100.00%**	
Heterogeneity: $I^2 = 76.36\%$, $\tau^2 = 0.08$, $\chi^2_{25} = 105.74$ ($p < 0.001$)							
Test for overall effect: $z = 6.63$ ($p < 0.001$)							
Test for subgroup differences: $\chi^2_3 = 2.13$, df = 3 ($p = 0.546$)							

Figure S3. Meta-analysis of any first trimester maternal infection for overall congenital heart defects in offspring, by subgroups: A) study region; B) study design; C) study setting; D) infection assessment method; E) adjustment for confounders or not; F) inclusion of pregnancy terminations or not; G) risk of bias; H) diagnosis timing (post-hoc analysis)

Forest plot presenting the association between any maternal infection during the first trimester of pregnancy and risk of congenital heart defects in the offspring by subgroups of study region, study design, study setting, infection assessment method, adjustment for confounders or not, inclusion of pregnancy terminations or not, and risk of bias. Pooled OR and their corresponding 95% CI were calculated using inverse variance weighted random-effects model. The quality score was calculated using the Newcastle-Ottawa Scale. Scores of 0-3, 4-6, and 7-9 are regarded as high, moderate, and low risk of bias, respectively. Main analyses were restricted to studies with low or moderate risk of bias. OR = odds ratio. CI = confidence interval.

†: Wang 2022 reports relative risk, others report odds ratio.

‡: In studies reporting associations for more than one infection, the infection variable with the highest prevalence ("Type of infection" column) was used as the quantitative summary for any infection in pooled analyses.

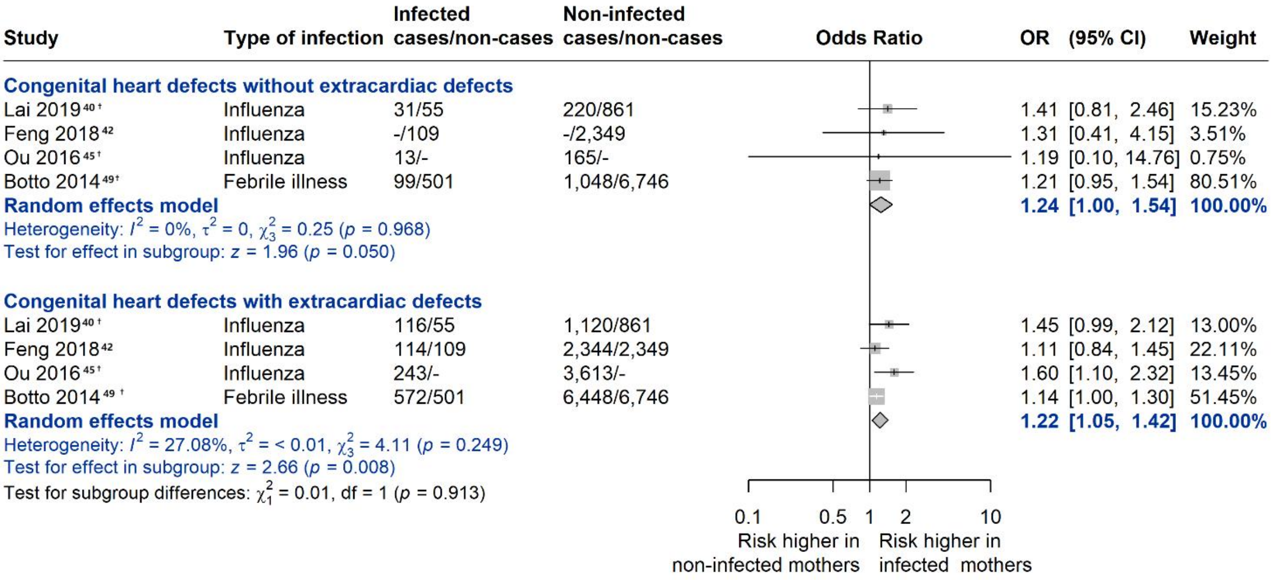

Figure S4. Meta-analysis of any first trimester maternal infection for congenital heart defects without extracardiac defects and with extracardiac defects among the same population

This is a post-hoc analysis on 4 studies with low or moderate risk of bias investigating CHD both with and without extracardiac defects. The forest plot presenting the association between any maternal infection during the first trimester of pregnancy and risk of congenital heart defects in the offspring. Pooled OR and their corresponding 95% CI were calculated using inverse variance weighted random-effects model. OR = odds ratio. CI = confidence interval.

†: In studies reporting associations for more than one infection, the infection variable with the highest prevalence ("Type of infection" column) was used as the quantitative summary for any infection in pooled analyses.

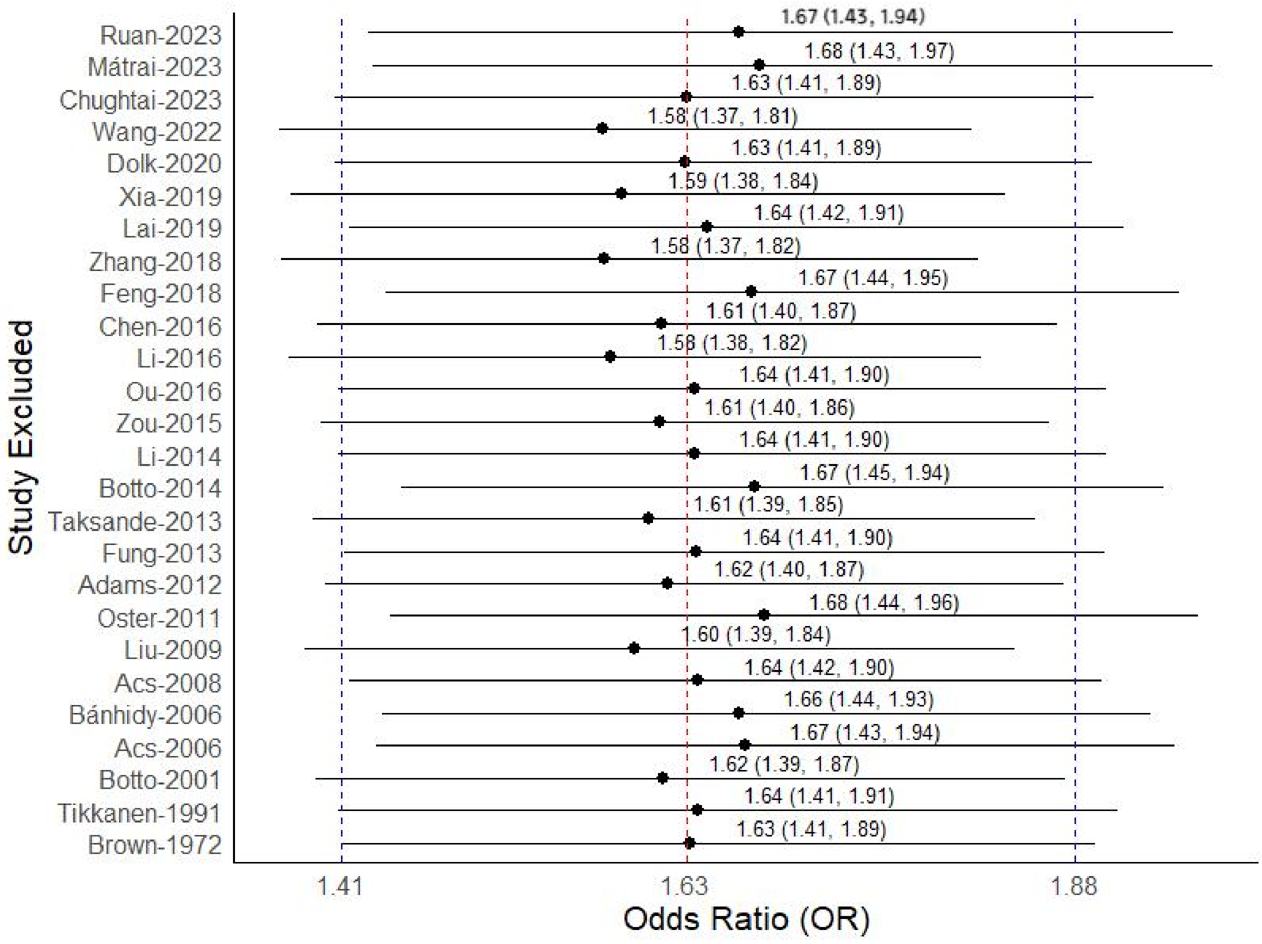

Figure S5. Leave-one-out sensitivity analysis

This analysis is based on the 26 studies with low or moderate risk of bias that investigated first-trimester maternal infection and overall congenital heart defects in offspring.

This analysis evaluates the influence of each individual study on the overall pooled effect size by iteratively removing one study at a time. All resulting estimates remained consistent with the main analysis (OR: 1.63; 95% CI: 1.41, 1.88).

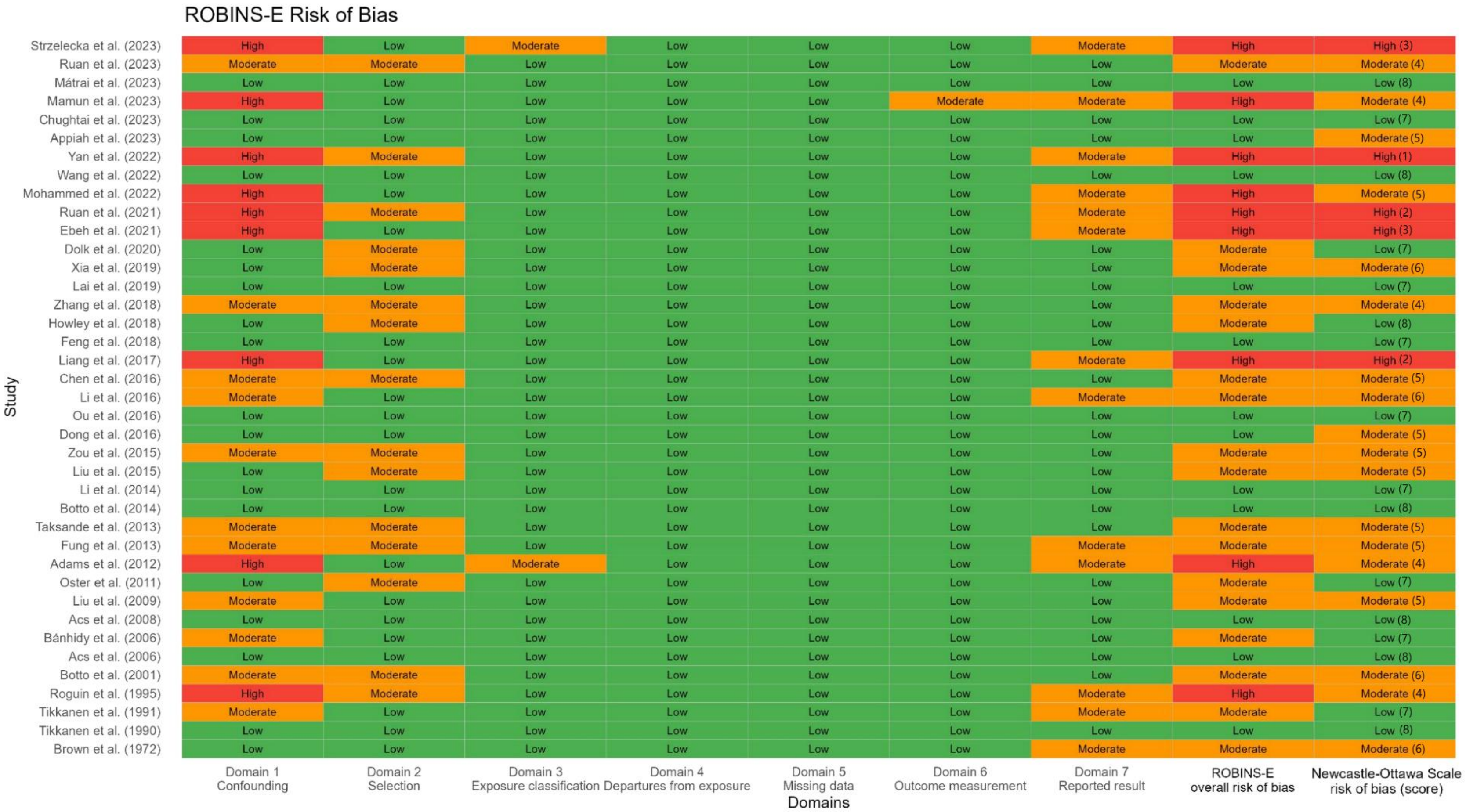

ROBINS-E Risk of Bias

Study	Domain 1 Confounding	Domain 2 Selection	Domain 3 Exposure classification	Domain 4 Departures from exposure	Domain 5 Missing data	Domain 6 Outcome measurement	Domain 7 Reported result	ROBINS-E overall risk of bias	Newcastle-Ottawa Scale risk of bias (score)
Strzelecka et al. (2023)	High	Low	Moderate	Low	Low	Low	Moderate	High	High (3)
Ruan et al. (2023)	Moderate	Moderate	Low	Low	Low	Low	Low	Moderate	Moderate (4)
Mátrai et al. (2023)	Low	Low	Low	Low	Low	Low	Low	Low	Low (8)
Mamun et al. (2023)	High	Low	Low	Low	Low	Moderate	Moderate	High	Moderate (4)
Chughtai et al. (2023)	Low	Low	Low	Low	Low	Low	Low	Low	Low (7)
Appiah et al. (2023)	Low	Low	Low	Low	Low	Low	Low	Low	Moderate (5)
Yan et al. (2022)	High	Moderate	Low	Low	Low	Low	Moderate	High	High (1)
Wang et al. (2022)	Low	Low	Low	Low	Low	Low	Low	Low	Low (8)
Mohammed et al. (2022)	High	Low	Low	Low	Low	Low	Moderate	High	Moderate (5)
Ruan et al. (2021)	High	Moderate	Low	Low	Low	Low	Moderate	High	High (2)
Ebeh et al. (2021)	High	Low	Low	Low	Low	Low	Moderate	High	High (3)
Dolk et al. (2020)	Low	Moderate	Low	Low	Low	Low	Low	Moderate	Low (7)
Xia et al. (2019)	Low	Moderate	Low	Low	Low	Low	Low	Moderate	Moderate (6)
Lai et al. (2019)	Low	Low	Low	Low	Low	Low	Low	Low	Low (7)
Zhang et al. (2018)	Moderate	Moderate	Low	Low	Low	Low	Low	Moderate	Moderate (4)
Howley et al. (2018)	Low	Moderate	Low	Low	Low	Low	Low	Moderate	Low (8)
Feng et al. (2018)	Low	Low	Low	Low	Low	Low	Low	Low	Low (7)
Liang et al. (2017)	High	Low	Low	Low	Low	Low	Moderate	High	High (2)
Chen et al. (2016)	Moderate	Moderate	Low	Low	Low	Low	Low	Moderate	Moderate (5)
Li et al. (2016)	Moderate	Low	Low	Low	Low	Low	Moderate	Moderate	Moderate (6)
Ou et al. (2016)	Low	Low	Low	Low	Low	Low	Low	Low	Low (7)
Dong et al. (2016)	Low	Low	Low	Low	Low	Low	Low	Low	Moderate (5)
Zou et al. (2015)	Moderate	Moderate	Low	Low	Low	Low	Low	Moderate	Moderate (5)
Liu et al. (2015)	Low	Moderate	Low	Low	Low	Low	Low	Moderate	Moderate (5)
Li et al. (2014)	Low	Low	Low	Low	Low	Low	Low	Low	Low (7)
Botto et al. (2014)	Low	Low	Low	Low	Low	Low	Low	Low	Low (8)
Taksande et al. (2013)	Moderate	Moderate	Low	Low	Low	Low	Low	Moderate	Moderate (5)
Fung et al. (2013)	Moderate	Moderate	Low	Low	Low	Low	Moderate	Moderate	Moderate (5)
Adams et al. (2012)	High	Low	Moderate	Low	Low	Low	Moderate	High	Moderate (4)
Oster et al. (2011)	Low	Moderate	Low	Low	Low	Low	Low	Moderate	Low (7)
Liu et al. (2009)	Moderate	Low	Low	Low	Low	Low	Low	Moderate	Moderate (5)
Acs et al. (2008)	Low	Low	Low	Low	Low	Low	Low	Low	Low (8)
Bánhidy et al. (2006)	Moderate	Low	Low	Low	Low	Low	Low	Moderate	Low (7)
Acs et al. (2006)	Low	Low	Low	Low	Low	Low	Low	Low	Low (8)
Botto et al. (2001)	Moderate	Moderate	Low	Low	Low	Low	Low	Moderate	Moderate (6)
Roguin et al. (1995)	High	Moderate	Low	Low	Low	Low	Moderate	High	Moderate (4)
Tikkanen et al. (1991)	Moderate	Low	Low	Low	Low	Low	Moderate	Moderate	Low (7)
Tikkanen et al. (1990)	Low	Low	Low	Low	Low	Low	Low	Low	Low (8)
Brown et al. (1972)	Low	Low	Low	Low	Low	Low	Moderate	Moderate	Moderate (6)

Figure S6. Post-hoc risk-of-bias assessment of 39 included studies using the Risk Of Bias In Non-randomized Studies - of Exposure tool

ROBINS-E assesses risk of bias across seven domains: confounding, selection of participants, classification of exposures, departures from intended exposures, missing data, measurement of outcomes, and selection of the reported result. Each domain was rated using three levels - low, moderate, and high risk of bias - based on the standard ROBINS-E criteria, considering the specific context of the present review. In particular, for the confounding domain, studies that adjusted for or matched on key factors such as maternal age were rated as low or moderate risk; those with no report control for confounding were rated as high risk. The overall rating reflects the highest domain-level risk in each study. This figure presents ROBINS-E assessments for all included studies as a supplementary evaluation. The results were not used to define study inclusion for main analyses but informed post-hoc sensitivity analyses.
